# Efficacy of emergency extracorporeal shock wave lithotripsy in the treatment of ureteral stones: a meta-analysis

**DOI:** 10.1186/s12894-023-01226-5

**Published:** 2023-04-04

**Authors:** Cheng-Xia Peng, Yi-Kai Lou, Li Xu, Guang-Hao Wu, Xie-Lai Zhou, Kang-Er Wang, Chun-Hua Ye

**Affiliations:** 1 Department of Urology, The Affiliated Hospital of Hangzhou Normal University, Hangzhou, 310015 China; 2 School of Medicine, Hangzhou Normal University, Hangzhou, 310016 China

**Keywords:** Ureteral stones, Emergency, Extracorporeal shock wave lithotripsy, Meta-analysis

## Abstract

**Objective:**

To compare the clinical efficiency and safety of emergency extracorporeal shock wave lithotripsy (eESWL) and delayed extracorporeal shock wave lithotripsy (dESWL) in the treatment of ureteral stones.

**Methods:**

Cochrane Library, PubMed, Google Scholar, and Web of Science were searched from January 1, 1992 to September 30, 2022, and all comparative studies involving eESWL and dESWL for ureteral calculi were included. Statistical analysis was performed using Review Manager 5.3 software. Funnel plot was used to evaluated publication bias.

**Results:**

A total of 9 articles involving 976 patients diagnosed with ureteral stones were included. The results showed that the stone-free rate (SFR) after four weeks was significantly higher in the eESWL group than in the dESWL group [relative risk (RR) = 1.22, 95% confidence interval (CI): 1.13–1.32, P < 0.01]. In subgroup analysis of different stone locations, proximal ureteral calculi [RR = 1.25, 95% CI: 1.14–1.38, P < 0.01] and mid-to-distal ureteral calculi [RR = 1.18, 95% CI: 1.03–1.34, P < 0.05] all showed a higher SFR in the eESWL group. eESWL significantly shortened the stone-free time(SFT) [mean difference (MD) = -5.75, 95% CI: -9.33 to -2.17, P < 0.01]. In addition, eESWL significantly reduced auxiliary procedures [RR = 0.53, 95% CI: 0.40–0.70, P < 0.01]. No significant difference in complications was found between the two groups [RR = 0.90, 95% CI: 0.69–1.16, P > 0.05].

**Conclusion:**

eESWL can significantly improve SFR, shorten SFT, and reduce auxiliary procedures.

**Supplementary Information:**

The online version contains supplementary material available at 10.1186/s12894-023-01226-5.

## Background

Renal colic (RC) caused by ureteral calculi is one of the most common emergencies encountered by urologists in clinical practice. Conservative treatment, extracorporeal shock wave lithotripsy (ESWL), ureteroscopic lithotripsy (URS), and laparoscopic or open surgery are the common methods for the treatment of ureteral calculi to relieve RC [1]. Usually, whether a ureteral stone can be spontaneously expelled depends on the size and shape of the stone, as well as its location in the ureter [2]. For stones > 6 mm in diameter, the chances of spontaneous expulsion are significantly reduced [1]. Conservative treatment is usually accompanied by recurrent RC, repeated emergency department visits, and loss of work ability, and long-term conservative treatment may be associated with complications such as infection, ureteral stricture, and renal function impairment [2].

Since its advent in the 1980s, ESWL has long been considered the first-choice treatment for upper urinary tract stones due to its simplicity, non-invasiveness, fewer complications, and low cost [3]. It is recommended by the European Association of Urology (EAU) guidelines for the management of urinary stones, which state that the three-month stone-free rate (SFR) is 82% for the proximal ureter, 3% for the middle ureter, and 74% for the distal ureter for ureteral calculi smaller than 2 cm [4, 5].

However, for patients with acute RC, conservative therapy such as spasmolysis and pain relief is performed first, while lithotripsy, whether ESWL or URS is often delayed. Delayed lithotripsy is often associated with recurrent RC, possible urinary tract infections, and azotaemia [6]. For patients with these conditions, treatment should focus on relieving pain and removing stones as soon as possible, while reducing complications. Kravchick and colleagues [7] conducted a randomized controlled trial (RCT) to investigate the efficacy of emergency extracorporeal shock wave lithotripsy (eESWL) in ureteral stones and concluded that ESWL within 48–72 h after RC relieves obstruction and pain more quickly, which is a safe and effective treatment. Tombal et al. [8] found that ESWL within 6 h was associated with faster stone clearance and shorter hospital stays. Choi et al. [9] showed that compared to delayed extracorporeal shock wave lithotripsy (dESWL), patients receiving eESWL experienced a significantly higher rate of treatment success, quicker stone expulsion, and fewer ESWL sessions. A growing number of studies have confirmed that eESWL is efficacious and safe for treating ureteral stones; therefore, the present study sought to evaluate the role of eESWL in ureteral stone expulsion through a comprehensive meta-analysis.

## Methods

### Search strategy and study selection

This meta-analysis was conducted according to Preferred Reporting Items for Systematic Reviews and Meta-Analyses (PRISMA) guidelines [10]. Relevant studies were searched on PubMed, Web of Science, Cochrane Library, and Google Scholar from January 1, 1992 to September 30, 2022, without language restriction. Search terms included: “emergency”, “urgency”, “extracorporeal shockwave lithotripsy”, “ureteral stones”, “ureteral calculi”, “ureteral urolithiasis”, and “ureteric stones”. Our study protocol was registered on PROSPERO (No. CRD42023407392).

The inclusion criteria were as follows. (1) Comparative study of eESWL and dESWL in the treatment of ureteral calculi. (2) Stone characteristics: unilateral, single ureteral calculi, stone diameter < 2 cm. (3) Intervention measures: the experimental group was eESWL (ESWL within 48–72 h of RC attack) and the control group was dESWL (ESWL after 48–72 h of RC attack). (4) Outcome measures: at least one of the SFR of the proximal ureteral calculi, SFR of the mid-to-distal ureteral calculi, overall SFR, stone-free time (SFT), complications, and auxiliary procedures. Exclusion criteria were: (1) case reports, reviews, editorial comments, and conference abstracts; (2) duplicate publications; (3) data not available or extractable.

### Data extraction and quality assessment

Two researchers independently performed the literature search and assessed the eligibility of studies based on the inclusion and exclusion criteria by reading the titles and abstracts. Any discrepancies were resolved through a consensus discussion with a third researcher. The following information was extracted: (1) general data, including the first author, publication time, country, and type of literature; (2) patient characteristics, including the number of experimental group and control group, intervention measures, stone size, stone location; (3) outcome measures: SFR, SFT(day), the incidence of complications, and auxiliary procedures. We performed subgroup analysis according to the location of calculi, including proximal ureteral calculi group and mid-to-distal ureteral calculi group. The Newcastle-Ottawa Scale (NOS) was used to evaluate the quality of non-randomised studies. NOS scores were assessed on a 9-point scale. A score of 0–3, 4–6, and 7–9 represents a low, moderate, and high quality, respectively. The Cochrane Collaboration tool was used to evaluate the risk bias of RCTs as follows: low, unclear, and high risk of bias.

### Statistical analysis

Review Manager 5.3 software was used for statistical analysis (Cochrane Collaboration, Oxford, United Kingdom). Relative risk (RR) was used as the effective index for dichotomous variables, whereas mean difference (MD) was used for continuous variables. The results were expressed as a 95% confidence interval (CI). The statistical significance level was set at P < 0.05. χ^2^ and I^2^ were used to test the heterogeneity among the results of each study. A random-effects model was adopted for pooled analysis when statistical heterogeneity was found (I^2^ ≥ 50%, P ≤ 0.1), while a fixed-effects model was adopted when no significant heterogeneity was detected (I^2^ < 50% and P > 0.1). The ultimate results are presented in forest plots. And publication bias was evaluated through funnel plot. .

## Results

### Search results and study characteristics

**Fig. 1:**
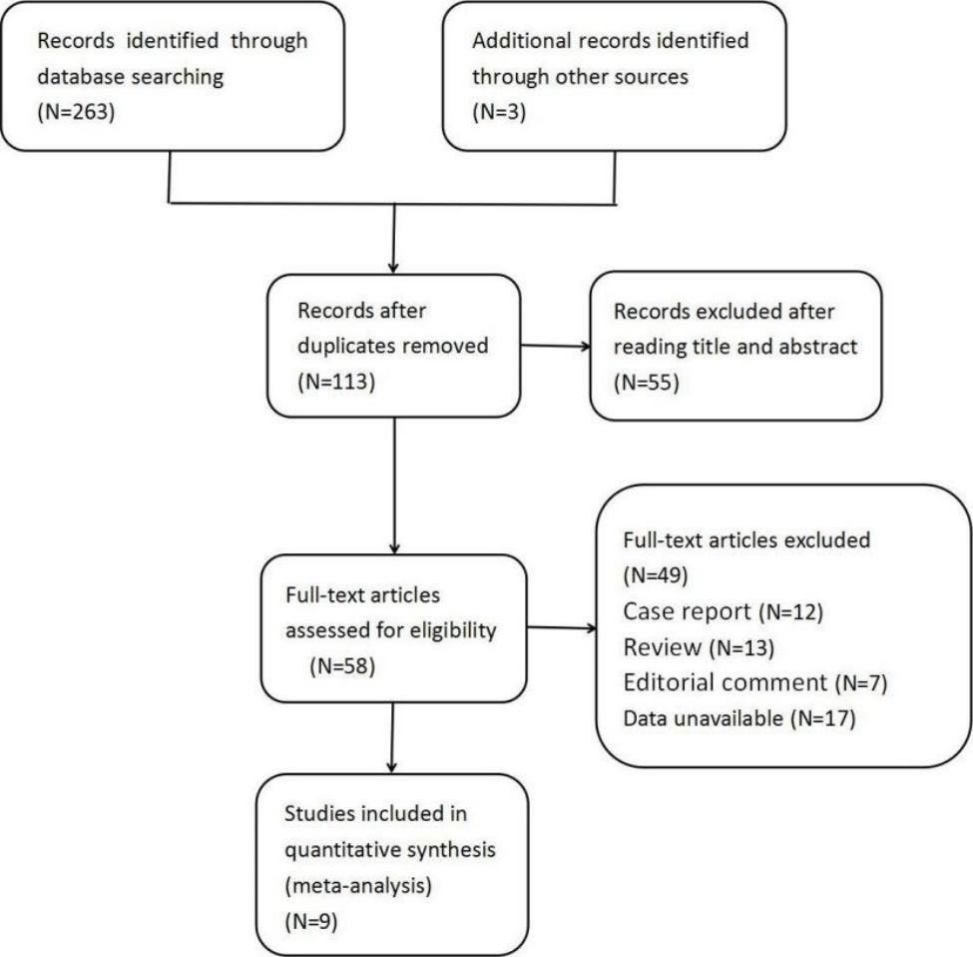
Flowchart of study selection

A total of 9 articles were retrieved, including 5 RCTs and 4 retrospective studies. The flow diagram of the literature screening process is shown in Fig. 1. The quality evaluation of the studies are shown in Table 1. All studies were of high quality. A total of 976 patients were included in the study, of whom 488 patients underwent ESWL within 48–72 h of the onset of RC as an intervention. The remaining 488 patients underwent ESWL after 48–72 h, and the mean follow-up time for all patients was 4 weeks. A summary of the included studies is shown in Table 2.

**Table 1:** Newcastle-Ottawa Scale and Cochrane Collaboration tool for quality evaluation of the studies

		Selection				Comparability	Outcome			
Study	Design	Representativeness of exposed cohort	Selective of nonexposed Cohort	Ascertainment of exposure	Outcome not present at start		Assessment of outcome	Adequate follow-up length	Adequacy of follow-up	Total
Choi 2012	R	*	*	*	*	*	*	*		7
Cornelius 2020	R	*	*	*	*	**	*	*	*	9
Joshi 1999	R	*	*	*	*	*	*	*	*	8
Seitz 2005	R	*	*	*	*	*		*	*	7
		Random sequence generation	Allocation concealment	Blinding of participant and personnel	Blinding of outcome assessment	Incomplete outcome data	Selective reporting	Other bias		
Bucci 2018	RCT	Low	Unclear	Low	Low	Low	Low	Unclear		
Kravchick 2005	RCT	Low	Unclear	Unclear	Unclear	Low	Low	Low		
Kumar 2010	RCT	Low	Unclear	Low	Low	Low	Low	Low		
Tombal 2005	RCT	Unclear	Unclear	Unclear	Low	Low	Low	Low		
Uguz 2012	RCT	Low	Low	Low	Low	Low	Low	Unclear		

* signifies score, RCT: Randomised controlled trial, R: Retrospectively study

**Table 2:** Characteristics of studies

Study	Country	Design	Therapy in the experimental group	Therapy in the control group	Sample size: experiment/control	Stone size: experiment/control, mm	Location of stone	
							Proximal ureter	Mid-to-distal ureter
Bucci 2018 [11]	Italy	RCT	ESWL within 12 h	dESWL	36/34	9.03/10.68	25	45
Choi 2012 [9]	Korea	R	ESWL within 48 h	dESWL	153/126	7.6 ± 2.5/8.3 ± 3.1	183	96
Cornelius 2020 [12]	Italy	R	ESWL within 48 h	dESWL	52/52	7.1 ± 1.9/7.2 ± 1.9	80	24
Joshi 1999 [13]	UK	R	ESWL within 48 h	dESWL	16/40	8.2/9.3	37	19
Kravchick 2005 [7]	Israel	RCT	ESWL within 48 to 72 h	dESWL	25/28	7.4 ± 2.4/6.9 ± 2.4	53	NA
Kumar 2010 [14]	India	RCT	ESWL within 48 h	dESWL	80/80	7.3 ± 1.5/7.5 ± 1.7	160	NA
Seitz 2005 [15]	Austria	R	ESWL within 48 h	dESWL	44/47	8.0 ± 2.4/8.1 ± 2.1	91	NA
Tombal 2005 [8]	Belgium	RCT	ESWL within 6 h	dESWL	50/50	6.38/4.8	46	54
Uguz 2012 [16]	Turkey	RCT	ESWL within 24 h	dESWL	32/31	8.1 ± 3.2/8.8 ± 2.9	41	22

RCT: Randomised controlled trial, R: Retrospectively study, NA: not available, ESWL: emergency extracorporeal shock wave lithotripsy, dESWL: delayed extracorporeal shock wave lithotripsy

### Stone-free rate

**Fig. 2:**
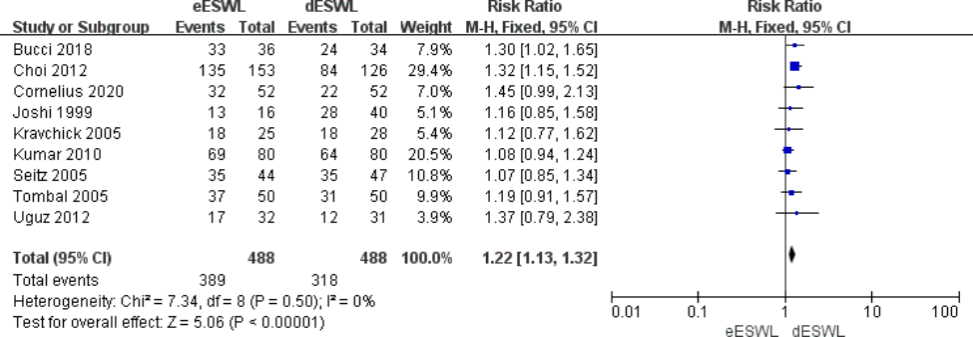
Forest plot of overall SFR

**Fig. 3:**
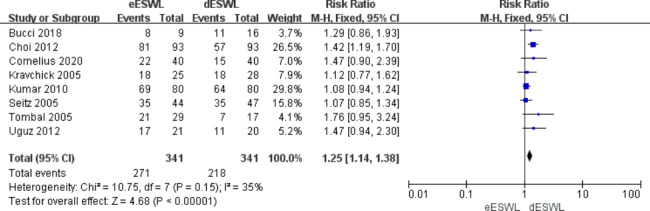
Forest plot of SFR for proximal ureteral calculi

**Fig. 4:**
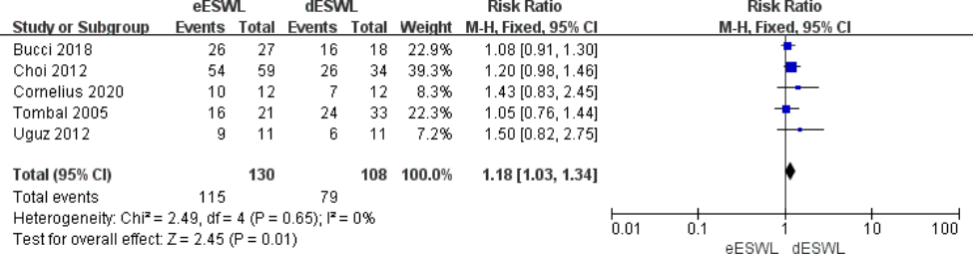
Forest plot of SFR for mid-to-distal ureteral calculi

The overall SFR was reported in all included studies, and eESWL increased the SFR compared with the control group [RR = 1.22, 95% CI: 1.13–1.32, I^2^ = 0%, P < 0.01] (Fig. 2). The SFR for proximal ureteral calculi was reported in eight of the articles, and the results showed that eESWL significantly improved SFR [RR = 1.25, 95% CI: 1.14–1.38, I^2^ = 35%, P < 0.01] (Fig. 3). Five studies reported SFRs for the mid-to-distal ureteral calculi, and the results also showed a higher SFR with eESWL [RR = 1.18, 95% CI: 1.03–1.34, I^2^ = 0%, P < 0.05] (Fig. 4).

### Stone-free time

**Fig. 5:**
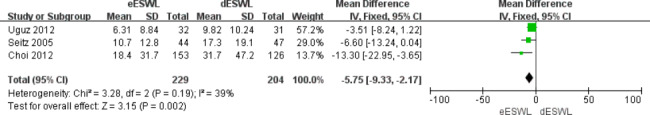
Forest plot of SFT

Stone free time was reported in three articles, and the results showed that the time required for stone expulsion was significantly shorter in eESWL group than in dESWL group [MD = -5.75, 95% CI (-9.33, -2.17), I^2^ = 39%, P < 0.01] (Fig. 5).

### Auxiliary procedure

**Fig. 6:**
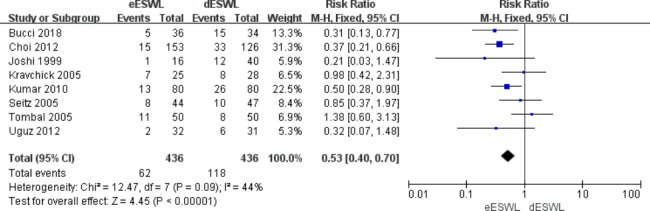
Forest plot of auxiliary procedure

Residual stones ≤ 4 mm in diameter are called insignificant residual stones, and additional ureteroscopic lithotripsy is performed as an auxiliary procedure to expel the stones if the diameter of residual stones after surgery is > 4 mm. Eight studies reported stone removal by the auxiliary procedure. The results showed that the rate of auxiliary procedure required by eESWL was significantly lower than that required by dESWL [RR = 0.53, 95% CI: 0.40–0.70, I^2^ = 44%, P < 0.01] (Fig. 6).

### Complications

**Fig. 7:**
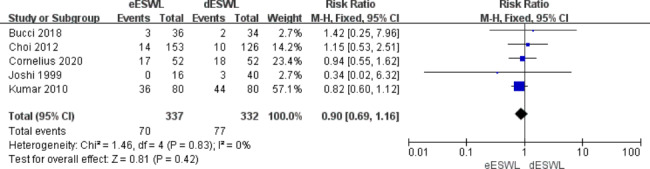
Forest plot of complications after ESWL

Postoperative complications after ESWL include recurrence of RC, fever, steinstrasse formation, azotaemia, and perirenal hematoma. The complication rate was reported in five articles, and the results showed that eESWL had a lower complication rate than dESWL [RR = 0.90, 95% CI: 0.69–1.16, I^2^ = 0%, P > 0.05] (Fig. 7), but no statistically significant was detected between the two methods.

### Publication bias

**Fig. 8:**
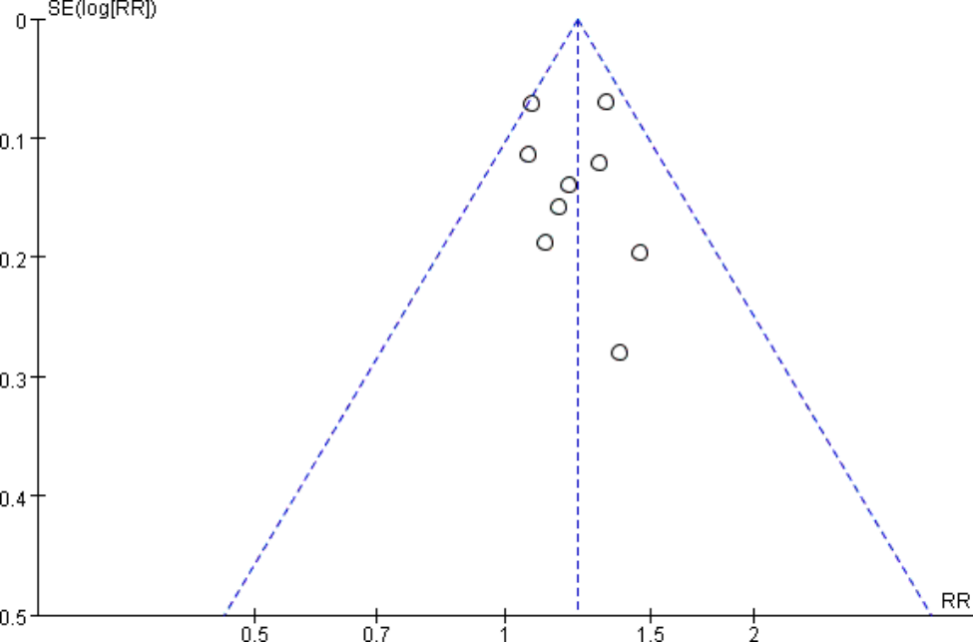
Funnel plot of overall SFR

No significant asymmetry was observed in funnel plots, which indicated no publication bias (Fig. 8).

## Discussion

The rationale for performing eESWL is mainly based on the finding that ureteral mucosal oedema starts after 24–48 h of stone obstruction, and progresses over time [17]. Therefore, ureteral mucosal oedema is closely related to the development of stone obstruction. A previous study demonstrated morphologic changes of the mucosa in the stone bed after 48 h, such as a marked increase in hyperplasia and mitotic activity in histologic examinations [18]. This gradual increase in ureteral mucosal oedema prevents luminal distension and the formation of fluid interfaces, impeding adequate delivery of shock wave energy, which decreases fragmentation and expulsion of stones [19]. Furthermore, Cummins et al. [20] showed that the duration after symptom onset was the most important predictor of ureteral stone removal. Therefore, the rationale for applying eESWL to treat RC caused by ureteral calculi is to achieve maximal SFR before the development or progression of peripheral mucosal oedema.

Herein, we conducted a systematic review and meta-analysis on the efficacy of eESWL and dESWL in the treatment of ureteral stones based on nine comparative clinical studies with 4 weeks of follow-up. This study compared the short-term follow-up results of patients who underwent eESWL and dESWL, which showed no significant difference in the incidence of complications. However, the eESWL group had higher SFR, fewer SFT, and reduced auxiliary procedures.

SFR after lithotripsy is an important reference for surgical results. Our study showed that eESWL significantly increased the SFR (P < 0.01, Fig. 2). Both proximal and mid-to-distal ureteral calculi showed a higher SFR (P < 0.05, Figs. 3 and 4). Tombal et al. [8] showed that eESWL was an efficient treatment for stones, with significantly higher stone clearance in patients with proximal calculi than in those with distal calculi, improving the success rate by more than 35%. Choi et al. [9] showed that eESWL is a reliable and efficient way to manage urinary stones, particularly proximal ureteral stones. Arrabal-Martin et al. [21] showed that the success of ESWL was comparable to ureteroscopy in proximal calculi. The reason may be that distal ureteral calculi are greatly affected by the bowel and pelvis, which can disturb the localisation of the target stone and transmission of shock waves to the target stone [22].

SFT after lithotripsy can also be used as a reference for surgical outcomes. Our study showed that eESWL significantly shortened the time required for stone expulsion (P < 0.01, Fig. 5). ESWL does not immediately achieve a stone-free status and may take some time to eliminate fragmented ureteral stones depending on various factors, such as the size and location of the stone, degree of stone impaction, and degree of ureteral mucosal oedema [23]. eESWL is performed to maximise stone clearance when ureteral mucosal oedema reaches the apex. Seitz et al. [15] showed that eESWL required significantly fewer shock sessions than dESWL.

Meanwhile, the current study showed that the need for auxiliary procedures after eESWL was much lower than after dESWL (P < 0.01, Fig. 6). This can be explained by the above pathophysiological principles that oedema and hyperplasia of the ureteral mucosa due to stone obstruction limit the luminal distension and formation of fluid interfaces [19]. This not only reduces the fragmentation rate after ESWL but also compromises stone clearance, simultaneously increasing the sessions of ESWL and the need for ureteroscopic lithotripsy.

Our study found no statistically significant difference in complications between the eESWL and dESWL groups. Generally, complications after ESWL are short-term and mild, the most common of which are RC, haematuria, urinary tract infection, and perirenal hematoma [24]. Kumar et al. [14] showed a slightly higher incidence of haematuria in the dESWL group compared with the eESWL group (41.3 vs. 38.8%, P < 0.05) and a higher rate of steinstrasse formation (12.5 vs. 6.25%, P < 0.05). Bucci et al. [11] reported a case of acute pyelonephritis due to postoperative steinstrasse in the dESWL group who underwent emergent double-J tube placement and intravenous antibiotics. Blackwell et al. [25] conducted a study that included 10,301 patients hospitalised for acute ureteral obstruction and found that early intervention reduced mortality by 0.16% compared with delayed intervention; they concluded that early intervention reduced patient mortality in some way.

This study has some limitations. First, not all of the included articles were RCTs, which reduced the quality of the included articles. Second, factors affecting lithotripsy, such as stone composition and distance from stone to skin, were not performed in the subgroup analysis, which may lead to biased results. Third, some studies used kidney-ureter-bladder X-ray rather than computed tomography to assess residual stones, and different authors had different definitions of stone-free status, which may also lead to biased results. Finally, the follow-up time was too short.

## Conclusion

In summary, eESWL is a safe and effective treatment for ureteral calculi, which can significantly improve the SFR, shorten the SFT, and reduce auxiliary procedures.

## Electronic supplementary material

Below is the link to the electronic supplementary material.

Additional File 1: Bucci 2018

Additional File 2: Choi 2012

Additional File 3: Cornelius 2020

Additional File 4: Joshi 1999

Additional File 5: Kravchick 2005

Additional File 6: Kumar 2010

Additional File 7: Seitz 2005

Additional File 8: Tombal 2005

Additional File 9: Uguz 2012

## Data Availability

All data generated or analysed during this study are included in this published article [and its supplementary information files].

## Abbreviations

ESWL: extracorporeal shock wave lithotripsy
eESWL: emergency extracorporeal shock wave lithotripsy
dESWL: delayed extracorporeal shock wave lithotripsy
SFR: stone-free rate
SFT: stone-free time
RR: relative risk
CI: confidence interval
MD: mean difference
RC: renal colic
URS: ureteroscopic lithotripsy
EAU: European Association of Urology
RCT: randomized controlled trial
PRISMA: Preferred Reporting Items for Systematic Reviews and Meta-Analyses
NOS: Newcastle-Ottawa Scale
