# Supplementary material for: Efficacy of emergency extracorporeal shock wave lithotripsy in the treatment of ureteral stones: a meta-analysis
Source: BMC Urol. 2023 Apr 4;23:56. doi: 10.1186/s12894-023-01226-5 (PMC10074806; doi:10.1186/s12894-023-01226-5)
Supplement: Supplementary file 1 — Additional File 1: Bucci 2018 [file 12894_2023_1226_MOESM1_ESM.pdf]

COPYRIGHT© 2018 EDIZIONI MINERVA MEDICA

This document is protected by international copyright laws. No additional reproduction is authorized. It is permitted for personal use to download and save only one file and print only one copy of this Article. It is not permitted to make additional copies (either sporadically or systematically, either printed or electronic) of the Article for any purpose. It is not permitted to distribute the electronic copy of the article through online internet and/or intranet file sharing systems, electronic mailing or any other means which may allow access to the Article. The use of all or any part of the Article for any Commercial Use is not permitted. The creation of derivative works from the Article is not permitted. The production of reprints for personal or commercial use is not permitted. It is not permitted to remove, cover, overlay, obscure, block, or change any copyright notices or terms of use which the Publisher may post on the Article. It is not permitted to frame or use framing techniques to enclose any trademark, logo, or other proprietary information of the Publisher.

© 2018 EDIZIONI MINERVA MEDICA
Online version at http://www.minervamedica.it

Minerva Urologica e Nefrologica 2018 October;70(5):526-33
DOI: 10.23736/S0393-2249.18.03084-9

ORIGINAL ARTICLE

Emergency extracorporeal shockwave lithotripsy as opposed to delayed shockwave lithotripsy for the treatment of acute renal colic due to obstructive ureteral stone: a prospective randomized trial

Stefano BUCCI [1], Paolo UMARI [1] *, Michele RIZZO [1], Nicola PAVAN [1], Giovanni LIGUORI [1], Fabio BARBONE [2], Carlo TROMBETTA [1]

[1]Department of Urology, Cattinara Hospital, University of Trieste, Trieste, Italy; [2]IRCCS Burlo Garofolo, University of Trieste, Trieste, Italy

*Corresponding author: Paolo Umari, Department of Urology, Cattinara Hospital, University of Trieste, Trieste, Italy.
E-mail: paoloumari@gmail.com

ABSTRACT

BACKGROUND: The aim of this study was to assess the efficacy of emergency extracorporeal shockwave lithotripsy (eSWL) as first-line treatment in patients with acute colic due to obstructive ureteral stone.
METHODS: Seventy-four patients were randomized to emergency SWL within 12 hours (eSWL group) and deferred SWL later than 3 days (dSWL group). Follow-up included ultrasound, KUB (kidney-ureter-bladder) radiography and CT (computed tomography) scan at 24 hours, 7 days, 1 and 3 months from the treatment. When necessary, repeated SWL (re-SWL) or ureteroscopy (auxiliary-URS) was performed. Preoperative and postoperative data were compared and stone free rates (SFR) and efficiency quotients (EQ) were evaluated. Analyses were performed using SAS software.
RESULTS: Complete data of 70 patients were collected. 36 underwent eSWL and 34 dSWL. The mean patient age was 48.7. Mean stone size was 9.8 mm (CI 95%: 8.9-10.8). 25 (35.7%) were proximal and 45 (64.3%) distal. Mean SWL energy was 19.2 kV (CI 95%: 18.5-19.9) and mean number of shocks was 2657 (CI 95%: 2513-2802). eSWL patients needs less auxiliary-URS than dSWL patients (13.9% *vs.* 44.1%, P=0.039) and less re-SWL sessions (8.3% *vs.* 32.4%, P=0.093). SFR at 24 hours was 52.8% and 11.8% (P<0.001) and the EQ at 3 months was 79.1% and 57.5% in the eSWL and dSWL group respectively. Patients from the dSWL group spent more time in the hospital (2.21 *vs.* 1.36 days, P=0.046) and complication rates between the two groups were similar.
CONCLUSIONS: eSWL is a safe procedure and delivers high SFR even within 24 hours especially for ≤10 mm stones. It is able to reduce the number of auxiliary procedures and hospitalization.

(*Cite this article as:* Bucci S, Umari P, Rizzo M, Pavan N, Liguori G, Barbone F, *et al.* Emergency extracorporeal shockwave lithotripsy as opposed to delayed shockwave lithotripsy for the treatment of acute renal colic due to obstructive ureteral stone: a prospective randomized trial. Minerva Urol Nefrol 2018;70:526-33. DOI: 10.23736/S0393-2249.18.03084-9)

KEY WORDS: Extracorporeal shockwave therapy - Lithotripsy - Ureteral calculi - Renal colic.

Urinary stone disease is the predominant cause of hospitalization in urological departments with a prevalence that vary from 1 to 20%.[1] Half of the patients who present with a renal colic will have a recurrence within 5 years after the first episode.[2, 3]

Treatment options include drug therapy, decompression of obstructed collecting system (ureteral stent, nephrostomy tube), extracorporeal shockwave lithotripsy (SWL), ureteroscopy (URS) and open or laparoscopic surgery.[4-6] However the European and US guidelines for

COPYRIGHT© 2018 EDIZIONI MINERVA MEDICA

This document is protected by international copyright laws. No additional reproduction is authorized. It is permitted for personal use to download and save only one file and print only one copy of this Article. It is not permitted to make additional copies (either sporadically or systematically, either printed or electronic) of the Article for any purpose. It is not permitted to distribute the electronic copy of the article through online internet and/or intranet file sharing systems, electronic mailing or any other means which may allow access to the Article. The use of all or any part of the Article for any Commercial Use is not permitted. The creation of derivative works from the Article is not permitted. The production of reprints for personal or commercial use is not permitted. It is not permitted to remove, cover, overlay, obscure, block, or change any copyright notices or terms of use which the Publisher may post on the Article. It is not permitted to frame or use framing techniques to enclose any trademark, logo, or other proprietary information of the Publisher.

urolithiasis reccomend MET (Medical Expulsive Therapy) with alpha blockers as first-line therapy for small (≤10 mm) ureteral stones in selected patients, its benefits seem still unclear with such conflicting data in the current literature.[7]

The SWL monotherapy for ureteral stones is safe, highly effective and mostly cost-effective.[8] Usually the treatment of ureteral stones with SWL or endoscopy is deferred for at least 3-4 weeks to await spontaneous passage. The spontaneous expulsion depends essentially on the size of the stone and decreases exponentially above 5 mm.[9]

Very few studies have addressed the role of immediate use of SWL whitin 6-72 hours for the treatment of patients who present with renal colic.[10-13] The rationale for emergency SWL (eSWL) is to achieve maximum stone clearance in the shortest possible time before ureteric edema formation.[14] Moreover it allows early detection of lithotripsy failure which could be treated with auxiliary procedures.[15]

Few studies show that eSWL reduces the incidence of auxiliary ureteroscopy procedures and hospital stay in patients with ureteral calculi.[16, 17] A comparative retrospective analysis has shown that eSWL has less morbidity and is more effective than nephrostomy placement or ureteral stenting.[18]

The aim of this study was to evaluate the efficacy of emergency SWL (eSWL) as first-line treatment in patients with acute colic due to an obstructive ureteral stone and to compare it with delayed SWL (dSWL) in terms of stone-free rate (SFR), efficiency quotient (EQ), hospital stay and complication rates.

Materials and methods

This study included patients admitted to our department with an episode of ureteric colic due to obstructive ureteral stone who underwent SWL treatment over a period of 14 months. The inclusion criteria were: no previous active treatment for stone disease, solitary, radiopaque or detected by ultrasound ureteral stone from 5 to 20 mm and normal renal function without evidence of urinary infection. Pregnant women, patients taking anticoagulants or antiplatelet medications, patients with coagulation disorders, solitary kidney, ureteral pathology, severe hydronephrosis (grade 3 and 4) and perirenal urinoma were excluded from the study.

Seventy-four patients matched all criteria and were enrolled in the study. They were randomly allocated in two groups using a block randomization method. Complete data of 70 patients were collected. Thirty patients were treated within 12 hours (eSWL group) and 34 patients later than 3 days from the time of admission (dSWL group). Four patients were excluded from the study because of spontaneous passage of the stone before the scheduled SWL session (1 from the eSWL group and 3 from the dSWL group).

The patients were previously informed about all available treatment modalities and their potential complications. The diagnosis of a ureteric stone was by ultrasonography and KUB (Kidney-Ureter-Bladder) radiography; when in question a CT (computed tomography) scan was performed. Proximal and distal calculi were defined by their position above or below the iliac vessels, respectively. Electrocardiography, urine dipstick and blood test including coagulation assay were performed in all cases before treatment. From the time of admission none of the patients had taken MET either before or after the SWL treatment.

All the procedures were performed by two expert urologist (>2000 procedures) from a dedicated stone unit team with the same electrohydraulic lithotripter (HMT Lithotron Lits172) at the frequency of 1 Hz. Upper ureteral stones were fragmented in supine position, while mid-ureter and distal calculi required prone position. The localization of the stone was achieved during the expiratory phase of the respiratory cycle with either fluoroscopy or ultrasound. Moreover, intravenous sedation with Petidine (1 mg/kg body weight) was performed in order to decrease the amplitude of the respiratory cycles and obtain a good analgesia. To maximize energy delivery to the stone, ultrasound gel was used as a coupling medium. The treatment started with low-energy shockwaves and increased gradually to allow the patient to adapt to the procedure. The treatment terminated when a sufficient stone fragmentation was observed.

COPYRIGHT© 2018 EDIZIONI MINERVA MEDICA

This document is protected by international copyright laws. No additional reproduction is authorized. It is permitted for personal use to download and save only one file and print only one copy of this Article. It is not permitted to make additional copies (either sporadically or systematically, either printed or electronic) of the Article for any purpose. It is not permitted to distribute the electronic copy of the article through online internet and/or intranet file sharing systems, electronic mailing or any other means which may allow access to the Article. The use of all or any part of the Article for any Commercial Use is not permitted. The creation of derivative works from the Article is not permitted. The production of reprints for personal or commercial use is not permitted. It is not permitted to remove, cover, overlay, obscure, block, or change any copyright notices or terms of use which the Publisher may post on the Article. It is not permitted to frame or use framing techniques to enclose any trademark, logo, or other proprietary information of the Publisher.

Patients were reviewed at 24 hours, 7 days, 1 and 3 months after each SWL session with ultrasound and KUB radiography to assess stone fragmentation and hydronephrosis. A CT scan was performed when the previous examinations were inconclusive. When necessary, auxiliary procedures as repeated SWL (re-SWL) or ureteroscopy (URS) were performed. The stone free status was defined as no residual fragments visible on follow-up imaging. The mean efficiency quotient (EQ) was calculated according to the formula of Denstedt *et al.*: 100% stone-free / (100% + percent retreatment + percent auxiliary procedures).[10] Hospital stay was defined as the time from patient admission to our department until discharged plus the time spent for eventual auxiliary procedures (re-SWL or URS). Complication rates were described according to the Clavien-Dindo classification.

Analyses were performed using SAS software. To compare continuous variables between the two groups the Student *t*-test was used. To assess the difference in categorical variables χ^2 test was used.

All procedures performed in the study were in accordance with the ethical standards of the Institutional and/or national research committee and with the 1964 Helsinki declaration and its later amendments or comparable ethical standards. Informed consent was obtained from all individual participants included in the study.

Results

The mean patient age was 48.7 years (95% CI: 40.4-50.0). Male/female ratio was 44/26 and left/right ratio was 30/40. Twenty-five stones (35.7%) were located in the upper ureter and 45 stones (64.3%) in the distal ureter. Mean stone size was 9.8 mm (95% CI: 8.9-10.8) and was higher for proximal compared to distal stones (12.9 *vs.* 8.1 mm). Hydronephrosis (grade 1 and 2) was present in 68 (97.1%) patients. All the stones were radioopaque or visible on ultrasound. 66 patients were treated as outpatients and 4 were kept in hospital overnight (3 from eSWL and 1 from dSWL group). In the dSWL group the mean time to treat was 8.3 days (95% CI: 6.9-9.1). SWL treatment lasted on average 44 minutes (95% CI: 33.8-60.5). No treatment was interrupted because of poor tolerance. The mean maximum energy was 19.2 kV (95% CI: 18.5-19.9). The mean shockwave number was 2657 (95% CI: 2513-2802). Regarding spontaneous stone passage before treatment, one patient from eSWL group passed a 6.5 mm stone and the mean expelled stone size of the three patients from the dSWL group was 7.3 mm. Patient characteristics are reported in Table I.

The stone free rate (SFR) in the eSWL and dSWL groups were 52.8% *vs.* 11.8% at 24 hours from the treatment (P<0.001), 86.1% *vs.* 44.1% at 1 week (P<0.001), 91.7% *vs.* 70.6% at 1 month (P=0.032) and 94.4% *vs.* 79.4% at 3 months (P=0.078) respectively (Table II, Figure 1).[19] Patients from eSWL group required less auxiliary URS than patients from dSWL group (13.9% *vs.* 44.1%, P=0.039) and also the re-SWL treatment rates tended to be lower (8.3% *vs.* 32.4%, P=0.093) (Table II).

The early SFR (24 hours) was significantly higher in eSWL group even after stratification of the patients in subgroups with ≤10 mm (P=0.012), >10 mm (P=0.004) and distal stones (P=0.005). The statistical significance was not reach in the subgroup of patients with proximal stones (P=0.076); both a significant difference in mean stone size (11.2 *vs.* 13.8 mm, P=0.004) and an unequal distribution of patients with proximal stones (6 *vs.* 16) among the two groups was present. The 3 months SFR for patients from eSWL group with ≤10 mm stones was 100% both for proximal and distal. The 3 months retreatment rate was significantly higher in patients from dSWL group with ≤10 mm stones (61.1 *vs.* 15.8%, P=0.004) and distal stones (66.7 *vs.* 18.5%, P=0.001). The presence and the degree of hydronephrosis didn't have a significative impact on the results (Table III).

The efficiency quotient (EQ) was 52.8% *vs.* 11.8% at 24 hours and 79.1% *vs.* 57.5% at 3 months in eSWL and dSWL group respectively (P<0.01) (Figure 1).

Hospitalization was significantly lower in eSWL group (1.36 *vs.* 2.21 days, P=0.046) and no statistically significant differences were observed in complication rates (8.3 *vs.* 5.9%, P=0.691) among the two groups. One patient

COPYRIGHT© 2018 EDIZIONI MINERVA MEDICA

This document is protected by international copyright laws. No additional reproduction is authorized. It is permitted for personal use to download and save only one file and print only one copy of this Article. It is not permitted to make additional copies (either sporadically or systematically, either printed or electronic) of the Article for any purpose. It is not permitted to distribute the electronic copy of the article through online internet and/or intranet file sharing systems, electronic mailing or any other means which may allow access to the Article. The use of all or any part of the Article for any Commercial Use is not permitted. The creation of derivative works from the Article is not permitted. The production of reprints for personal or commercial use is not permitted. It is not permitted to remove, cover, overlay, obscure, block, or change any copyright notices or terms of use which the Publisher may post on the Article. It is not permitted to frame or use framing techniques to enclose any trademark, logo, or other proprietary information of the Publisher.

TABLE I.—*Patients' characteristics and relation to first SWL session.*

	Total	eSWL	dSWL	P value
Patients with ureteric colic (N.)	74	37	37	
Spontaneous passage	4	1	3	
Patients undergone SWL (N.)	70	36 (51.4%)	34 (48.6%)	
Gender, N. (%)				0.0004
Male	44 (62.9%)	18 (50%)	26 (76.5%)	
Female	26 (37.1%)	18 (50%)	8 (23.5%)	
Age (years)				
Mean	48.7	45.9	51.7	0.0954
95% CI lower-upper		41.2-50.7	46.7-56.7	
Median	49	45	53	
Range	24-81	24-76	25-81	
Stone location, N. (%)				
Proximal ureter	25 (35.7%)	9 (35%)	16 (47%)	0.0542
Distal ureter	45 (64.3%)	27 (65%)	18 (52.9%)	
Side, N. (%)				0.0976
Left	30 (42.9%)	12 (33.3%)	18 (52.9%)	
Right	40 (57.1%)	24 (66.6%)	16 (47.1%)	
Stone size (mm)				
Mean	9.83	9.03	10.68	0.0976
95% CI lower-upper		8.0-10.1	8.9-12.4	
Median	8.5	8.5	8.5	
Range	5-20	5-20	5-20	
Hydronephrosis, N. (%)				
No	2 (2.9%)	2 (5.6%)	0 (0%)	0.6567
Grade 1	40 (57.1%)	20 (55.6%)	20 (58.8%)	
Grade 2	28 (40%)	14 (38.9%)	14 (41.1%)	
Power (kV)				
Mean	19.21	19.83	18.56	0.0771
95% CI lower-upper		18.8-20.9	17.6-19.5	
Median	20	20	18	
Range	14-24	14-24	14-24	
Number of shocks (N.)				
Mean	2657	2678	2653	0.7720
95% CI lower-upper		2465-2889	2428-2842	
Median	3000	3000	3000	
Range	1000-3600	1000-3600	1400-3600	
Hospital stay (days)				
Mean	1.77	1.36	2.21	0.0461
Median	1	1	2	
Range	1-5	1-4	1-5	
Complications, N. (%)				
Mean	5 (7.1%)	3 (8.3%)	2 (5.9%)	NA

SWL: shock wave lithotripsy; eSWL: emergency shock wave lithotripsy group; dSWL: delayed shock wave lithotripsy group; 95% CI lower-upper: upper and lower limit of 95% confidence interval; (L/R): left/right side; kV: kilovolts; NA: not applicable.

TABLE II.—*SFR at 24 hours, 7 days, 1 and 3 months and need of auxiliary procedures in eSWL and dSWL group.*

SFR	eSWL		dSWL		OR	95% CI lower-upper	P
	N.	%	N.	%			
24 hours	19	52.8	4	11.8	0.119	0.035-0.409	0.0007
7 days	31	86.1	15	44.1	0.127	0.040-0.407	0.0005
1 month	33	91.7	24	70.6	0.218	0.054-0.879	0.0322
3 month	34	94.4	27	79.4	0.227	0.044-1.180	0.0782
Re-SWL	3	8.3	11	32.4	0.295	0.071-1.226	0.0931
Auxiliary-URS	5	13.9	15	44.1	0.181	0.057-0.579	0.0039

SFR: stone free rate (defined as no residual fragments visible on follow-up imaging); eSWL: emergency shock wave lithotripsy group; dSWL: delayed shock wave lithotripsy group; OR: odds ratio; 95% CI lower-upper: upper and lower limit of 95% confidence interval; re-SWL: repeated SWL; auxiliary URS: auxiliary ureteroscopy.

COPYRIGHT© 2018 EDIZIONI MINERVA MEDICA

This document is protected by international copyright laws. No additional reproduction is authorized. It is permitted for personal use to download and save only one file and print only one copy of this Article. It is not permitted to make additional copies (either sporadically or systematically, either printed or electronic) of the Article for any purpose. It is not permitted to distribute the electronic copy of the article through online internet and/or intranet file sharing systems, electronic mailing or any other means which may allow access to the Article. The use of all or any part of the Article for any Commercial Use is not permitted. The creation of derivative works from the Article is not permitted. The production of reprints for personal or commercial use is not permitted. It is not permitted to remove, cover, overlay, obscure, block, or change any copyright notices or terms of use which the Publisher may post on the Article. It is not permitted to frame or use framing techniques to enclose any trademark, logo, or other proprietary information of the Publisher.

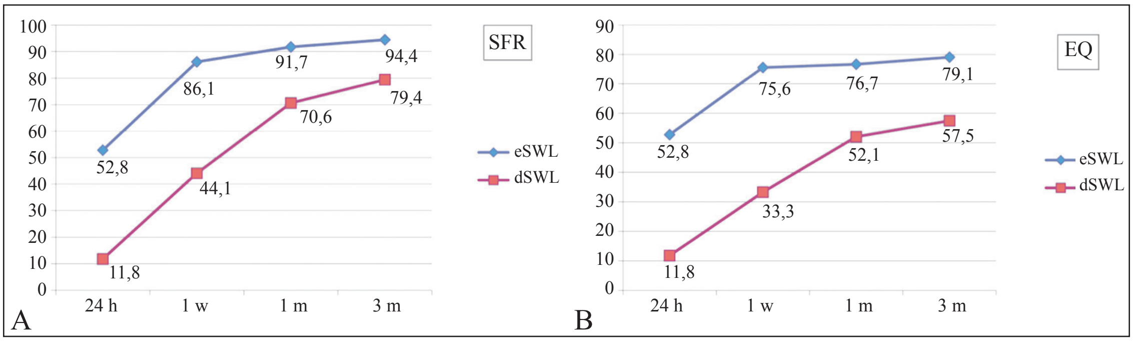

Figure 1.—A, B) SFR in patients from eSWL group and dSWL groupat 24 hours, 1 week, 1 and 3 months follow-up and EQ in patients from eSWL group and dSWL group at 24 hours, 1 week, 1 and 3 months follow-up.
SFR: stone free rates (defined as no residual fragments visible on follow-up imaging); EQ: efficienty quotient (according to the formula of Dentstedt *et al.*);[19] eSWL: emergency shock wave lithotripsy; dSWL: delayed shock wave lithotripsy.

Table III.—*Results as a function of stone location and size in eSWL and dSWL groups.*

Stone location and size	Mean size (mm)		P	SFR at 24 hours (%)		P	SFR at 3 months (%)		P	Retreatment rate at 3 month (%)		P	Hospitalization (days)		P
	eSWL	dSWL		eSWL	dSWL		eSWL	dSWL		eSWL	dSWL		eSWL	dSWL	
<10 mm	6.95	7.06	0.045	63.2%	22.2%	0.012	100%	88.9%	0.135	15.8%	27.8%	0.004	1.26	2.17	0.001
>10 mm	11.35	14.75	0.080	41.1%	0%	0.004	88.2%	68.8%	0.171	17.6%	25%	0.114	1.47	2.25	0.002
Proximal	11.22	13.81	0.044	33.3%	6.3%	0.076	88.9%	68.8%	0.258	22.2%	18.8%	0.282	1.56	2.13	0.006
Distal	8.29	7.89	0.070	59.3%	16.7%	0.005	96.3%	88.9%	0.329	14.8%	33.3%	0.001	1.29	2.28	0.001

SFR: stone free rate (defined as no residual fragments visible on follow-up imaging); eSWL: emergency shock wave lithotripsy group; dSWL: delayed shock wave lithotripsy group.

from the dSWL group developed pyelonephritis requiring a double J stent placement and antibiotics because of obstruction due to steinstrasse formation (Grade 3b). One patient from the eSWL group developed fever and 3 patients (2 from the eSWL group and 1 from the dSWL group) developed macroscopic hematuria (Grade 1) (Table I).

Discussion

Active stone removal is strongly indicated in patients with recurrent and persistent pain resistant to medical therapy, acute obstruction of the collecting system with impairment of the renal function, solitary kidney and urosepsis.[6] Currently the main debate gravitates around the choice of the therapeutic option (SWL or endourology procedure) and the choice of the correct setting (emercency or deferred).

The literature clearly shows that the spontaneous passage of ureteral stones depends on stone size and position. In a prospective study, Coll. *et al.* using helical CT, have demonstrated that the spontaneous passage rate for 5 to 7 mm stones is 60% decreasing to less than 25% for stones of 9 mm or larger. They have also showed that the spontaneous passage rate is 48% for proximal and 75% for distal stones.[19] Although observation of small stones is still recommended, most international guidelines indicate active removal of stones exceeding 5-7 mm not responding MET and analgesia.[12, 15] Therefore, in this study we included patients with >5 mm stones.

The rationale for early application of SWL is based mainly on the findings that the edema of the ureteral mucosa developes quickly (24-48 hours) and increase over time preventing an adequate delivery of the shockwave energy.[14] We decide to treat patients within 12 hours to minimize as much as possible both patient morbidity and the use of analgesics and to avoid ureteral edema formation that occurs gradually and fastly after the colic pain.

COPYRIGHT© 2018 EDIZIONI MINERVA MEDICA

This document is protected by international copyright laws. No additional reproduction is authorized. It is permitted for personal use to download and save only one file and print only one copy of this Article. It is not permitted to make additional copies (either sporadically or systematically, either printed or electronic) of the Article for any purpose. It is not permitted to distribute the electronic copy of the article through online internet and/or intranet file sharing systems, electronic mailing or any other means which may allow access to the Article. The use of all or any part of the Article for any Commercial Use is not permitted. The creation of derivative works from the Article is not permitted. The production of reprints for personal or commercial use is not permitted. It is not permitted to remove, cover, overlay, obscure, block, or change any copyright notices or terms of use which the Publisher may post on the Article. It is not permitted to frame or use framing techniques to enclose any trademark, logo, or other proprietary information of the Publisher.

We did not prescribe MET neither to patients from dSWL group waiting for the session nor to all patients after the treatment because its benefit to decrease the need for urological interventions has not been proven yet.[20] Porpiglia *et al.* conducted a prospective randomized study investigating the expulsion rate in nonresponder patients to a first cycle of MET (tamsulosin + deflazacort) underwent to a second 10-day cycle of MET with tamsulosin *vs.* placebo. The expulsion rate was significantly higher in the study group (80% *vs.* 49%, P<0.01), however MET did not reduce the expulsion time, number of colic episodes and analgesic use. Moreover the mean stone size was 6 mm and the study included only distal stones.[21]

SWL management of ureteral stones in emergency setting is completely lacking in the international guidelines. Some authors suggested the potential interest of SWL in emergency setting as treatment strategy for symptomatic ureteral stones.[10-13] It has been also reported that eSWL reduces repeated visits to emergency room, incidence of URS and hospitalization compared to delayed SWL.[22] In a meta-analysis of 570 patients, Picozzi *et al.* concluded that immediate SWL is a safe treatment with good success rate and less invasiveness than URS.[15, 23, 24]

Pace *et al.* demonstrated a superior success rate for upper and mid ureteral stones compared to distal calculi in a series of more that 1500 stones treated for the first time with extracorporeal lithotripsy.[25] Arrabal-Martin *et al.* reported comparable success rates for proximal stones treated with SWL and ureteroscopy.[26] In our study a non-stratified randomization method was used resulting in an imbalance between cohorts in mean stone size and location. In fact in the dSWL group proximal stones were significantly larger than distal (13.81 *vs.* 11.22 mm) and the mean stone size among patients with large stones (>10 mm) was also higher (14.75 *vs.* 11.35 mm). Regarding stone location, in the eSWL group the distribution of patients with proximal and distal stones wasn't equal (25 *vs.* 75%) compared to dSWL group where an equal distribution was present (47 *vs.* 53%). However, even after stratification per stone size and location, the early SFR (<24 hours) resulted significantly higher in the eSWL group in most of the cases (Table III).

In our series all the patients underwent SWL for ureteral stones in prone position with the shockwave source in contact with the anterior abdominal wall. Galli *et al.* reported a SFR of 85.7% and a re-treatment rate of 18.3% in a series of 70 patients underwent supine transgluteal SWL. Authors ascribed an easier focusing and a better real time visualization of the stone as the main advantages of this approach.[27]

In a small series of endoscopically controlled extracorporeal shockwave lithotripsy procedure, Traxer *et al.* showed that SWL could have a stone free rate up to 100% if the stone is well targeted and the excretory cavity are dilated.[28] Furthermore, in a recent comprehensive review of the literature, Kroczak *et al.* emphasized the importance of a proper patient selection and optimization of SWL technique to maximize the sucess rate of the procedure.[29] Also in our experience, we advocate that the ability of the operator in stone targeting and continuous monitoring are of primary importance for the success of the procedure.

In our series, the complication rates were in accordance with the literature with one major complication in dSWL group that solved with double J stenting, antibiotics and 5 days of hospitalization. One case of fever and 3 cases of macroscopic hematuria solved spontaneously. No cases of perinephric hematoma were observed.

Wherever lithotripter is available on a 24-hour basis, eSWL represents a non invasive way to perform stone fragmentation. Therefore its use is possible in early setting at the presentation of symptoms except in patients with absolute contraindications. The appropriate selection of patients is essential in order to avoid overtreatment of stones that would pass spontaneously. Due to our knowledge, prospective studies comparing eSWL and MET are lacking in the current literature. In fact would be interesting to compare these two options not only in terms of SFR but also in other outcomes as the use of analgesics and cost effectiveness. Patients who would benefit more from this treatment modality are those with a visible medium-size ureteral

COPYRIGHT© 2018 EDIZIONI MINERVA MEDICA

This document is protected by international copyright laws. No additional reproduction is authorized. It is permitted for personal use to download and save only one file and print only one copy of this Article. It is not permitted to make additional copies (either sporadically or systematically, either printed or electronic) of the Article for any purpose. It is not permitted to distribute the electronic copy of the article through online internet and/or intranet file sharing systems, electronic mailing or any other means which may allow access to the Article. The use of all or any part of the Article for any Commercial Use is not permitted. The creation of derivative works from the Article is not permitted. The production of reprints for personal or commercial use is not permitted. It is not permitted to remove, cover, overlay, obscure, block, or change any copyright notices or terms of use which the Publisher may post on the Article. It is not permitted to frame or use framing techniques to enclose any trademark, logo, or other proprietary information of the Publisher.

stone (7-10 mm) and non-responders to analgesia who needs a fast recovery. Indeed patient counseling and provision of treatment information is mandatory.

Limitations of the study

The main limitations of our study are the small number of patients and the heterogeneity of the study population with particular regards to stone size and location. Moreover not all the patients underwent CT scan examination during the diagnostic evaluation therefore was not possible to include the stone composition (Hounsfield Unit) and the skin-to-stone distance data in the statistical analysis.

Conclusions

Emergency SWL after a first onset of colic pain represents an effective treatment. It delivers high SFR even within 24 hours from the treatment especially in ≤10 mm stones. This procedure is safe and is able to reduce the number of auxiliary procedures (URS or re-SWL) and the hospitalization. We believe that "emergency" timing of the precedure is remarkably important because of quick development ureteral edema.

References

1. Trinchieri A. Epidemiology of urolithiasis. Arch Ital Urol Androl 1996;68:203–49.

2. Hollingsworth JM, Rogers MA, Kaufman SR, Bradford TJ, Saint S, Wei JT, *et al.* Medical therapy to facilitate urinary stone passage: a meta-analysis. Lancet 2006;368:1171–9.

3. Liu M, Henderson SO. Myth: nephrolithiasis and medical expulsive therapy. CJEM 2007;9:463–5.

4. Mozafarpour S, Hernandez N, Lipkin M, Eisner BH. Outcomes of ureteroscopy. Minerva Urol Nefrol 2016;68:560–9.

5. Teichman JM. Clinical practice. Acute renal colic from ureteral calculus. N Engl J Med 2004;350:684–93.

6. Türk C, Petřík A, Sarica K, Seitz C, Skolarikos A, Straub M, *et al.* EAU Guidelines on Diagnosis and Conservative Management of Urolithiasis. Eur Urol 2016;69:468–74.

7. Pickard R, Starr K, MacLennan G, Lam T, Thomas R, Burr J, *et al.* Medical expulsive therapy in adults with ureteric colic: a multicentre, randomised, placebo-controlled trial. Lancet 2015;386:341–9.

8. Preminger GM, Tiselius HG, Assimos DG, Alken P, Buck C, Gallucci M, *et al.*; EAU/AUA Nephrolithiasis Guideline Panel. 2007 guideline for the management of ureteral calculi. J Urol 2007;178:2418–34.

9. Seitz C, Tanovic E, Kikic Z, Memarsadeghi M, Fajkovic H. Rapid extracorporeal shock wave lithotripsy for proximal ureteral calculi in colic versus noncolic patients. Eur Urol 2007;52:1223–7.

10. Tligui M, El Khadime MR, Tchala K, Haab F, Traxer O, Gattegno B, *et al.* Emergency extracorporeal shock wave lithotripsy (ESWL) for obstructing ureteral stones. Eur Urol 2003;43:552–5.

11. Ghalayini IF, Al-Ghazo MA, Khader YS. Evaluation of emergency extracorporeal shock wave lithotripsy for obstructing ureteral stones. Int Braz J Urol 2008;34:433–40.

12. Tombal B, Mawlawi H, Feyaerts A, Wese FX, Opsomer R, Van Cangh PJ. Prospective randomized evaluation of emergency extracorporeal shock wave lithotripsy (ESWL) on the short-time outcome of symptomatic ureteral stones. Eur Urol 2005;47:855–9.

13. Kravchick S, Bunkin I, Stepnov E, Peled R, Agulansky L, Cytron S. Emergency extracorporeal shockwave lithotripsy for acute renal colic caused by upper urinary-tract stones. J Endourol 2005;19:1–4.

14. Deliveliotis C, Chrisofos M, Albanis S, Serafetinides E, Varkarakis J, Protogerou V. Management and follow-up of impacted ureteral stones. Urol Int 2003;70:269–72.

15. Picozzi SC, Ricci C, Gaeta M, Casellato S, Stubinski R, Ratti D, *et al.* Urgent shock wave lithotripsy as first-line treatment for ureteral stones: a meta-analysis of 570 patients. Urol Res 2012;40:725–31.

16. Panah A, Patel S, Bourdoumis A, Kachrilas S, Buchholz N, Masood J. Factors predicting success of emergency extracorporeal shockwave lithotripsy (eESWL) in ureteric calculi—a single centre experience from the United Kingdom (UK). Urolithiasis 2013;41:437–41.

17. Kumar A, Mohanty NK, Jain M, Prakash S, Arora RP. A prospective randomized comparison between early (<48 hours of onset of colicky pain) *versus* delayed shockwave lithotripsy for symptomatic upper ureteral calculi: a single center experience. J Endourol 2010;24:2059–66.

18. Joshi HB, Obadeyi OO, Rao PN. A comparative analysis of nephrostomy, JJ stent and urgent in situ extracorporeal shock wave lithotripsy for obstructing ureteric stones. BJU Int 1999;84:264–9.

19. Coll DM, Varanelli MJ, Smith RC. Relationship of spontaneous passage of ureteral calculi to stone size and location as revealed by unenhanced helical CT. AJR Am J Roentgenol 2002;178:101–3.

20. Loftus C, Nyame Y, Hinck B, Greene D, Chaparala H, Alazem K, *et al.* Medical Expulsive Therapy is Underused for the Management of Renal Colic in the Emergency Setting. J Urol 2016;195:987–91.

21. Porpiglia F, Fiori C, Ghignone G, Vaccino D, Billia M, Morra I, *et al.* A second cycle of tamsulosin in patients with distal ureteric stones: a prospective randomized trial. BJU Int 2009;103:1700–3.

22. Argyropoulos AN, Tolley DA. Optimizing shock wave lithotripsy in the 21st century. Eur Urol 2007;52:344–52.

23. Fiori C. Life-threatening complications: "the dark side" of ureteroscopy. Minerva Urol Nefrol 2017;69:521.

24. Volkin D, Shah O. Complications of ureteroscopy for stone disease. Minerva Urol Nefrol 2016;68:570–85.

25. Pace KT, Weir MJ, Tariq N, Honey RJ. Low success rate of repeat shock wave lithotripsy for ureteral stones after failed initial treatment. J Urol 2000;164:1905–7.

26. Arrabal-Martín M, Pareja-Vilches M, Gutiérrez-Tejero F, Miján-Ortiz JL, Palao-Yago F, Zuluaga-Gómez A. Therapeutic options in lithiasis of the lumbar ureter. Eur Urol 2003;43:556–63.

COPYRIGHT© 2018 EDIZIONI MINERVA MEDICA

This document is protected by international copyright laws. No additional reproduction is authorized. It is permitted for personal use to download and save only one file and print only one copy of this Article. It is not permitted to make additional copies (either sporadically or systematically, either printed or electronic) of the Article for any purpose. It is not permitted to distribute the electronic copy of the article through online internet and/or intranet file sharing systems, electronic mailing or any other means which may allow access to the Article. The use of all or any part of the Article for any Commercial Use is not permitted. The creation of derivative works from the Article is not permitted. The production of reprints for personal or commercial use is not permitted. It is not permitted to remove, cover, overlay, obscure, block, or change any copyright notices or terms of use which the Publisher may post on the Article. It is not permitted to frame or use framing techniques to enclose any trademark, logo, or other proprietary information of the Publisher.

27. Galli R, Sighinolfi MC, Micali S, Martorana E, Rosa M, Mofferdin A, *et al*. Advantages of the supine transgluteal approach for distal ureteral stone extracorporeal shock wave lithotripsy: outcomes based on CT characteristics. Minerva Urol Nefrol 2017;69:189–94.

28. Traxer O, Letendre J. Extracorporeal lithotripsy endoscopically controlled by ureterorenoscopy (LECURS): a new concept for the treatment of kidney stones-first clinical experience using digital ureterorenoscopes. World J Urol 2014;32:715–21.

29. Kroczak T, Scotland KB, Chew B, Pace KT. Shockwave lithotripsy: techniques for improving outcomes. World J Urol 2017;35:1341–6.

Conflicts of interest.—The authors certify that there is no conflict of interest with any financial organization regarding the material discussed in the manuscript.

Authors' contributions.—Bucci Stefano protocol development; Umari Paolo: data collecting and manuscript writing and editing; Rizzo Michele: data collecting; Nicola Pavan: data collecting; Liguori Giovanni: manuscript writing and editing; Barbone Fabio: data analysis; Trombetta Carlo: protocol development, manuscript writing and editing.

Article first published online: May 14, 2018. - Manuscript accepted: May 11, 2018. - Manuscript revised: April 18, 2018. - Manuscript received: November 2, 2017.
