# Supplementary material for: Efficacy of emergency extracorporeal shock wave lithotripsy in the treatment of ureteral stones: a meta-analysis
Source: BMC Urol. 2023 Apr 4;23:56. doi: 10.1186/s12894-023-01226-5 (PMC10074806; doi:10.1186/s12894-023-01226-5)
Supplement: Supplementary file 2 — Additional File 2: Choi 2012 [file 12894_2023_1226_MOESM2_ESM.pdf]

KJU Korean Journal of Urology

www.kjurology.org
http://dx.doi.org/10.4111/kju.2012.53.12.853

Urolithiasis

Usefulness of Early Extracorporeal Shock Wave Lithotripsy in Colic Patients with Ureteral Stones

Hyeung Joon Choi, Jin-Hee Jung[1], Jungbum Bae, Min Chul Cho, Hae Won Lee, Kwang Soo Lee

Departments of Urology and [1]Radiology, Dongguk University Ilsan Hospital, Goyang, Korea

Purpose: To compare efficacy and safety between early extracorporeal shock wave lithotripsy (eESWL) and deferred ESWL (dESWL) in colic patients with ureteral stones and to investigate whether eESWL can play a critical role in improving treatment outcomes.
Materials and Methods: A total of 279 patients who underwent ESWL for single radio-opaque ureteral stones of 5 to 20 mm in size were included in this retrospective study. The patients were categorized into two groups according to the time between the onset of colic and ESWL: eESWL ($<$48 hours, n=153) and dESWL ($\geq$48 hours, n=126). Success was defined as stone-free status as shown on a plain radiograph within 1 month of the first session.
Results: For all patients, the success rate in the eESWL group was significantly higher than that in the dESWL group. The eESWL group required significantly fewer ESWL sessions and less time to achieve stone-free status than did the dESWL group. For 241 patients with stones $<$10 mm, all treatment outcomes in the former group were superior to those in the latter group, but not for 38 patients with stones sized 10 to 20 mm. The superiority of eESWL over dESWL in the treatment outcomes was more pronounced for proximal ureteral stones than for mid-to-distal ureteral stones. Post-ESWL complication rates were comparable between the two groups. In the multivariate analysis, smaller stone size and a time to ESWL of $<$48 hours were independent predictors of success.
Conclusions: Our data suggest that eESWL in colic patients with ureteral stones is an effective and safe treatment with accelerated stone clearance.

Key Words: *Colic; Lithotripsy; Ureteral calculi*

This is an Open Access article distributed under the terms of the Creative Commons Attribution Non-Commercial License (http://creativecommons.org/licenses/by-nc/3.0) which permits unrestricted non-commercial use, distribution, and reproduction in any medium, provided the original work is properly cited.

Article History:
received 19 August, 2012
accepted 24 September, 2012

Corresponding Author:
Kwang Soo Lee
Department of Urology, Dongguk University Ilsan Hospital, 27 Dongguk-ro, Ilsandong-gu, Goyang 410-773, Korea
TEL: +82-31-961-7450
FAX: +82-31-961-7457
E-mail: urolee51@yahoo.co.kr

INTRODUCTION

Colicky pain (colic) due to ureteral stones is a widespread problem in the field of urology. Although various treatment options are available, such as expectant management, extracorporeal shock wave lithotripsy (ESWL), and ureteroscopic stone removal, conservative management is usually performed with deferral of definite treatment for patients with acute colic. However, because the probability of spontaneous passage of ureteral stones depends on stone size and, to a lesser extent, on stone location at presentation, the likelihood of spontaneous passage decreases rapidly for stones $\geq$5 mm [1,2]. Furthermore, the conservative approach is often complicated by recurrent pain, frequent visits to the emergency room, absence from work, and an increased risk of complications including pyelonephritis and silent loss of renal function [3].

Despite the increased use of alpha-blockers and recent improvements in ureteroscopic technology, ESWL remains an attractive treatment option for clearance of ureteral stones owing to this benefits of this noninvasive intervention, such as no requirement for anesthesia and low morbidity [4]. Furthermore, it is one of the suitable treatment options for stones in all locations within the ureter, as suggested by the recent joint American Urological Association/European Association of Urology guideline

[1]. However, stone-free status by ESWL is not immediately achieved, and it can require a variable period of time for ureteral stones to be eliminated completely, depending on several factors, including stone size, location, degree of stone impaction, and edema of the ureteral mucosa. This concern about the development or progression of edema of the ureteral mucosa has raised the possibility of rapid application of ESWL for colic due to ureteral stones. Thus, the rationale for the application of early ESWL (eESWL) for colic due to ureteral stones is to achieve maximum stone clearance before the surrounding edema develops or progresses. Recently, several studies have demonstrated the benefit of eESWL for colic patients with ureteral stones [2,3,5-8]. However, conclusions on ESWL treatment of patients in an acute setting are lacking in the international guidelines [9]. Also, the data still appear to be limited on the effectiveness of eESWL compared with the well-documented advances in ureteroscopy [4].

The aim of this study was to compare efficacy and safety between eESWL (within 48 hours after the onset of colic) and deferred ESWL (dESWL, 48 hours or later after the onset of colic) for colic patients with ureteral stones and to investigate whether eESWL can play a critical role in improving treatment outcomes.

MATERIALS AND METHODS

1. Study design

This retrospective study was approved by the Institutional Review Board at Dongguk University Ilsan Hospital. Between July 2005 and December 2011, a total of 567 patients visited the emergency room of our hospital or the outpatient clinic of our department owing to colic caused by radio-opaque, solitary ureteral stones. Among these, 324 patients underwent ESWL. After excluding 5 patients who underwent placement of a ureteral stent or percutaneous nephrostomy tube before ESWL and 40 patients who were lost to follow-up after ESWL or with missing data, a total

TABLE 1. Baseline patient or stone characteristics and ESWL treatment data

Variable	eESWL (n=153)	dESWL (n=126)	p-value
Demographics & clinical parameters			
Age (yr)	43.6±12.9	45.1±13.1	0.458
Gender (male/female)	108/45	96/30	0.411
Body mass index (kg/m^2)	25.3±3.6	25.0±3.3	0.576
Serum creatinine (mg/dl)	0.9±0.2	1.0±0.3	0.057
MDRD GFR (ml/min/1.73 m^2)	92.5±20.5	86.8±22.5	0.087
Radiologic parameters			
Stone size (mm)	7.6±2.5	8.3±3.1	0.105
Stone location (proximal/mid/distal)	93/6/54	90/6/30	0.211
Stone laterality (right/left)	72/81	63/63	0.767
Average Hounsfield unit	792.9±358.7	854.7±360.6	0.274
Presence of hydronephrosis	141 (92.2)	101 (95.2)	0.772
Presence of tissue rim sign	105 (68.6)	93 (73.8)	0.483
Skin-to-stone distance (cm)	11.1±1.3	11.1±1.7	0.692
ESWL treatment data			
Time to ESWL (h)	11.6±10.0	161.9±140.9	<0.001
No. of ESWL sessions performed	1.7±1.1	2.1±1.3	0.017
Total no. of shocks per stone delivered	5,061±3,365	6,366±3,927	0.017

Values are presented as mean±SD or no. of patients (%).
ESWL, extracorporeal shock wave lithotripsy; eESWL, early ESWL; dESWL, deferred ESWL; MDRD GFR, modification of diet in renal disease glomerular filtration rate (calculated using the abbreviated MDRD study equation).

TABLE 2. Comparison of post-ESWL complications between the eESWL and dESWL groups

	eESWL	dESWL	CDC	Management
Steinstrasse	3 (2.0)	2 (1.6)		
Asymptomatic	2 (1.3)	1 (0.8)	I	Observation and spontaneous passage
Symptomatic	1 (0.7)	1 (0.8)	IIIa	Ureteroscopic removal
Gross hematuria	2 (1.3)	1 (0.8)	I	Conservative
Severe pain	6 (3.9)	5 (4.0)	I	Medication with supplementary analgesics
Total	14 (9.2)	10 (7.9)		

Values are presented as no. of patients (%).
ESWL, extracorporeal shock wave lithotripsy; eESWL, early ESWL; dESWL, deferred ESWL; CDC, Clavien-Dindo classification.

TABLE 3. Comparison of treatment outcomes between eESWL and dESWL groups according to stone size or location

	eESWL	dESWL	p-value
Total	(n=153)	(n=126)	
No. of treatment success	135 (88.2)	84 (66.7)	<0.001
No. of ESWL sessions performed	1.7±1.1	2.1±1.3	0.011
Time to stone-free (d)	18.4±31.7	31.7±47.2	0.011
Stone size			
<10 mm	(n=139)	(n=102)	
No. of treatment success	127 (91.4)	70 (68.6)	<0.001
No. of ESWL sessions performed	1.6±0.9	1.9±1.1	0.019
Time to stone-free (d)	15.8±22.6	30.2±50.3	0.023
≥10 mm	(n=13)	(n=25)	
No. of treatment success	8 (61.5)	14 (56.0)	0.513
No. of ESWL sessions performed	2.9±1.7	3.1±2.0	0.500
Time to stone-free (d)	37.8±32.1	47.5±61.4	0.409
Stone location			
Proximal ureter	(n=93)	(n=93)	
No. of treatment success	81 (87.1)	57 (61.3)	<0.001
No. of ESWL sessions performed	1.8±1.2	2.2±1.3	0.047
Time to stone-free (d)	21.4±34.9	35.3±53.5	0.049
Mid-to-distal ureter	(n=59)	(n=34)	
No. of treatment success	54 (91.5)	26 (76.5)	0.057
No. of ESWL sessions performed	1.5±0.9	1.9±1.2	0.074
Time to stone-free (d)	13.4±11.7	21.2±19.4	0.038

Values are presented as mean±SD or no. of patients (%).
ESWL, extracorporeal shock wave lithotripsy; eESWL, early ESWL; dESWL, deferred ESWL.

TABLE 4. Predictors of ESWL success in all patients (univariate and multivariate logistic regression analysis)

Variable	OR (95% CI)	p-value	Adjusted OR (95% CI)	p-value
Age	0.983 (0.958–1.009)	0.210	1.002 (0.957–1.050)	0.916
Body mass index	0.965 (0.856– 1.088)	0.557	1.119 (0.940–1.333)	0.206
Sex		0.432		0.902
Male	1.000		1.000	
Female	1.391 (0.610–3.171)		1.094 (0.261–4.587)	
MDRD GFR	1.002 (0.985–1.020)	0.809	0.991 (0.966–1.017)	0.509
Stone size	0.805 (0.715–0.907)	<0.001	0.808 (0.699–0.934)	0.004
Stone laterality		0.610		0.546
Right	1.000		1.000	
Left	1.200 (0.596–2.417)		1.397 (0.472–4.129)	
Stone location		0.056		0.582
Proximal ureter	1.000		1.000	
Mid-to-distal ureter	2.280 (0.979–5.307)		1.454 (0.384–5.502)	
Average HU	0.999 (0.998–1.000)	0.024	0.999 (0.998–1.001)	0.547
Hydronephrosis		0.800		0.648
Absence	1.000		1.000	
Presence	1.190 (0.309–4.587)		1.549 (0.237–10.126)	
Tissue rim sign		0.079		0.213
Absence	1.000		1.000	
Presence	0.401 (0.145–1.110)		0.422 (0.109–1.640)	
Skin-to-stone distance	0.998 (0.973–1.203)	0.860	0.986 (0.946–1.028)	0.509
Time to ESWL (h)		<0.001		0.022
≥48	1.000		1.000	
<48	4.165 (1.921–9.031)		3.811 (1.212–11.987)	

ESWL, extracorporeal shock wave lithotripsy; OR, odds ratio; CI, confidence interval; MDRD GFR, modification of diet in renal disease glomerular filtration rate (calculated using the abbreviated MDRD study equation); HU, Hounsfield unit.

of 279 patients with stones sized 5 to 20 mm were included in this study. Other exclusion criteria were as follows: stones >20 mm in largest diameter, absolute contraindication to ESWL such as pregnancy or coagulopathy, previous history of ureteral surgery, structural urinary tract abnormality, and a solitary kidney. No patient was excluded because of these conditions. To investigate the prognostic significance of eESWL for ureteral stones, all eligible patients were categorized into two groups according to the time between the onset of colic and ESWL treatment as follows: the eESWL group (<48 hours, n=153) and the dESWL group (≥48 hours, n=126). All patients underwent a baseline evaluation with medical history, physical examination, routine blood or urine tests, plain radiography of kidney-ureter-bladder (KUB), and noncontrast computed tomography (NCCT) with three-dimensional reconstruction. On the basis of the radiologic findings, stone factors including stone size, location, presence or absence of hydronephrosis, tissue rim sign, skin-to-stone distance (SSD), and average Hounsfield unit (stone density) were reviewed by a single radiologist who was blinded to the ESWL outcomes. Also, demographic variables, laboratory findings, and data on ESWL treatment or auxiliary procedures or post-ESWL complications were compared between the two groups. The stone size was defined as the greatest diameter in any dimension. The tissue rim sign is recognized as a circumferential area of soft-tissue attenuation surrounding a suspended ureteral stone [10]. The average SSD was calculated by measuring three distances from the center of the stone to the skin (0°, 45°, and 90°) by use of radiographic callipers as described previously [11]. Stone-free status was assessed by use of follow-up KUB performed at 1 week after each ESWL session and was defined as no radiologic evidence of stones on KUB. In cases for which residual fragments could not be excluded, NCCT was performed. Repeated ESWL sessions were performed immediately when follow-up KUB showed inadequate fragmentation of the stone. Success was defined as stone-free status within 1 month after the first ESWL session. All other outcomes were regarded as failures. Post-ESWL complications were graded by using the Clavien-Dindo classification [12].

2. ESWL procedure

All patients received analgesia in the form of an intramuscular injection of ketorolac (30 mg) or an intravenous injection of pethidine (50 mg). In all cases, ESWL procedures were performed by using the same electromagnetic Dornier Compact Delta lithotriptor (Dornier Medtech, Kennesaw, GA, USA). A maximum of 3,000 shocks per session were delivered to each stone at a frequency of 100 shocks per minute and the power setting of 15 to 20 kV under fluoroscopy.

3. Statistical analysis

Statistical analysis was performed by using the Fisher's exact test or chi-square test for categorical data and the Student's t-test for continuous variables. To identify the independent predictors of success, we used backward stepwise logistic regression analysis. A 5% level of significance was used for all statistical testing, and all statistical tests were two-sided. The SPSS ver. 17.0 (SPSS Inc., Chicago, IL, USA) was used for the analyses.

RESULTS

The baseline characteristics of the patients and the ESWL treatment data are shown in Table 1. There were no significant differences between the two groups in baseline parameters including stone size and location. The eESWL group required significantly fewer ESWL sessions than did the dESWL group. For the eESWL group, 90 (58.8%), 36 (23.5%), 6 (3.9%), and 6 patients (3.9%) required 1, 2, 3, and ≥4 sessions of ESWL for stone-free status, respectively. The remaining 15 cases who did not respond to ESWL underwent ureteroscopic stone removal. For the dESWL group, 48 (38.1%), 27 (21.4%), 6 (4.8%), and 12 patients (9.5%) required 1, 2, 3, and ≥4 sessions of ESWL for stone-free status, respectively. The remaining 33 patients underwent ureteroscopic stone removal as an auxiliary procedure. Post-ESWL complication rates were compara-

TABLE 5. Predictors of ESWL success in 241 patients with ureteral stones of <10 mm (multivariate logistic regression analysis)

Variable	Adjusted OR (95% CI)	p-value
Age	1.005 (0.948–1.066)	0.861
Body mass index	1.089 (0.896–1.324)	0.391
Sex		0.624
Male	1.000	
Female	0.676 (0.141–3.236)	
MDRD GFR	0.994 (0.964–1.024)	0.687
Stone size	0.505 (0.319–0.801)	0.004
Stone laterality		0.849
Right	1.000	
Left	1.138 (0.303–4.275)	
Stone location		0.725
Proximal ureter	1.000	
Mid-to-distal ureter	1.333 (0.269–6.616)	
Average HU	0.999 (0.997–1.002)	0.609
Hydronephrosis		0.591
Absence	1.000	
Presence	1.976 (0.165–23.667)	
Tissue rim sign		0.368
Absence	1.000	
Presence	0.500 (0.111–2.259)	
Skin-to-stone distance	0.984 (0.936–1.034)	0.520
Time to ESWL (h)		0.007
≥48	1.000	
<48	8.179 (1.792–37.345)	

ESWL, extracorporeal shock wave lithotripsy; OR, odds ratio; CI, confidence interval; MDRD GFR, modification of diet in renal disease glomerular filtration rate (calculated using the abbreviated MDRD study equation); HU, Hounsfield unit.

ble between the two groups (Table 2).

The comparison of treatment outcomes (success rate, time to stone-free status, and number of ESWL sessions performed) between the eESWL and dESWL groups according to stone size or location is shown in Table 3. In the eESWL group, 135 patients (88.2%) in whom the stones were completely eliminated within 1 month after the first ESWL session were considered to have a successful outcome. In the dESWL group, 84 patients (66.7%) were classified as having a successful outcome. Mean time to stone-free status after the first ESWL session was significantly shorter in the eESWL group than in the dESWL group. For the subset of 241 patients with stone size <10 mm, all treatment outcomes in the eESWL group were significantly superior to those in the dESWL group. On the other hand, in 38 patients with stone size ≥10 mm, there was no significant difference in any treatment outcome between the two groups. In the subset of 186 patients with proximal ureteral stones, all treatment outcomes in the eESWL group were significantly better than those in the dESWL group. For 93 patients with mid-to-distal ureteral stones, the eESWL group had a tendency toward a greater success rate and a fewer number of ESWL sessions compared with the dESWL group, but this difference was not statistically significant. In terms of time to stone-free status, stone clearance was significantly accelerated in the former group compared with the latter group.

In a multivariate analysis, smaller stone size and a time to ESWL of <48 hours were independent predictors of success, and patients who underwent eESWL had a 3.8 times higher probability of ESWL success than did those who underwent dESWL (Table 4). In the subset of 241 patients with a stone size <10 mm, the multivariate analysis revealed that stone size and the time to ESWL were independent predictors of ESWL outcome (Table 5). However, the multivariate analysis was not performed for 38 patients with a stone size ≥10 mm because the sample size was too small for accurate analysis. In the subset of patients with proximal ureteral stones, stone size and the time to ESWL were independent predictors of ESWL success (Table 6). In the patients with mid-to-distal ureteral stones, only the average SSD was independently associated with ESWL success (Table 6).

DISCUSSION

The relatively high efficacy and low morbidity of ESWL can justify its application in the treatment of ureteral stones accompanied by colic, often in response to patients' demand [8]. Nevertheless, its role as a first-line therapeutic option applied in the early stage after the onset of colic has received limited attention, and the role of eESWL in symptomatic ureteral stones has not yet been established [5]. Thus, the present study can extend the current state of knowledge in the study of the role of eESWL in colic patients with ureteral stones by further exploring ESWL treatment outcomes and identifying the independent predictors of successful outcome for each subset stratified ac-

TABLE 6. Predictors of ESWL success according to stone location (multivariate logistic regression analysis)

Variable	Proximal ureter		Mid-to-distal ureter	
	Adjusted OR (95% CI)	p-value	Adjusted OR (95% CI)	p-value
Age	0.982 (0.935–1.031)	0.457	0.984 (0.922–1.050)	0.620
Body mass index	1.196 (0.947–1.511)	0.134	1.222 (0.832–1.612)	0.366
Sex		0.970		0.101
Male	1.000		1.000	
Female	0.969 (0.185–5.077)		1.919 (0.625–3.697)	
MDRD GFR	0.991 (0.958–1.026)	0.619	0.993 (0.945–1.045)	0.798
Stone size	0.825 (0.708–0.962)	0.014	0.734 (0.382–1.410)	0.354
Stone laterality		0.122		0.053
Right	1.000		1.000	
Left	2.827 (0.757–10.566)		0.053 (0.003–1.036)	
Average HU	1.000 (0.998–1.002)	0.814	0.997 (0.994–1.000)	0.083
Hydronephrosis		0.944		0.543
Absence	1.000		1.000	
Presence	0.931 (0.128–6.780)		0.842 (0.232–1.452)	
Tissue rim sign		0.531		0.513
Absence	1.000		1.000	
Presence	2.068 (0.213–20.133)		0.363 (0.017–7.596)	
Skin-to-stone distance	0.985 (0.949–1.022)	0.409	0.901 (0.814–0.996)	0.042
Time to ESWL (h)		0.012		0.077
≥48	1.000		1.000	
<48	4.388 (1.646–13.034)		7.262 (0.807–65.353)	

ESWL, extracorporeal shock wave lithotripsy; OR, odds ratio; CI, confidence interval; MDRD GFR, modification of diet in renal disease glomerular filtration rate (calculated using the abbreviated MDRD study equation); HU, Hounsfield unit.

cording to stone size or location. The major findings of this study can be briefly summarized as follows: 1) eESWL in colic patients with ureteral stones can achieve a higher success rate with more accelerated elimination of ureteral stones and a fewer number of ESWL sessions than for dESWL, and 2) the benefit of eESWL over dESWL was more pronounced in patients with stones <10 mm or located in the proximal ureter.

The rationale for earlier application of ESWL in patients with ureteral stones accompanied by colic is based mainly on the finding that edema of the ureteral mucosa begins developing after 24 to 48 hours and progresses over time, leading to impaired elimination of stones after ESWL [9,13,14]. Thus, edema of the ureteral mucosa is associated with the development of stone impaction, which is supported by a previous study demonstrating that the mucosa in the stone bed shows morphological changes that develop after 48 hours, such as a hyperplastic appearance and increased mitotic activity on histological examination [13,14]. This gradual increase in edema of the ureteral mucosa can prevent the formation of an expansion chamber and liquid interface, which can impede fragmentation by ESWL [9,13,14]. Also, Cummings et al. [15] showed that the duration of symptoms before presentation was the most important factor in predicting the passage of ureteral stones by use of an artificial neural network model. On the basis of the above studies, in the present study, eESWL was defined as ESWL performed within 48 hours after the onset of colic. Our study demonstrated that patients who underwent eESWL had a significantly higher probability of treatment success with more accelerated elimination of stones and a fewer number of ESWL sessions than did those who underwent dESWL. Taken together, these data suggest that it seems reasonable for colic patients with ureteral stones to undergo ESWL at an earlier stage after the onset of colic before morphological changes such as edema of the ureteral mucosa occur.

Another major finding of this study was that the superiority of eESWL over dESWL was more pronounced for proximal ureteral stones than for mid-to-distal ureteral stones. Similarly, previous studies have shown that the beneficial effect of eESWL was more pronounced in patients with proximal ureteral stones than in those with mid-to-distal ureteral stones [3,7]. Furthermore, previous studies of colic patients with proximal ureteral stones have shown that eESWL can offer better treatment outcomes compared with dESWL [2,5,6,16]. Thus, the data suggest that eESWL can achieve maximal stone clearance in the shortest possible time, particularly in patients with proximal ureteral stones. On the other hand, in patients with mid-to-distal ureteral stones, we only observed a trend toward a higher success rate and fewer number of sessions in patients treated by eESWL compared with dESWL. This lack of statistical significance might be partly due to the relatively small sample size of the subset. In terms of time to stone-free status after the first session of ESWL, elimination of stones was significantly more accelerated in the eESWL group than in the dESWL group. Accordingly, the eESWL treatment may be also helpful, to a certain degree, in colic patients with mid-to-distal ureteral stones.

The stone-free rate after ESWL is known to be negatively affected by increasing size of ureteral stones [1]. To date, the data for an impact of stone size on the stone-free rate after eESWL are inconclusive. Ghalayini et al. [3] demonstrated that success rates after eESWL were negatively correlated with stone size. Also, Tligui et al. [8] showed the highest success rate in patients with 6- to 10-mm sized stones and the worst outcome in those with 10- to 20-mm sized stones. Similarly, our study showed that the therapeutic gain of eESWL over dESWL was more pronounced in patients with ureteral stones <10 mm, whereas the treatment outcomes were not significantly different between the two in patients with 10- to 20-mm sized stones. On the other hand, Tombal et al. [7] noted that the therapeutic benefit by eESWL was modest for ureteral stones ≤5 mm but evident for stones >5 mm. However, because the study by Tombal et al. [7] did not include patients with ureteral stones >10 mm, it may be difficult to directly compare ESWL outcomes according to stone size among the above studies.

In our study, the success rates in the eESWL and dESWL groups were 88.2% and 66.7%, respectively. This success rate after eESWL was a little higher than that in the study by Kravchick et al. [16], whereas those after dESWL in the two studies were similar [15]. On the other hand, the success rate after dESWL in this study was a little lower than in the recent studies [2,5]. This may be a result of the heterogeneity of the patients, differences in follow-up or definition of success, types of lithotripter, focusing mechanism, power setting, or different distribution of stone composition [9].

Some limitations of our study warrant consideration. First, our study was limited by its retrospective nature. Second, the routine radiological follow-up of this study was performed by using KUB but not CT. Thus, it might be difficult to exactly assess stone-free status. Last, we did not investigate stone composition because a considerable number of the passed stones were not collected by the patients.

CONCLUSIONS

Our data suggest that eESWL in patients with colic by ureteral stones is an effective and safe treatment that results in accelerated elimination of ureteral stones. Thus, it can be beneficial for colic patients with ureteral stones to undergo ESWL treatment as soon as possible. Also, our results indicate that eESWL is particularly favorable for stones <10 mm or located in the proximal ureter. Larger, well-organized, randomized trials are needed to confirm these findings.

CONFLICTS OF INTEREST

The authors have nothing to disclose.

REFERENCES

1. Preminger GM, Tiselius HG, Assimos DG, Alken P, Buck C, Gallucci M, et al. 2007 guideline for the management of ureteral calculi. J Urol 2007;178:2418-34.
2. Seitz C, Fajkovic H, Remzi M, Waldert M, Ozsoy M, Kramer G, et al. Rapid extracorporeal shock wave lithotripsy treatment after a first colic episode correlates with accelerated ureteral stone clearance. Eur Urol 2006;49:1099-105.
3. Ghalayini IF, Al-Ghazo MA, Khader YS. Evaluation of emergency extracorporeal shock wave lithotripsy for obstructing ureteral stones. Int Braz J Urol 2008;34:433-40.
4. Dasgupta R, Hegarty N, Thomas K. Emergency shock wave lithotripsy for ureteric stones. Curr Opin Urol 2009;19:196-9.
5. Kumar A, Mohanty NK, Jain M, Prakash S, Arora RP. A prospective randomized comparison between early (<48 hours of onset of colicky pain) versus delayed shockwave lithotripsy for symptomatic upper ureteral calculi: a single center experience. J Endourol 2010;24:2059-66.
6. Seitz C, Tanovic E, Kikic Z, Memarsadeghi M, Fajkovic H. Rapid extracorporeal shock wave lithotripsy for proximal ureteral calculi in colic versus noncolic patients. Eur Urol 2007;52:1223-7.
7. Tombal B, Mawlawi H, Feyaerts A, Wese FX, Opsomer R, Van Cangh PJ. Prospective randomized evaluation of emergency extracorporeal shock wave lithotripsy (ESWL) on the short-time outcome of symptomatic ureteral stones. Eur Urol 2005;47:855-9.
8. Tligui M, El Khadime MR, Tchala K, Haab F, Traxer O, Gattegno B, et al. Emergency extracorporeal shock wave lithotripsy (ESWL) for obstructing ureteral stones. Eur Urol 2003;43:552-5.
9. Picozzi SC, Ricci C, Gaeta M, Casellato S, Stubinski R, Ratti D, et al. Urgent shock wave lithotripsy as first-line treatment for ureteral stones: a meta-analysis of 570 patients. Urol Res 2012;40:725-31.
10. Al-Nakshabandi NA. The soft-tissue rim sign. Radiology 2003;229:239-40.
11. Pareek G, Hedican SP, Lee FT Jr, Nakada SY. Shock wave lithotripsy success determined by skin-to-stone distance on computed tomography. Urology 2005;66:941-4.
12. Dindo D, Demartines N, Clavien PA. Classification of surgical complications: a new proposal with evaluation in a cohort of 6336 patients and results of a survey. Ann Surg 2004;240:205-13.
13. Mueller SC, Wilbert D, Thueroff JW, Alken P. Extracorporeal shock wave lithotripsy of ureteral stones: clinical experience and experimental findings. J Urol 1986;135:831-4.
14. Kim HL, Labay PC, Boyarsky S, Glenn JF. An experimental model of ureteral colic. J Urol 1970;104:390-4.
15. Cummings JM, Boullier JA, Izenberg SD, Kitchens DM, Kothandapani RV. Prediction of spontaneous ureteral calculous passage by an artificial neural network. J Urol 2000;164:326-8.
16. Kravchick S, Bunkin I, Stepnov E, Peled R, Agulansky L, Cytron S. Emergency extracorporeal shockwave lithotripsy for acute renal colic caused by upper urinary-tract stones. J Endourol 2005;19:1-4.
