# Supplementary material for: Efficacy of emergency extracorporeal shock wave lithotripsy in the treatment of ureteral stones: a meta-analysis
Source: BMC Urol. 2023 Apr 4;23:56. doi: 10.1186/s12894-023-01226-5 (PMC10074806; doi:10.1186/s12894-023-01226-5)
Supplement: Supplementary file 3 — Additional File 3: Cornelius 2020 [file 12894_2023_1226_MOESM3_ESM.pdf]

Journal of Endourology

© Mary Ann Liebert, Inc.

DOI: 10.1089/end.2020.0384

Downloaded by King's College London Jrnls Section from www.liebertpub.com at 11/29/20. For personal use only.

Journal of Endourology
Immediate shockwave-lithotripsy versus delayed shockwave-lithotripsy after urgent ureteral stenting in patients with ureteral or pyeloureteral urolithiasis: A matched-pair analysis (DOI: 10.1089/end.2020.0384)
This paper has been peer-reviewed and accepted for publication, but has yet to undergo copyediting and proof correction. The final published version may differ from this proof.

Immediate shockwave-lithotripsy versus delayed shockwave-lithotripsy after urgent ureteral stenting in patients with ureteral or pyeloureteral urolithiasis: A matched-pair analysis

Cornelius J.[1#], Zumbühl D.[1#], Afferi L.[1], Mordasini L.[1], Di Bona C.[1], Zamboni S.[1,2], Moschini M.[1], Pozzi E.[3], Salonia. A.[3,4], Mattei A.[1], Danuser H.[1], Baumeister P.[1]

[1] Department of Urology, Luzerner Kantonsspital, Lucerne, Switzerland

[2] Department of Urology, Spedali Civili Hospital of Brescia, University of Brescia, Brescia, Italy

[3] Division of Experimental Oncology/Unit of Urology; URI; IRCCS Ospedale San Raffaele, 20132 Milan, Italy

[4] University Vita- Salute San Raffaele, Milan, Italy

[#] Authors contributed equally in the preparation of the manuscript

Corresponding author: Julian Cornelius, Department of Urology, Luzerner Kantonsspital, Lucerne, Switzerland, Email: julian.cornelius@luks.ch, Phone: +41 412054510

Running head: eSWL versus sSWL after double-J stenting

Keywords: shockwave lithotripsy, ureteral calculi, ureterolithiasis, double-J stent, SWL

Downloaded by King's College London Jrnls Section from www.liebertpub.com at 11/29/20. For personal use only.

Journal of Endourology
Immediate shockwave-lithotripsy versus delayed shockwave-lithotripsy after urgent ureteral stenting in patients with ureteral or pyeloureteral urolithiasis: A matched-pair analysis (DOI: 10.1089/end.2020.0384)
This paper has been peer-reviewed and accepted for publication, but has yet to undergo copyediting and proof correction. The final published version may differ from this proof.

Abstract

Introduction: The most common cause of acute renal colic is a ureteral obstruction caused by ureterolithiasis. Urgent intervention is often necessary due to intractable pain. Early extracorporeal shockwave lithotripsy (SWL) as an alternative treatment option to ureteral stenting becomes forgotten in times of rising Ureterorenoscopy. However, definitive guidelines are lacking which urgent treatment should be preferred in absence of signs of infection. Therefore, we assessed efficacy and safety of early SWL (eSWL) to secondary SWL (sSWL) after urgent ureteral stenting.

Patients and Methods: 104 patients treated between 01/2015 and 11/2017 for obstructive ureterolithiasis were matched regarding stone size, stone localization and assigned to group eSWL (n= 52) or group sSWL (n= 52). eSWL group received shock waves (without prior ureteral stenting) and sSWL group ureteral stenting within 48 hours from diagnosis. Thereafter, patients in group sSWL were treated with shock waves median 23 ± 14.6 days after ureteral stenting. Stone-free rates, complication rates and reintervention rates were assessed. Univariable and multivariable logistic regression was applied to find predictors of outcomes in the two treatment groups.

Results: Overall there was no statistically significant difference between both groups regarding stone-free rate and complication rate. Reinterventions were more often addressed for patients in group sSWL (*p= 0.05*). eSWL was significantly superior to sSWL regarding stone-free rates for stones between 6-9mm (*p= 0.04).* At the multivariable multinomial logistic regression none of the two treatment modalities was associated with better outcomes. A body mass index ≥30 was associated with a reduced 6-weeks stone-free status (*p= 0.04)*, whereas stones ≥8 mm were associated with an increased need of reintervention *(p= 0.04)*.

Conclusion: eSWL seems to be an effective and safe emergency procedure compared to sSWL after urgent stenting within 6 weeks and should be considered as a treatment option in patients without absolute indications for immediate ureteral drainage.

Downloaded by King's College London Jnls Section from www.liebertpub.com at 11/29/20. For personal use only.
Journal of Endourology
Immediate shockwave-lithotripsy versus delayed shockwave-lithotripsy after urgent ureteral stenting in patients with ureteral or pyeloureteral urolithiasis: A matched-pair analysis (DOI: 10.1089/end.2020.0384)
This paper has been peer-reviewed and accepted for publication, but has yet to undergo copyediting and proof correction. The final published version may differ from this proof.

Introduction

Ureteral and pyeloureteral urolithiasis have a high prevalence among urological emergency patients and leads to renal colic caused by ureteral obstruction[1]. These patients can be treated by different procedures due to the individual situation. Absolute indications for an urgent ureteral drainage are patients with obstructing ureteral stones and acute urinary tract infection or urinoma as well as anuria because of a single kidney[2]. However, even in the absence of these indications a ureteral drainage is often used as primary procedure in patients with analgesic not compensable colic pain.

In general, stones ≤ 4mm can be treated by medical (expulsion) therapy. Patients with larger stones or with colics refractory to medication therapy can be treated by one of the following options: draining the collecting system by double-J stent (or percutaneous nephrostomy) followed by secondary URS or SWL, early or delayed SWL and early or delayed URS. Important factors predicting the selection of above mentioned therapeutical options are stone location, stone size, stone density, patients' comorbidities, as well as patients' preference in cases with more than one equal treatment option[3]. In general, SWL can be recommended for stones located in the upper and middle pole of the kidney, as well as the proximal or distal ureter[2]. Picozzi *et al*. considered early SWL to be a safe and effective primary procedure[4]. SWL as an urgent procedure in patients with acute colic pain can lead to quick pain relief and can lower stone-related morbidity[5].

From our point of view, the main disadvantages of a primary ureteral drainage are stent induced discomfort or complications (f.e. irritative bladder symptoms, hematuria, flank pain or bacteriuria[6]) and the need for a secondary stone therapy with increased anesthesia-/ procedure derived risks and therefore increased health related costs. On the other hand, immediate ureteral drainage leads to rapid pain relief while patients after SWL are at risk of a recurrent colic in 2-4% of cases[7]. However, guidelines are unclear regarding an urgent treatment of ureteral and pyeloureteral urolithiasis.

The aim of our study is to assess whether early SWL (eSWL) within 48 hours and secondary SWL (sSWL) after emergency ureteral stenting within 48 hours show different efficacy and safety outcomes.

Downloaded by King's College London Jrnls Section from www.liebertpub.com at 11/29/20. For personal use only.
Journal of Endourology
Immediate shockwave-lithotripsy versus delayed shockwave-lithotripsy after urgent ureteral stenting in patients with ureteral or pyeloureteral urolithiasis: A matched-pair analysis (DOI: 10.1089/end.2020.0384)
This paper has been peer-reviewed and accepted for publication, but has yet to undergo copyediting and proof correction. The final published version may differ from this proof.

Patients and Methods

Study population

Patients treated for an obstructive urolithiasis between 01/2015 and 11/2017 were retrospectively screened for their primary procedure using the clinical electronic health record system of our hospital (EPIC hyperspace, EPIC System Corporation®).

A total of 104 patients with ureterolithiasis who underwent either eSWL (without ureteral stenting) or sSWL after urgent ureteral drainage within 48 hours after first presentation at the emergency department were included. Patients were matched regarding stone size (≤5 mm; 6-9 mm; >10 mm) and stone location (proximal or distal ureter) and grouped regarding their primary surgical procedure into eSWL (n=52) or sSWL (n=52). Subanalysis was performed for stone size and stone localization.

Diagnosis of urolithiasis was made by unenhanced computed tomography (CT) and abdominal X-Ray, measuring the largest available stone diameter each time. All patients were afebrile and had no signs of infection in blood and urinary analysis. Excluded from the study were patients with previous surgical treatment for the same calculi, ureteral pathologies or clotting disorders.

SWL was performed using the Storz Modulith Lithotriptor at a shockwave rate of 60 – 90 Hz. A maximum number of 3000 shockwaves and a maximum energy of 9 were delivered in each session for patients with ureteral stone disease while patients with pyeloureteral stones received a maximum of 2500 shockwaves and a maximum energy level of 7. A precise focus was selected until (partial) fragmentation was observed. The treatment was then continued with an extended focus to ensure optimal fragmentation. All patients were treated under a need-based sedo-analgesia by the collogues in anesthesia (intravenous Metamizol, NSAIDs and opioids). Ureteral drainage was performed in general anesthesia by insertion of a 6 French double-J-stent for all patients in the sSWL group. Stent removal was carried out routinely 6 weeks after sSWL in our outpatient clinic under local anesthesia (Instillagel®) and was not included in our evaluation as an independent intervention.

Downloaded by King's College London Jnls Section from www.liebertpub.com at 11/29/20. For personal use only.
Journal of Endourology
Immediate shockwave-lithotripsy versus delayed shockwave-lithotripsy after urgent ureteral stenting in patients with ureteral or pyeloureteral urolithiasis: A matched-pair analysis (DOI: 10.1089/end.2020.0384)
This paper has been peer-reviewed and accepted for publication, but has yet to undergo copyediting and proof correction. The final published version may differ from this proof.

After SWL patients of both groups received a standard needs-based analgesic therapy with Metamizol and Diclofenac, as well as an expulsive treatment with Tamsulosin for the maximum duration of 6 weeks. A follow-up visit routinely took place 6 weeks after eSWL or sSWL and stone passage was assessed by abdominal X-Ray and ultrasound. Any residual fragment detected during the follow-up visit was considered as SWL failure. Complications were assessed according to the Clavien-Dindo classification[8].

Primary and secondary endpoints

Primary endpoint was defined as stone-free rate 6 weeks after eSWL or sSWL. Secondary endpoints were complications and the reintervention rate within the observation period of 6 weeks.

Statistical analysis

Descriptive statistics are reported as mean ± standard deviation (SD). Normal distribution was tested using the Shapiro-Wilk test. To compare independent variables, Student's t-test and Fisher´s Exact test were addressed. A conventional p-value of ≤ 0.05 was considered as statistically significant. For the prediction of stone-free rates, reinterventions and complications dependent variables were tested by univariable and multivariable logistic regression. Statistical analysis was carried out with Prism GraphPad Version 7.0 (GraphPad Software, La Jolla, California, USA).

This study was approved by the national research committee "Ethikkomission Nordwest- und Zentralschweiz" under the registry 2019-00155. We hereby confirm that all methods were performed in accordance with the relevant guidelines and regulations.

Results

Patients and stone data are shown in **Table 1**. After propensity match scoring, 52 patients treated by eSWL and 52 by sSWL after urgent ureteral double-J stenting due to analgetic not compensable flank pain were obtained. 80 patients (78%) were male, 24 patients (22%) female. Mean age was 48,1 ± 14,9 years in the eSWL group and 54,6 ± 13,2 years in the sSWL group. Mean stone size was 7mm (range: 5-12 mm) in both study groups; 70% of stones were located in the proximal ureter (n=40) and 30% in the distal

Downloaded by King's College London Jnls Section from www.liebertpub.com at 11/29/20. For personal use only.

Journal of Endourology
Immediate shockwave-lithotripsy versus delayed shockwave-lithotripsy after urgent ureteral stenting in patients with ureteral or pyeloureteral urolithiasis: A matched-pair analysis (DOI: 10.1089/end.2020.0384)
This paper has been peer-reviewed and accepted for publication, but has yet to undergo copyediting and proof correction. The final published version may differ from this proof.

ureter (n=12) in both study groups. Mean time from patients' first presentation at the emergency department until initial procedure was 20,3 ± 13 hours for the eSWL group and 13,3 hours ± 7,1 hours for the sSWL group. Mean time between ureteral stenting and sSWL was 23 ± 14,6 days. sSWL was carried out with double-J stent in situ for all patients.

Overall, we did not find any significant difference between eSWL and sSWL regarding stone-free rate and complication rate. 32/48 patients (67%) in group eSWL and 22/45 patients (49%) in group sSWL were stone free after 6 weeks (p=0.10) (**Figure 1A**). Clavien-Dindo Grade 1 and 2 complications occurred in 17/51 (33%) cases of the eSWL group and in 18/52 (35%) cases of the sSWL group (p>0.99) (**Figure 1B**). In group eSWL 16/17 patients (94%) needed additional brief analgesic therapy (oral opioids) which is classified as Grade 1 complications according to the Clavien-Dindo classification. 1 case of postinterventional fever was recorded (Clavien-Dindo Grade 2). In group sSWL 16/18 patients (89%) needed additional brief opioid therapy (Clavien-Dindo Grade 1) and 2 cases of fever occurred (Clavien-Dindo Grade 2). No Clavien-Dindo Grade 3-5 complications and no renal hematomas were reported in both groups.

Reintervention rate was significantly higher in group sSWL *(p=0.05)*. 17/51 patients (33%) in group eSWL and 27/50 patients (54%) in group sSWL needed a reintervention due to stone persistence and/or recurrent flank pain (**Figure 1C**). No case of postinterventional ureteral stenting was reported in the eSWL group. Reinterventions consisted either of re-SWL or secondary URS. Mean number of total procedures until stone free status was achieved was n= 84 in group eSWL and n=132 in group sSWL, respectively.

In patients after eSWL, 8/17 reinterventions (47%) were carried out as a second SWL session. 5/8 patients (62.5%) were stone free after their second SWL session. 3/8 patients (37.5%) had to undergo secondary URS after ureteral drainage due to stone persistence. 9/17 patients (53%) received a secondary URS after elective double-J stent placement without undergoing a second SWL session.

Interestingly, in group sSWL secondary URS was carried out in 14/27 patients (52%), while a repeated SWL was still performed in 13/27 patients (41%). All 14 patients underwent secondary URS after initial SWL failure were stone-free afterwards. Out of 13

Downloaded by King's College London Jrnls Section from www.liebertpub.com at 11/29/20. For personal use only.
Journal of Endourology
Immediate shockwave-lithotripsy versus delayed shockwave-lithotripsy after urgent ureteral stenting in patients with ureteral or pyeloureteral urolithiasis: A matched-pair analysis (DOI: 10.1089/end.2020.0384)
This paper has been peer-reviewed and accepted for publication, but has yet to undergo copyediting and proof correction. The final published version may differ from this proof.

patients underwent a second SWL session, 1 patient needed further treatment by URS after pigtail insertion due to stone persistence.

The subgroup analyses are reported in **Table 2A** and **Table 2B**. Patients with 6-9 mm stones reached a significantly higher stone-free rate after eSWL in comparison with sSWL (72% vs. 45%, *p=0.04*) (**Figure 2**). Regarding complication- and reintervention rates, no significant difference was detected within the subgroups. The reintervention rate in patients with eSWL versus sSWL was insignificantly different for stones sized 5 mm (20% vs. 50%; p=0.35) and 6-9 mm (33% vs. 58%; p=0.08) and almost equal for calculi sized >10 mm (50% vs. 43%; p>0.99). More minor complications were detected after sSWL when compared to eSWL (20% vs. 40%; p=0.63) in patients with stones sized 5mm. For patients with stones sized 6-9 mm (35% vs. 38%; p>0.99) and ≥ 10 mm (38% vs. 13%; p=0.57) complication rates were almost equal or slightly lower for group sSWL (**Table 2A**).

In addition, subanalysis for stones in the proximal or distal ureter showed no significant difference between both groups regarding stone-free rate, complication rate and reintervention rate. Stone-free rate after 6 weeks was slightly higher for patients treated with eSWL for calculi located in the proximal (61% vs. 43%; p=0.16) or the distal ureter (83% vs. 70%; p= 0.62) while reintervention rate was higher for stones in the proximal (36% vs. 53%; p=0.17) and distal ureter (25% vs. 58%; p= 0.21) in the sSWL group. Primary ureteral drainage prior to SWL is associated with an insignificantly increase of minor complications for calculi located in the distal ureter (42% vs. 58%; p= 0.68) but not for calculi located in the proximal ureter (33% vs. 28%; p>0.99) **(Table 2B)**.

At univariable logistic regression analysis, only stone localization of the distal ureter was significantly associated with stone-free rates after 6 weeks (OR 3.12; 95% confidence interval (CI) 1.04-9.39; p= 0.04). At multivariable logistic regression analysis, a BMI ≥ 30 kg/m^2 was found to be an independent predictor of a lower stone-free rate after 6 weeks after SWL (OR 0.18; 95% CI 0.03-0.98; *p=0.04*) (**Table 3A**). Multivariable logistic regression showed also that stone diameters ≥ 8mm are significantly associated with a higher reintervention rate (OR 3.45; 95% CI 1.01-11.91; *p=0.04*) **(Table 3B)**, whereas none of the variables tested were able to predict a high risk of complications **(Table 3C)**.

Downloaded by King's College London Jmls Section from www.liebertpub.com at 11/29/20. For personal use only.
Journal of Endourology
Immediate shockwave-lithotripsy versus delayed shockwave-lithotripsy after urgent ureteral stenting in patients with ureteral or pyeloureteral urolithiasis: A matched-pair analysis (DOI: 10.1089/end.2020.0384)
This paper has been peer-reviewed and accepted for publication, but has yet to undergo copyediting and proof correction. The final published version may differ from this proof.

Discussion

Acute flank colic due to obstructive ureteral calculi can be treated depending on stone size and location conservatively, by double-J stenting, nephrostomy, Ureteroscopy or SWL. Double-J stents are widely used as a primary procedure. First introduced in 1980, SWL became a standard treatment option for patients with ureteral or renal calculi especially due to its non-invasive nature, low morbidity and high efficacy. In our study we focused on early SWL and delayed SWL after urgent ureteral stenting. Our data suggest that eSWL compared to urgent ureteral stenting and sSWL is more effective and leads to fewer reinterventions.

Although many institutions used early SWL since years, in 1999 Joshi *et al.*[9] proposing SWL in this setting for the first time. The comparative retrospective analysis of 82 patients showed in situ eSWL superior to ureteral drainage or nephrostomy placement with following in situ SWL. The median success rate after eSWL was 89% compared with 70% after double-J stenting and 54% after placement of a percutaneous nephrostomy. In our study overall stone-free rate of eSWL (67%) within 6 weeks was lower than reported for in situ SWL but still insignificantly superior when compared to sSWL (49%). Previously double-J stenting was furthermore associated with a significantly higher probability of reinterventions in our cohort. The higher reintervention rate might be explained by a lower effectiveness in stone passage after SWL when the stent is in situ during the procedure compared with no stenting or percutaneous nephrostomy (PCN)[10]. In line, our data support that there is no benefit of ureteral stenting in terms of complications in patients assigned for SWL in an urgent setting although complication rates were slightly lower in patients with stones ≥10mm undergoing sSWL. Kumar *et al.*[11] prospectively enrolled 160 patients comparing early SWL within 48 hours to delayed SWL (without ureteral stenting). After 3 months stone-free rate was 86% for early SWL and 76% for delayed SWL (p=0.34). Mean time for stone clearance was significantly shorter within the early SWL group when compared to delayed SWL (10.2 days vs. 21.1 days; p=0.01). Supporting especially our findings on the safety profile of eSWL, a cumulative analysis identified 4 randomized controlled studies and 2 retrospective case-control studies comparing eSWL and delayed SWL. eSWL was not only superior to delayed treatment in

Downloaded by King's College London Jrnls Section from www.liebertpub.com at 11/29/20. For personal use only.

This paper has been peer-reviewed and accepted for publication, but has yet to undergo copyediting and proof correction. The final published version may differ from this proof.

terms of stone-free rate (OR 2.2; 95% CI 1.55-3.17; p< 0.001) but also in terms of the need of auxiliary procedures (OR 0.49; 95% CI 0.33-0.72; p< 0.001) with comparable complication rates for both techniques. Findings show high efficacy rates with a relatively low risk for complications highlighting that in the literature the definition for immediate treatment varies between 6 and 72 hours[5]. Regardless of ureteral stenting, time to SWL procedure is known to be relevant. Kumar *et al.*[11] found that a timeframe >48 hours after onset of colic pain to SWL is associated with lower stone clearance rates, higher reintervention rates and a greater need for auxiliary procedures.

In 2008, Musa *et al.*[12] published a prospective-randomized series of 120 patients comparing elective SWL outcomes between patients previously stented and patients without a double-J stent. Stone-free rates were higher in the unstented group, while more residual fragments were detected by X-ray within patients who received a double-J stent prior to SWL. Furthermore, patients complained about typical side effects of double-J stenting like urinary frequency, urgency pain and hematuria. Also, in our series, double-J stenting was associated with an insignificantly but higher risk for minor complications. This might be associated with above mentioned well-known and common side effects of double-J stents (e.g. flank pain and bladder irritation)[6]. In line with these findings we were able to show that in case of an urgent intervention due to intractable pain and especially in the presence of stones sized up to 1cm, ureteral stenting prior to SWL has no statistically significant benefit regarding stone-free rate. On the contrary, ureteral stenting was associated with a significantly lower stone-free rate in patients with stones sized 6-9mm. Besides in our cohort stone localization and patients' BMI predict stone-free rates, while greater stone dimensions are associated with a high risk of reintervention due to residual fragments along the ureter. A systematic review of eight randomized controlled trials conducted in 2011 shows only a benefit regarding the formation of Steinstrasse when a double-J stent was previously placed, but not for stone-free rate and for the need of auxiliary treatment[13]. Another prospective- randomized study of 186 patients investigating elective SWL with or without prior double-J stenting was conducted in 2006 by El-Assmy *et al*. The authors described no significant benefit of prior ureteral stenting in terms of stone-free and retreatment rates[14]. Eventually our experiences in the urgent setting coincide with the evidence available on elective SWL stone treatment.

Downloaded by King's College London Jrnls Section from www.liebertpub.com at 11/29/20. For personal use only.
Journal of Endourology
Immediate shockwave-lithotripsy versus delayed shockwave-lithotripsy after urgent ureteral stenting in patients with ureteral or pyeloureteral urolithiasis: A matched-pair analysis (DOI: 10.1089/end.2020.0384)
This paper has been peer-reviewed and accepted for publication, but has yet to undergo copyediting and proof correction. The final published version may differ from this proof.

We can summarize that our data support the previously published evidence and that we were able to show that these principles also apply in the urgency setting. eSWL is efficient and safe and should be considered as a treatment option. Nevertheless, patients presenting at the emergency department often receive a double-J stent as primary treatment to relieve the pain caused by ureteral obstruction or SWL capacities are not available.

Despite several strengths, our study is not devoid of limitations. First of all, data have been collected retrospectively, therefore our results might suffer from the biases associated with this study design, although we tried to overcome this condition by performing a matched pair analysis, which at least partially automatically adjusts the treatment populations for baseline characteristics. Second, the number of patients analyzed is limited and a confirmation of our findings should be performed on larger populations. Third, patient within the sSWL group were treated while the ureteral drainage was in situ. This might have led to an underestimation in sSWL efficacy as proposed by Middela et al. [10].

Conclusion

We conclude that for patients presenting with obstructive ureterolithiasis, eSWL is an effective and safe alternative to primary double-J stenting. eSWL should be considered especially for stones sized up to 1cm located either in the distal or proximal ureter. Prospective and randomized clinical trials are needed to validate our findings.

Acknowledgments:

JC, LM, HD, AM and PB conceived the project; JC, DZ and CDB collected the data via the clinical operating system. JC, LA, SZ, EP and MM performed the statistically analyses. JC and DZ contributed equally in the preparation of the manuscript (co-shared first authors). All the co-authors actively contributed to interpret the data and write the manuscript.

Author Disclosure Statement:

All authors certify that they have NO affiliations with or involvement in any organization or entity with any financial interest (such as honoraria; educational grants; participation in speakers' bureaus; membership, employment, consultancies, stock ownership, or other

Downloaded by King's College London Jnls Section from www.liebertpub.com at 11/29/20. For personal use only.
Journal of Endourology
Immediate shockwave-lithotripsy versus delayed shockwave-lithotripsy after urgent ureteral stenting in patients with ureteral or pyeloureteral urolithiasis: A matched-pair analysis (DOI: 10.1089/end.2020.0384)
This paper has been peer-reviewed and accepted for publication, but has yet to undergo copyediting and proof correction. The final published version may differ from this proof.

equity interest; and expert testimony or patent-licensing arrangements), or non-financial interest (such as personal or professional relationships, affiliations, knowledge or beliefs) in the subject matter or materials discussed in this manuscript.

Downloaded by King's College London Jrnls Section from www.liebertpub.com at 11/29/20. For personal use only.
Journal of Endourology
Immediate shockwave-lithotripsy versus delayed shockwave-lithotripsy after urgent ureteral stenting in patients with ureteral or pyeloureteral urolithiasis: A matched-pair analysis (DOI: 10.1089/end.2020.0384)
This paper has been peer-reviewed and accepted for publication, but has yet to undergo copyediting and proof correction. The final published version may differ from this proof.

References

1. Teichman JMH. Clinical practice. Acute renal colic from ureteral calculus. *N Engl J Med*. 2004;350(7):684-693. doi:10.1056/NEJMcp030813
2. Türk C, Knoll T, Petrik a, et al. Pocket Guidelines on urolithiasis. *Eur Urol*. 2014;40(4):362-371. doi:10.1159/000049803
3. Wright PJ, English PJ, Hungin APS, Marsden SNE. Managing acute renal colic across the primary-secondary care interface: a pathway of care based on evidence and consensus. *BMJ Br Med J*. 2002;325(7377):1408-1412.
4. Picozzi SCM, Ricci C, Gaeta M, et al. Urgent shock wave lithotripsy as first-line treatment for ureteral stones: a meta-analysis of 570 patients. *Urol Res*. 2012;40(6):725-731. doi:10.1007/s00240-012-0484-0
5. Arcaniolo D, De Sio M, Rassweiler J, et al. Emergent versus delayed lithotripsy for obstructing ureteral stones: a cumulative analysis of comparative studies. *Urolithiasis*. 2017;45(6):563-572. doi:10.1007/s00240-017-0960-7
6. Damiano R, Oliva A, Esposito C, De Sio M, Autorino R, D'Armiento M. Early and late complications of double pigtail ureteral stent. *Urol Int*. 2002;69(2):136-140. doi:10.1159/000065563
7. Tan YM, Yip SK, Chong TW, Wong MYC, Cheng C, Foo KT. Clinical experience and results of ESWL treatment for 3,093 urinary calculi with the Storz Modulith SL 20 lithotripter at the Singapore general hospital. *Scand J Urol Nephrol*. 2002;36(5):363-367. doi:10.1080/003655902320783872
8. Clavien PA, Barkun J, de Oliveira ML, et al. The Clavien-Dindo classification of surgical complications: five-year experience. *Ann Surg*. 2009;250(2):187-196. doi:10.1097/SLA.0b013e3181b13ca2
9. Joshi HB, Obadeyi OO, Rao PN. A comparative analysis of nephrostomy, JJ stent and urgent in situ extracorporeal shock wave lithotripsy for obstructing ureteric stones. *BJU Int*. 1999;84(3):264-269. doi:10.1046/j.1464-410x.1999.00174.x

Downloaded by King's College London Jnls Section from www.liebertpub.com at 11/29/20. For personal use only.
Journal of Endourology
Immediate shockwave-lithotripsy versus delayed shockwave-lithotripsy after urgent ureteral stenting in patients with ureteral or pyeloureteral urolithiasis: A matched-pair analysis (DOI: 10.1089/end.2020.0384)
This paper has been peer-reviewed and accepted for publication, but has yet to undergo copyediting and proof correction. The final published version may differ from this proof.

10. Middela S, Papadopoulos G, Srirangam S, Rao P. Extracorporeal shock wave lithotripsy for ureteral stones: do decompression tubes matter? *Urology*. 2010;76(4):821-825. doi:10.1016/j.urology.2010.01.051

11. Kumar A, Mohanty NK, Jain M, Prakash S, Arora RP. A prospective randomized comparison between early (<48 hours of onset of colicky pain) versus delayed shockwave lithotripsy for symptomatic upper ureteral calculi: a single center experience. *J Endourol*. 2010;24(12):2059-2066. doi:10.1089/end.2010.0066

12. Musa AAK. Use of double-J stents prior to extracorporeal shock wave lithotripsy is not beneficial: results of a prospective randomized study. *Int Urol Nephrol*. 2008;40(1):19-22. doi:10.1007/s11255-006-9030-8

13. Shen P, Jiang M, Yang J, et al. Use of ureteral stent in extracorporeal shock wave lithotripsy for upper urinary calculi: a systematic review and meta-analysis. *J Urol*. 2011;186(4):1328-1335. doi:10.1016/j.juro.2011.05.073

14. El-Assmy A, El-Nahas AR, Sheir KZ. Is pre-shock wave lithotripsy stenting necessary for ureteral stones with moderate or severe hydronephrosis? *J Urol*. 2006;176(5):2059-2062; discussion 2062. doi:10.1016/j.juro.2006.07.022

Downloaded by King's College London Jnls Section from www.liebertpub.com at 11/29/20. For personal use only.
Journal of Endourology
Immediate shockwave-lithotripsy versus delayed shockwave-lithotripsy after urgent ureteral stenting in patients with ureteral or pyeloureteral urolithiasis: A matched-pair analysis (DOI: 10.1089/end.2020.0384)
This paper has been peer-reviewed and accepted for publication, but has yet to undergo copyediting and proof correction. The final published version may differ from this proof.

Abbreviations Used

BMI - Body mass index

CI - Confidence Interval

CT - computed tomography

eSWL - emergency shockwave-lithotripsy

HUmax- maximal Houndsfield Units

Hz - Herz

OD - Odds Ratio

re-SWL- repeated SWL

SD - Standard Deviation

SWL - extracorporeal shockwave-lithotripsy

sSWL - secondary extracorporeal shockwave-lithotripsy

URS - Ureterorenoscopy

X-Ray - Energetic High-Frequency Electromagnetic Radiation

Downloaded by King's College London Jrnls Section from www.liebertpub.com at 11/29/20. For personal use only.
Journal of Endourology
Immediate shockwave-lithotripsy versus delayed shockwave-lithotripsy after urgent ureteral stenting in patients with ureteral or pyeloureteral urolithiasis: A matched-pair analysis (DOI: 10.1089/end.2020.0384)
This paper has been peer-reviewed and accepted for publication, but has yet to undergo copyediting and proof correction. The final published version may differ from this proof.

Table 1: Patient and stone characteristics of matched pairs (eSWL vs. sSWL)

		**eSWL**<br>n= 52	**sSWL**<br>n= 52	***p-value***
**Patients**	Gender	m= 38<br>f= 14	m= 42<br>f= 10	
	BMI (kg/m^2)	27.3 ± 4.7	27.8 ± 6.0	*p= 0.30*
	Age (years)	48.1 ± 14.9	54.6 ± 13.2	*p= 0.42*
**Stone-characteristics**	Size (mm)	7.1± 1.9	7.2± 1.9	*p> 0.99*
	5 mm	n= 10	n= 10	
	6-9 mm	n= 34	n= 34	
	≥10 mm	n= 8	n= 8	
	HUmax	1046± 263.7	1123± 287.6	*p= 0.46*
	Localisation			
	proximal	n= 40	n= 40	
	distal	n= 12	n= 12	

HUmax: Maximal Hounsfield Units

Downloaded by King's College London Jrnls Section from www.liebertpub.com at 11/29/20. For personal use only.
Journal of Endourology
Immediate shockwave-lithotripsy versus delayed shockwave-lithotripsy after urgent ureteral stenting in patients with ureteral or pyeloureteral urolithiasis: A matched-pair analysis (DOI: 10.1089/end.2020.0384)
This paper has been peer-reviewed and accepted for publication, but has yet to undergo copyediting and proof correction. The final published version may differ from this proof.

Table 2 A: Stone-free-, Complication- and Reintervention rate depending on stone size in patients after eSWL and sSWL

	Stone size	eSWL (n=52)	sSWL (n=52)	*p-value*
**Stone-free rate; n (%)**	5mm	8/10 (80%)	5/9 (56%)	*p= 0.35*
	6- 9mm	23/32 (72%)	13/29 (45%)	*p= 0.04**
	≥ 10mm	1/6 (17%)	4/7 (57%)	*p= 0.27*
**Complication rate; n (%)**	5mm	2/10 (20%)	4/10 (40%)	*p= 0.63*
	6- 9mm	12/34 (35%)	13/34 (38%)	*p> 0.99*
	≥ 10mm	3/8 (38%)	1/8 (13%)	*p= 0.57*
**Re-intervention rate; n (%)**	5mm	2/10 (20%)	5/10 (50%)	*p= 0.35*
	6- 9mm	11/33 (33%)	19/33 (58%)	*p= 0.08*
	≥ 10mm	4/8 (50%)	3/8 (43%)	*p> 0.99*

Downloaded by King's College London Jmls Section from www.liebertpub.com at 11/29/20. For personal use only.

Journal of Endourology
Immediate shockwave-lithotripsy versus delayed shockwave-lithotripsy after urgent ureteral stenting in patients with ureteral or pyeloureteral urolithiasis: A matched-pair analysis (DOI: 10.1089/end.2020.0384)
This paper has been peer-reviewed and accepted for publication, but has yet to undergo copyediting and proof correction. The final published version may differ from this proof.

Table 2 B: Stone-free-, Complication- and Reintervention rate depending on stone localization in patients after eSWL or sSWL

	**Stone localization**	**eSWL (n=52)**	**sSWL (n=52)**	***p-values***
**Stone-free rate; n (%)**	Distal	10/12 (83%)	7/10 (70%)	*p= 0.62*
	Proximal	22/36 (61%)	15/35 (43%)	*p= 0.16*
**Complication rate; n (%)**	Distal	5/12 (42%)	7/12 (58%)	*p= 0.68*
	Proximal	12/40 (33%)	11/40 (28%)	*p> 0.99*
**Reintervention-rate; n (%)**	Distal	3/12 (25%)	7/12 (58%)	*p= 0.21*
	Proximal	14/39 (36%)	20/38 (53%)	*p= 0.17*

Downloaded by King's College London Jnls Section from www.liebertpub.com at 11/29/20. For personal use only.
Journal of Endourology
Immediate shockwave-lithotripsy versus delayed shockwave-lithotripsy after urgent ureteral stenting in patients with ureteral or pyeloureteral urolithiasis: A matched-pair analysis (DOI: 10.1089/end.2020.0384)
This paper has been peer-reviewed and accepted for publication, but has yet to undergo copyediting and proof correction. The final published version may differ from this proof.

Table 3A. Univariable and multivariable logistic regression analyses for the prediction of stone-free status in patients who underwent immediate (eSWL) vs delayed (sSWL) shock wave lithotripsy.

Variables	Stone-free status			
	Univariable analyses		Multivariable analyses	
	OR (CI 95%)	*p* value	OR (CI 95%)	*p* value
**Technique**				
sSWL vs eSWL	0.47 (0.21-1.10)	0.08	0.50 (0.15-1.60)	0.2
**BMI**				
25-29.9 vs 18.5-24.9	0.55 (0.19-1.65)	0.3	0.55 (0.14-2.19)	0.4
≥30 vs 18.5-24.9	0.43 (0.12-1.57)	0.2	0.18 (0.03-0.98)	***0.04***
**Stone dimension**				
≥8 vs <8	0.45 (0.18-1.11)	0.08	0.35 (0.10-1.04)	0.06
**Stone density**				
≥1000 vs <1000	0.47 (0.17-1.29)	0.1	0.55 (0.16-1.84)	0.3
**Stone position**				
Distal vs proximal	3.12 (1.04-9.39)	***0.04***	2.08 (0.47-9.26)	0.2

OR: Odds ratio, CI: Confidence interval

Downloaded by King's College London Jmls Section from www.liebertpub.com at 11/29/20. For personal use only.

Journal of Endourology
Immediate shockwave-lithotripsy versus delayed shockwave-lithotripsy after urgent ureteral stenting in patients with ureteral or pyeloureteral urolithiasis: A matched-pair analysis (DOI: 10.1089/end.2020.0384)
This paper has been peer-reviewed and accepted for publication, but has yet to undergo copyediting and proof correction. The final published version may differ from this proof.

Table 3B. Univariable and multivariable logistic regression analyses for the prediction of need of re-intervention in patients who underwent immediate (eSWL) vs delayed (sSWL) shock wave lithotripsy.

**Variables**	**Need of reintervention**			
	**Univariable analyses**		**Multivariable analyses**	
	**OR (CI 95%)**	***p* value**	**OR (CI 95%)**	***p* value**
**Technique**				
sSWL vs eSWL	1.44 (0.58-3.59)	0.4	0.93 (0.29-2.92)	0.9
**BMI**				
25-29.9 vs 18.5-24.9	1.77 (0.54-5.72)	0.3	2.81 (0.61-12.84)	0.2
≥30 vs 18.5-24.9	1.26 (0.31-5.20)	0.7	2.65 (0.47-14.87)	0.3
**Stone dimension**				
≥8 vs <8	2.46 (0.97-6.24)	0.05	3.45 (1.01-11.91)	***0.04***
**Stone density**				
≥1000 vs <1000	1.86 (0.60-5.80)	0.2	2.22 (0.62-7.89)	0.2
**Stone position**				
Distal vs proximal	1.03 (0.36-2.98)	0.9	2.58 (0.61-10.92)	0.2

OR: Odds ratio, CI: Confidence interval

Downloaded by King's College London Jrnls Section from www.liebertpub.com at 11/29/20. For personal use only.
Journal of Endourology
Immediate shockwave-lithotripsy versus delayed shockwave-lithotripsy after urgent ureteral stenting in patients with ureteral or pyeloureteral urolithiasis: A matched-pair analysis (DOI: 10.1089/end.2020.0384)
This paper has been peer-reviewed and accepted for publication, but has yet to undergo copyediting and proof correction. The final published version may differ from this proof.

Table 3C. Univariable and multivariable logistic regression analyses for the prediction of complications in patients who underwent immediate (eSWL) vs delayed (sSWL) shock wave lithotripsy.

**Variables**	**Risk of complications**			
	**Univariable analyses**		**Multivariable analyses**	
	**OR (CI 95%)**	***p* value**	**OR (CI 95%)**	***p* value**
**Technique**				
sSWL vs eSWL	1.00 (0.44-2.24)	1	0.44 (1.45-1.33)	0.1
**BMI**				
25-29.9 vs 18.5-24.9	2.11 (0.70-6.31)	0.1	3.49 (0.88-13.72)	0.07
≥30 vs 18.5-24.9	2.00 (0.55-7.23)	0.3	1.82 (0.39-8.43)	0.4
**Stone dimension**				
≥8 vs <8	0.70 (0.29-1.70)	0.4	0.56 (0.18-1.73)	0.3
**Stone density**				
≥1000 vs <1000	0.90 (0.35-2.28)	0.8	0.94 (0.32-2.76)	0.9
**Stone position**				
Distal vs proximal	2.33 (0.91-5.93)	0.07	1.25 (0.36-4.36)	0.7

OR: Odds ratio, CI: Confidence interval

Downloaded by King's College London Jnls Section from www.liebertpub.com at 11/29/20. For personal use only.
Journal of Endourology
Immediate shockwave-lithotripsy versus delayed shockwave-lithotripsy after urgent ureteral stenting in patients with ureteral or pyeloureteral urolithiasis: A matched-pair analysis (DOI: 10.1089/end.2020.0384)
This paper has been peer-reviewed and accepted for publication, but has yet to undergo copyediting and proof correction. The final published version may differ from this proof.

Figure Legends

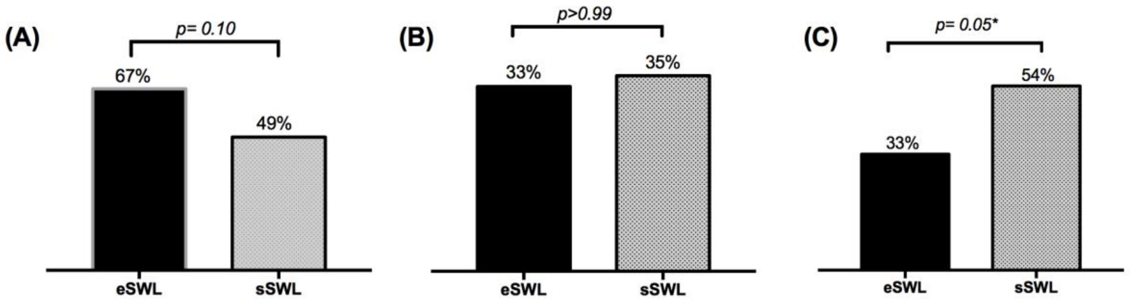

Figure 1:
(A) Stone-free rate in patients after eSWL or sSWL
(B) Complication rate within 6 weeks in patients treated by eSWL and sSWL respectively
(C) Reintervention rate in patients after eSWL and sSWL

Figure 1:

(A) Stone-free rate in patients after eSWL or sSWL

(B) Complication rate within 6 weeks in patients treated by eSWL and sSWL respectively

(C) Reintervention rate in patients after eSWL and sSWL

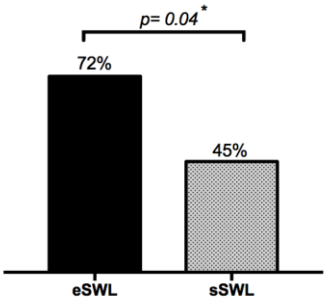

Figure 2: Stone-free rate for calculi sized 6-9 mm in group eSWL and group sSWL

Figure 2: Stone-free rate for calculi sized 6-9 mm in group eSWL and group sSWL

Downloaded by King's College London Jrnls Section from www.liebertpub.com at 11/29/20. For personal use only.
Journal of Endourology
Immediate shockwave-lithotripsy versus delayed shockwave-lithotripsy after urgent ureteral stenting in patients with ureteral or pyeloureteral urolithiasis: A matched-pair analysis (DOI: 10.1089/end.2020.0384)
This paper has been peer-reviewed and accepted for publication, but has yet to undergo copyediting and proof correction. The final published version may differ from this proof.
