# Supplementary material for: Efficacy of emergency extracorporeal shock wave lithotripsy in the treatment of ureteral stones: a meta-analysis
Source: BMC Urol. 2023 Apr 4;23:56. doi: 10.1186/s12894-023-01226-5 (PMC10074806; doi:10.1186/s12894-023-01226-5)
Supplement: Supplementary file 4 — Additional File 4: Joshi 1999 [file 12894_2023_1226_MOESM4_ESM.pdf]

BJU International (1999), **84**, 264–269

A comparative analysis of nephrostomy, JJ stent and urgent *in situ* extracorporeal shock wave lithotripsy for obstructing ureteric stones

H.B. JOSHI, O.O. OBADEYI and P.N. RAO
Lithotriptor Unit, South Manchester University Hospital NHS Trust, Withington Hospital, Manchester, UK

Objectives To determine the optimal method of treatment for ureteric stones causing complete obstruction, treated by insertion of a JJ stent or a nephrostomy tube, followed by extracorporeal shock wave lithotripsy (ESWL) or by urgent *in situ* ESWL if readily available.

Patients and methods The study comprised a retrospective analysis of 82 consecutive patients who presented with ureteric stones causing complete obstruction. Twenty-six had a percutaneous nephrostomy (PCN, group 1) and 40 had a JJ stent (group 2) placed to relieve the obstruction, and the stones were subsequently treated by ESWL. Sixteen patients underwent urgent *in situ* ESWL without recourse to either a JJ stent or a PCN (group 3). The choice of the procedure was not determined by stone size, site or other factors, but mainly by the attending surgeon's preference or the availability of urgent ESWL. The success rate was measured by the disintegration of the stone and spontaneous passage after ESWL; failure was defined as the need for additional procedure(s) for stone extraction.

Results Urgent *in situ* ESWL (group 3) had a median (95% confidence interval) success rate of 81 (54–96)%, compared with 70 (53–83)% in group 2 and 54 (33–73)% in group 1.

Conclusion If facilities are available, urgent *in situ* ESWL appears to be the choice of treatment for obstructing ureteric stones. If such facilities are not available, a JJ stent may offer better success than a PCN. A prospective controlled trial is necessary to confirm these findings.

Keywords Nephrostomy, JJ stent, ESWL, obstruction, ureteric stone

Introduction

ESWL and ureteroscopy are currently the mainstay of treatment for ureteric stones. When a stone causes complete obstruction, it is essential to relieve the obstruction first by inserting a percutaneous nephrostomy tube (PCN) or a JJ ureteric stent, with or without pushing the stone back into the kidney. The stone is then subsequently treated by either ureteroscopy or ESWL. Another less invasive option is urgent *in situ* ESWL, which has the advantage of dealing with the obstruction and the stone at the same time. Obviously, when stones are associated with sepsis, the obstruction should be relieved first and the infection dealt with before treating the stone. However, in cases not associated with sepsis, it is unclear which of the three options is the best and whether insertion of a PCN or a JJ stent would improve the outcome. We report our experience of managing obstructing ureteric stones by these three methods.

Accepted for publication 31 March 1999

Patients and methods

The records of all the patients referred to our department with ureteric stones between June 1994 and January 1997 were reviewed. Of the 370 patients reviewed, 85 patients satisfied the criteria for completely obstructing ureteric stones based on the appearances on IVU at the time of presentation. In a clinical situation, to observe the uniformity in the degree of obstruction caused by the stone, only those patients were considered who satisfied the following IVU criteria of obstruction: (i) Delayed visualization and dilatation of the pelvicalyceal system and ureter above the level of the stone, with no contrast medium beyond the stone in the ureter; or (ii) a persistent and dense nephrogram with non visualization of the collecting system.

The initial management of these patients was determined by the referring clinicians, based on their routine individual practices to deal with obstructing ureteric stones, some preferring a PCN and some a JJ stent. Hence, there were two groups of clinicians; one referring all their patients with a PCN and other group with a JJ stent. In some patients it was possible to perform an

© 1999 BJU International

urgent *in situ* ESWL, because the patients were urgently referred before an intervention to relieve the obstruction, and there was a place available on the ESWL list to perform the procedure. Which of the three procedures was used was determined by the practice of the clinicians and the facilities, and was not based on the size, site or other characteristics of the stone. Thus the three groups were: group 1, urgent PCN to relieve the obstruction followed by *in situ* ESWL; group 2, urgent insertion of JJ stent to relieve the obstruction, with or without an attempt at ureteroscopy or 'push-back', followed by *in situ* ESWL; and group 3, urgent *in situ* ESWL with no recourse to any other prior intervention.

All the stones except two were treated by ESWL using the Siemens Lithostar Multiline (Siemens GmBh, Erlangen, Germany). The remaining two patients underwent the treatment on the Siemens Lithostar Plus lithotripter. ESWL was given on a day-case basis and each patient received an indomethacin suppository (100 mg) 30 min before the treatment. A supplemental injection with pethidine was given if the patient experienced pain during treatment. After each session of ESWL every patient had a plain X-ray of the kidney, ureter and bladder (KUB) taken on the same day to assess fragmentation. Usually no more than two sessions of ESWL were used; however, if there was evidence of partial fragmentation at the end of the second session on a KUB and the fragment(s) was >4 mm, further sessions were given. The treatment was considered a success if there was fragmentation of the stone after ESWL, with spontaneous passage of fragments, i.e. a stone-free ureter with no additional intervention.

Treatment was considered a failure if there was no fragmentation, or additional intervention other than ESWL was required to clear the stone fragments. In patients with a PCN, a nephrostogram was taken to confirm the relief of obstruction by free flow of contrast medium in to the bladder, before removal of the nephrostomy tube. In patients in groups 2 and 3, fragmentation and clearance of fragments was monitored by the clinical improvement and relief of symptoms, with a record of the passage of stone fragments and regular follow-up KUB. In patients undergoing urgent *in situ* ESWL, if there was any doubt about the relief of obstruction because fragments did not progress (on a KUB) or there were symptoms suggestive of obstruction, urgent IVU was undertaken. In all patients where the treatment failed, additional procedures were performed including ureteroscopy with or without electrohydraulic lithotripsy (EHL), insertion of a JJ stent and 'push-back' (for groups 1 and 3) followed by ESWL, percutaneous/antegrade removal or open ureterolithotomy.

Means were calculated for the age distribution, stone size and number of ESWL sessions given in each group (including subgroups of successes and failures) and compared using a one-way ANOVA on log-transformed data, while the sex distribution was compared using the chi-square test. The 95% CIs were calculated for age, stone size, ESWL sessions and successful outcome in each group, for comparison.

Results

Of the 85 patients, follow-up data were available for 82 (58 men and 24 women, mean age 49.9 years, SD 16.34, range 21–88); 26 (32%) patients had a PCN inserted (group 1), 40 (49%) had a JJ stent inserted (group 2) and 16 (19%) received urgent *in situ* ESWL (group 3). In group 2, ureteroscopy had failed in seven patients before insertion of a JJ stent, because there were difficulties in reaching the stone or the stone slipped backwards. Four had the stone successfully pushed back with the insertion of the stent and 25 had insertion of a JJ stent only. In four patients in this group the precise details of the operation were unclear at the time of referral.

Table 1 shows the demographic characteristics of the patients, stones and ESWL sessions, with a comparison of the age of the patients, the percentage distribution of gender, and stone size and site. There was no statistically significant difference in age ($P=0.424$), sex ($P=0.914$, $\chi^2=0.179$) and stone size ($P=0.566$) among the three groups. The mean (range) stone size in the whole group was 8.98 (4–25) mm. Of the 82 patients treated, 46 (52%) had stones in the upper, 19 (23%) in the mid and 17 (21%) in the lower ureter. Table 1 also shows the number of ESWL sessions used in each group; 58 (71%) received $\leqslant 2$ sessions, while 24 (29%) received >2 sessions. The mean stone size where >2 sessions were used was 11.99 mm. The failure rate for patients undergoing $\leqslant 2$ sessions was 26%, for three sessions was 47% and for more sessions was 56%. Table 1 also compares the mean number of ESWL sessions for each group, with a separate distribution of the number of sessions given to those who succeeded and those who failed. There was no significant difference in the number of sessions used in the three groups including the subgroups of success and failure ($P=0.094$ overall, for success $=0.313$ and for failure $=0.132$). Figure 1 shows the final result after ESWL treatment for each group; urgent *in situ* ESWL (group 3) appeared to have best outcome, followed by JJ stenting, with the lowest rate for PCN. Only the difference between groups 1 and 3 was significant; the difference between the others was not. The overall success of ESWL of stones in the upper, mid and lower ureter was compared for all patients. ESWL was the most successful (72%) with stones in the upper ureter, followed by the mid (63%) and lower (59%) ureter.

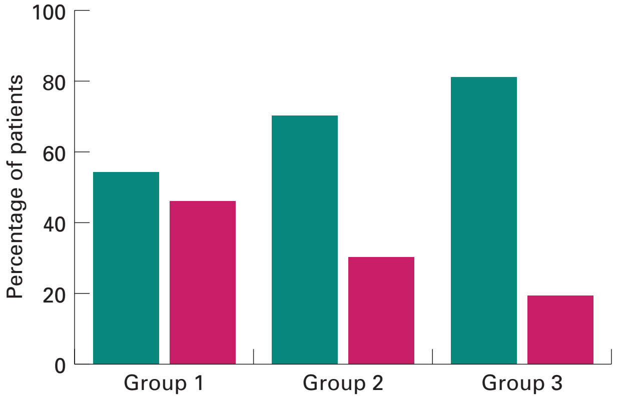

Fig. 1. The proportion of patients in whom treatment was successful (green) or failed (red) in each group. The overall 95% CIs for success groups 1–3, respectively, were 33–73%, 53–83% and 54–96%.

Three of 40 patients in group 2 developed systemic sepsis after attempted endoscopic manipulation or JJ stent insertion and 'push-back', while one patient in group 1 had a deterioration of his sepsis after insertion of a PCN. In five of the 26 patients who had a PCN inserted, clinical and laboratory evidence of systemic sepsis was the main reason for using this method to relieve the obstruction. There was no incidence of sepsis in patients in who underwent urgent *in situ* ESWL.

A secondary intervention was needed in those patients in whom the stone did not disintegrate or the fragments failed to be expressed spontaneously. All these patients were considered failures (Table 1). Failures in group 3 were managed with ureteroscopy (one patient) or 'push-back' with insertion of the stent and ESWL (two patients), while eight patients in group 1 and 2 required more extensive interventions (PCNL and antegrade removal or open ureterolithotomy in one). The mean duration between stenting and undergoing the first session of ESWL was >2 weeks (range 4 days to 6 weeks), while that for insertion of a PCN and the first session of ESWL was just over 9 days (range 3 days to 2 weeks). The insertion of the JJ stent and PCN was performed within a mean of 3 days and 24 h, respectively, from diagnosis. The delay between the intervention and ESWL was caused by factors such as the time for recovery after intervention and convenient arrangements for ESWL at the lithotripter unit. Of 16 patients who underwent urgent *in situ* ESWL, 13 did so after a mean of 3 days (range 1–6) from diagnosis; the remaining three had ESWL 10–14 days after diagnosis. The composition of the stone could be assessed in only six patients. Calcium and oxalate formed most of the stones, with small quantities of ammonium phosphate (three patients) and cystine (one patient).

Table 1 The distribution of patients and treatments, a comparison of ESWL sessions in each group, and the secondary interventions used for those in whom treatment failed

	Group		
Characteristic or variable	*1 (PCN)*	*2 (JJ stent)*	*3 (ESWL)*
Mean (range) age (years)	47.5 (22–75)	49.6 (21–85)	54.3 (29–88)
Men (%)	18 (31)	28 (48)	12 (21)
Women (%)	8 (33)	12 (50)	4 (17)
Mean (95% CI) stone size (mm)	9.0 (7.4–10.9)	9.3 (8.3–10.5)	8.2 (6.5–10.2)
Stone site in ureter			
Upper	9	31	6
Mid	12	4	3
Lower	5	5	7
Mean (range) shock waves	4200 (2500–5000)	4200 (3000–5000)	4050 (2500–5000)
ESWL sessions			
⩽2 ($n = 58$)	21	24	13
⩾3 ($n = 24$)	5	16	3
Mean (95% CI)			
Overall ($n = 82$)	2.0 (1.8–2.3)	2.2 (1.9–2.5)	1.7 (1.3–2.2)
Success group ($n = 55$)	1.8 (1.5–2.2)	1.9 (1.6–2.3)	1.5 (1.1–2.1)
Failure group ($n = 27$)	2.3 (1.9—2.8)	3.0 (2.4–3.7)	2.3 (1.3–4.1)
Secondary intervention ($n = 27$)			
Ureteroscopy (with EHL)	2	8	1
JJ stent + push-back + ESWL	6	–	2
PCNL and antegrade removal	3	4	–
Open ureterolithotomy	1	–	–

Discussion

ESWL is the least invasive and best accepted treatment for ureteric stones [1–5]. *In situ* ESWL has been shown to be effective for ureteric stones at all levels [6,7] including lower ureteric stones, where stones were disintegrated in 62–93% of cases [4,6,8,9], although comparison of the data is difficult because there are unspecified degrees of obstruction, variations in the treatment policy, the lithotripter used and the definitions of success or failure.

Along with the physical properties of the stone, e.g. size and composition, the degree of obstruction is an important factor which determines the success of fragmentation [8,9]. The two main treatment objectives are relief of obstruction, with the treatment of sepsis if present, and disintegration or removal of the stone. There are various approaches to address both objectives, each with advantages and disadvantages. Ureteroscopy is one option which can relieve the obstruction and simultaneously help extract or fragment the stone. However, it has limitations, as it is more suitable for lower ureteric stones than for the stones in mid and upper ureter. It is an invasive procedure, usually requiring general or regional anaesthesia, and may produce significant complications, e.g. perforation, stricture and sepsis in up to 62% [10–12], especially in an obstructed system. Ureteroscopy failed in seven of the present patients before JJ stent insertion and two patients developed sepsis after the procedure. Hence this modality should be used carefully, especially when other less invasive options are available.

The other two approaches are relief of obstruction by insertion of a PCN or a JJ stent and disintegration of the stone later by *in situ* ESWL. Insertion of a PCN under local anaesthesia is relatively less invasive. The drainage of the kidney is considered to be better than with a JJ stent and it can be used to drain pus if present [13]. In the present series it was the preferred treatment in five patients who had clinical and laboratory evidence of sepsis at the time of presentation. It was also used for two further patients who developed sepsis after an endourological procedure. There are other advantages of the PCN, e.g. localization of a radiolucent stone at the time of ESWL (one patient in the present series), and the ability to confirm the completeness of fragmentation and relief of obstruction using a nephrostogram. The potential disadvantages are leakage, dislodgement of the tube, the need to manage the stoma and the introduction of infection. Systemic sepsis may occur after the insertion of a PCN [14], as seen in one patient in this series.

Insertion of a JJ stent is a more invasive procedure, usually requiring general or regional anaesthesia. It can be technically difficult, particularly in an acute and completely obstructed system. However, if successful, in addition to relieving obstruction it may produce additional benefits. It has been shown that repositioning a stone in the renal pelvis or passing a stent alongside the stone improves the success rate of subsequent ESWL [3,4,10,15,16]. It may also help in locating the stone at the time of ESWL [6]. However, apart from complications such as ureteric perforations and failure to pass the stent in some cases, it may increase the risk of urosepsis [13]. Furthermore, there are doubts about its efficacy in decompressing the kidney when compared with a nephrostomy [13]. The presence of the stent may hinder the passage of stone fragments [8,13,17].

In the present series, seven patients in group 2 had the stone successfully pushed back during placement of the stent; 25 patients had only the stent placed, with no 'push-back'. By the time of the first session of ESWL (>5 days after stent insertion) all the stones which were previously pushed back had slipped down to the ureter. Hence, unless ESWL treatment is given as soon as possible (within 2–3 days) after stent placement, the potential advantages of the 'push-back' may be lost, as the stone is likely to migrate into the ureter.

Urgent *in situ* ESWL, being the least invasive procedure and which can be carried out with no need for anaesthesia, is an attractive proposition. In association with stone disintegration, it can simultaneously relieve obstruction. Even when disintegration may be partial after the first session of ESWL, the obstruction is often relieved, as shown by the disappearance of residual contrast medium above the stone and into the bladder in some of the present patients. *In situ* ESWL has been shown to be effective for stones in all parts of the ureter [2–7,9] and in the present series there were no cases of sepsis or other significant complications afterward. Hence it is an attractive treatment option, provided that facilities are available to use in urgent cases.

It is clear from the results of the present study that success after urgent *in situ* ESWL (81%) was greater than after the other two treatments, although the difference was significant only when compared with PCN. Although the success rate after insertion of a JJ stent (70%) was higher than for PCN (54%) the difference was not significant. Considering all its other advantages, and being the least invasive procedure, urgent *in situ* ESWL appears to be the preferred treatment option. A similar conclusion was reached by Chang *et al.* [8] for upper ureteric stones causing varying degrees of obstruction.

The relief of the obstruction seemed to correlate with the fragmentation and progression of the fragments after ESWL, as seen from nephrostograms taken in group 1. However, it was difficult to compare the rapidity of the relief of obstruction after ESWL for the three groups, due

to practical limitations, e.g. it cannot be reliably assessed in group 2 (JJ stent) until the stent is removed, which was performed by the referring clinicians. We accept that although desirable, function of the kidney after ESWL was not studied (by IVU/renogram) in some of the patients with a successful outcome. With the relief of symptoms, complete clearance of the fragments on KUB and retrieval of all/some of the spontaneously passed fragments after ESWL, it was felt appropriate to assume that the obstruction was relieved and hence not to repeat the IVU. Most of these patients had no assessment of renal function before the stone episode and it would not have been possible to compare renal function before and after treatment.

The factors that may affect the success of disintegration by *in situ* ESWL in each of these groups are worth examining. In general, stone disintegration can be considered to be based on three important factors: (i) stone factors (size/bulk, site and composition); (ii) *technical (ESWL) factors* (localization systems, machine used, power range, treatment policy); and (iii) *physical factors* (the presence of fluid and an expansion chamber around the stone, degree of impaction and obstruction, external pressure and other factors affecting the transmission of shockwave energy to the stone, e.g. the size of the patient). In the present study, as factors (i) and (ii) were essentially the same in all three groups, physical factors may have been responsible for the different rates of success among the three groups. Although the composition of the stone, which could influence fragmentation, was unknown for most stones, the X-ray appearances of the stones in the three groups were similar. As all the patients were from the same geographical area and as there were no differences in age, sex and race in the three groups, it is reasonable to assume a similar stone composition in all groups.

It has been postulated that creating an artificial expansion chamber and wet capillary slit is important for better fragmentation during ESWL [15]. This can be achieved by either 'push-back' or placement of a ureteric stent, thus increasing the success rate [4,18]. However, this view has been challenged by some, who showed that the results of treatment are similar whether the stone is pushed back or treated *in situ*, with or without a stent [19]. In the present study, although stenting gave better success rates than a PCN, the results were no better than *in situ* ESWL alone. It is probable that the presence of a stent resulted in a reduction of the shock-wave energy reaching the stone [8], with a resultant decrease in efficiency. Furthermore, the presence of a stent has been shown to cause ureteric constriction [8] and oedema of the wall [4], both of which may reduce the chance of successful fragmentation or the passage of fragments after ESWL. In the present study, more patients in group 2 required three or more sessions of ESWL than in group 1 and 2, providing further support to the view that the presence of a stent may reduce the efficacy of ESWL.

Although a PCN may not produce the additional deleterious effects at the site of the stone produced by a JJ stent, it may introduce air into the system. Because air absorbs or reflects the shock-wave energy [15], the efficacy of ESWL may be diminished. Furthermore, the 'collapse' of the collecting system above the stone may also contribute to its relatively low success rate.

In patients undergoing urgent *in situ* ESWL, urine (water) is present in the ureter above the stone and there are no associated factors which may diminish the efficacy of ESWL energy. Other important factors which may influence the success of ESWL are ureteric oedema and later, peri-ureteric fibrosis, as they may lead to energy absorption within multiple interfaces [15]. The degree of wall reaction may depend on the time the stone is resident in the ureter [4]. If the stone is treated by urgent *in situ* ESWL, this energy-absorbing effect from progressive wall changes can be minimized, thus resulting in an improved success rate. This advantage might have been lost by delayed treatment in groups 1 and 2. There may be additional (physiologically) important factors, such as the influence of ureteric peristalsis, which are difficult to study in a clinical situation but may have contributed to the observations in this study. It may be argued that clamping the PCN temporarily before ESWL may increase the success rate for PCN, based on this discussion. Further studies are required to investigate this hypothesis.

The position of the stone also requires consideration; in the present series, ESWL gave the best results for stones in the upper ureter, then the mid and lower ureter. This suggests that although ESWL has a definite role in treatment of lower ureteric stones, it may be less successful and in selected cases ureteroscopy may be important [6]. More than two ESWL sessions did not necessarily improve the success rate; indeed, the failure rate increased as more sessions were used. In this context, it is possible that *in situ* ESWL may cause impaction of the stone into the ureteric wall. Hence, if sufficient fragmentation cannot be achieved after two ESWL sessions, further sessions should be used only after careful consideration. Patients in group 3 in whom treatment failed were easily converted to group 1 or 2 and then treated successfully by ESWL afterwards, thus achieving success with relatively less intervention.

Finally, a potential limitation of a retrospective study is the possibility of a selection bias. This could have been minimized in that the treatment of the stone was not based on the age and sex of the patients, or the stone size, site or other characteristics. The relatively few

patients, especially in group 3, may have precluded the differences reaching statistical significance.

Notwithstanding these shortcomings, this study strongly indicates that if facilities are available, urgent *in situ* ESWL appears to be superior to other forms of treatment for obstructing ureteric stones. If such facilities are not available, then insertion of a JJ stent may offer better success than a PCN. A prospective controlled trial with more patients is desirable to substantiate these findings.

Acknowledgements

The authors thank Dr Brian Farragher and Miss Allison Wynn-Davies, who provided valuable assistance in the statistical analysis.

References

1 Lingeman JE, Woods J, Toth PD, Evan AP, McAteer JA. The role of lithotripsy and its side effects. *J Urol* 1989; **141**: 793–7

2 Holden D, Rao PN. Ureteral stones. The results of primary in situ ESWL. *J Urol* 1989; **142**: 37–9

3 Miller K, Fuchs G, Rassweiller J, Eisenberger F. Treatment of ureteral stone disease. The role of ESWL and endourology. *World J Urol* 1985; **3**: 53–7

4 Graf J, Pastor J, Funke PJ, Match P, Senge T. ESWL for ureteral stones: a retrospective analysis of 417 cases. *J Urol* 1988; **139**: 513–7

5 Hendrix AJM, Bierkens AF, Debruyne FMJ. ESWL treatment for mid and proximal ureteral calculi: in situ treatment or push bang technique: A randomised trial; *Proceedings of the 1st European Symposium on Urolithiasis*, Excerpta Medica, Medical Communications BV, Amsterdam, 1990: 172–4

6 Robert M, Delbos O, Guifer J, Grasset D. *In situ* piezoelectric extracorporeal shockwave lithotripsy of ureteric stones: *Br J Urol* 1995; **76**: 435–9

7 Cole RS, Shuttleworth KED. Is extracorporeal shock wave lithotripsy suitable for lower ureteric stones? *Br J Urol* 1988; **62**: 525–30

8 Chang S-C, Hann C, Tsi-Hsu K. Extracorporeal shock wave lithotripsy for obstructed proximal ureteral stones. *Eur Urol* 1993; **24**: 177–84

9 Selli C, Carini M. Treatment of lower ureteral calculi with extracorporeal shock wave lithotripsy. *J Urol* 1988; **140**: 280

10 Morse RM, Resnick MI. Ureteral calculi: natural history and treatment in an era of advanced technology. *J Urol* 1991; **145**: 263–5

11 Kramolowsky EV. Ureteral perforations during ureterorenoscopy: treatment and management. *J Urol* 1987; **138**: 36–8

12 Rao PN, Dube DA, Weightman NC, Oppenheim BA. Prediction of bacteraemia and endotoxemia after endourological procedures for stones. *J Endourol* 1990; **4**: Abstract P-16: S80

13 Watson G. JJ stent versus nephrostomy tube drainage. *Urology News* 1997; **1**: 11–3

14 O'Keefe N, Mortimer A, Rao PN. Severe sepsis following percutaneous or endoscopic procedures for urinary tract stones. *Br J Urology* 1993; **72**: 277–83

15 Muller SC, Wilbert D, Thuroff J, Alken P. Extracorporeal shock wave lithotripsy of ureteral stones: Clinical experience and experimental findings. *J Urol* 1986; **135**: 831–4

16 Naidich JB, Greenberg RW, Benetos FC, Badillo FL, Waldbaum RS. Extracorporeal shock wave lithotripsy for in situ ureteral stones: Comparison of two catheter strategies. *J Endourol* 1991; **5**: 197–9

17 Preminger GM, Kettelhut MC, Elkins SL, Seger J, Fetner CD. Ureteral stenting during extracorporeal shockwave lithotripsy: Help or hindrance. *J Urol* 1989; **142**: 32–6

18 Morgentaler A, Bridge SS, Dretler SP. Management of impacted ureteral calculus. *J Urol* 1990; **143**: 263–6

19 Cass AS. Do upper ureteral stones need to be manipulated into the kidney before extracorporeal shock wave lithotripsy. *J Urol* 1992; **147**: 349–51

Authors

H.B. Joshi, MS, FRCS(Glas), Senior SHO (Stone Management Unit) in Urology, (currently Research Registrar in Urology, Southmead Hospital, Bristol).

O.O. Obadeyi, FRCS(Ed), Staff Urologist.

P.N. Rao, ChM, FRCS(Ed), Consultant Urologist.

Correspondence: Mr P.N. Rao, South Manchester University Hospital NHS Trust, Withington Hospital, Manchester M20 2LR, UK.

© 1999 *BJU International* **84**, 264–269
