# Supplementary material for: Efficacy of emergency extracorporeal shock wave lithotripsy in the treatment of ureteral stones: a meta-analysis
Source: BMC Urol. 2023 Apr 4;23:56. doi: 10.1186/s12894-023-01226-5 (PMC10074806; doi:10.1186/s12894-023-01226-5)
Supplement: Supplementary file 5 — Additional File 5: Kravchick 2005 [file 12894_2023_1226_MOESM5_ESM.pdf]

JOURNAL OF ENDOUROLOGY
Volume 19, Number 1, January/February 2005
© Mary Ann Liebert, Inc.

Emergency Extracorporeal Shockwave Lithotripsy for Acute Renal Colic Caused by Upper Urinary-Tract Stones

SERGEY KRAVCHICK, M.D.,[1] IGOR BUNKIN, M.D.,[1] EUGENY STEPNOV, M.D.,[1]
RONIT PELED, PH.D.,[2] LEONID AGULANSKY, M.D.,[1] and SHMUEL CYTRON, M.D.[1]

ABSTRACT

***Purpose*: To evaluate emergency SWL for the treatment of upper urinary-tract stones causing renal colic.**

***Patients and Methods*: Between January 1999 and June 2003, 53 patients with a mean age of 46.6 years (range 22–65 years) were enrolled. The inclusion criteria were acute renal colic, radiopaque 5-mm to 1.5-cm calculi in the ureteropelvic junction (N = 10) or upper ureter (N = 43), and no evidence of urinary-tract infection or acute renal failure. The mean stone size was 7.14 mm (range 5–13 mm). Patients were randomly assigned to the control (N = 28) and study (N = 25) groups using previously prepared cards in envelopes. Patients in the study group underwent emergency SWL, while patients in the control group underwent scheduled SWL within 30 days. Stone status was evaluated 4 weeks after lithotripsy. There was no significant difference between the control and study groups with respect to age, sex, stone location or volume, renal obstruction, or days spent in the hospital for pain control. Available fragments of stones were sent for infrared spectroscopy. Preoperative and postoperative data were compared in the two groups using SPSS 10.0 statistical software.**

***Results*: The SWL treatment lasted 50 ± 11 minutes. The stone-free rates were 72% and 64% and the efficiency quotients were 53% and 44% in study and control groups, respectively. Patients in the control group spent more time in the hospital ($P = 0.014$) and in recovery at home ($P = 0.011$).**

***Conclusion*: Emergency SWL for acute renal colic caused by upper-ureteral stones is a safe procedure and offers effective release from pain and obstruction. It also decreases hospitalization days and hastens return to normal activity.**

INTRODUCTION

THE MANAGEMENT OF ureteropelvic junction (UPJ) and upper-ureteral stones tends to be deferred. It is indisputable that in cases of infection and acute renal failure, prompt renal drainage followed by definitive treatment is a reasonable approach. In other instances, however, the treatment of stones depends mainly on the clinical symptoms and stone size and location.[1] The fact that most stones ≤4 may be eliminated spontaneously supports expectant management. However, SWL and ureteroscopy are sometimes required.[2,3]

The efficacy of SWL and its low morbidity rate make it a desirable option in the management of ureteral stones, even during acute renal colic and ureteral obstruction.[4–7] This approach may help to resolve the obstruction definitively and spare the patient from suffering. However, previous studies dedicated to emergency SWL were performed in a retrospective manner. In addition, they included stones of different sizes and locations: some of the relatively small calculi in the lower ureter could have passed without any treatment. Moreover, these studies did not compare the results of emergency and scheduled SWL.

We designed a prospective study that included only patients with stone sizes from 5 mm to 1.5 cm that were located in the upper urinary tract (UPJ or upper ureter). We compared the results of emergency and scheduled SWL and included in the analysis the work time lost by the patients during hospitalization, outpatient visits, and treatment procedures.

[1]Department of Urology, Barzilai Medical Center, Ashkelon, Israel.
[2]Epidemiology Research Institute, Ashkelon, Israel.

PATIENTS AND METHODS

Patients

Between January 1999 and June 2003, 53 consecutive patients (32 women, 21 men) with a mean age of 46.6 ± 11.1 years (range 22–65 years) were enrolled in this prospective study. All patients were selected according to the following criteria: acute renal colic, radiopaque 5-mm to 1.5-cm calculus in the UPJ (N = 10) or upper ureter (N = 43), and no evidence of urinary tract infection or acute renal failure. Patients with coagulation disorders and pregnant women were excluded. All patients underwent radiographic examination; in 15 cases, an intravenous pyelogram was performed, and in 38 cases, spiral CT was done. To detect ureteral obstruction in patients who underwent spiral CT, we performed nuclear imaging with ^{99m}Tc-MAG-3. The mean stone size was 7.14 (range 5-12 mm), and 22.6% of patients had calculi measuring ≥1 cm.

Patients were assigned to the control (N = 28) and study groups (N = 25) according to sequentially numbered sealed envelopes. Patients in the study group underwent SWL within 48 to 72 hours (emergency SWL); patients in the control group were discharged from the hospital after conservative treatment and scheduled for SWL within 30 days. In cases of intractable pain, an internal stent or nephrostomy tube was inserted and removed only after the definitive treatment. There was no significant difference between the control and study groups with respect to age, sex, or stone location or size or renal obstruction (Table 1).

Lithotripsy protocol

Stones were fragmented with an Econolith electrohydraulic lithotripter (focal-point depth 135–170 mm; focus area 60/13 mm length/width, spark voltage 15–22.5 kV) (Medispec Group, Israel). In every procedure, 3000 to 3500 shockwaves were delivered with an energy range of 18 to 23 kV. Stone fragmentation and residual fragments were evaluated 4 weeks after lithotripsy by plain abdominal films and ultrasonography. A stone-free result was defined as no evidence of calculi. When necessary, repeated SWL or ureteroscopy was performed. An efficiency quotient (EQ) was calculated for each group. Hospitalization was defined as the time from patient admission to our department until discharge + days of treatment (SWL, repeat SWL, auxiliary procedures).

When available, stone fragments were sent for infrared spectroscopy (IRS) to determine their composition. This new method requires only a few milligrams of sample.[8] According to the results of IRS, stones were defined as either compliant (hydroxyapatite, struvite, calcium oxalate dihydrate, uric acid) or resistant (cystine, brushite, calcium oxalate monohydrate) to SWL.[9,10]

Statistical methods

The results are expressed as the arithmetic mean ± SD. To compare patient and disease features such as age, stone size, days spent for pain control, hospitalization, outpatient visits, and days spent recovering at home in the two groups, we used the Student *t*-test. To assess the difference in categorical variables (sex, stone location, obstruction, stone-free rate, stone composition, repeat SWL and auxiliary procedures), the subset χ^2 test was used. For this purpose, we employed SPSS 10.0 statistical software, and $P < 0.05$ was considered significant.

RESULTS

In the study group, SWL was performed within 1 to 3 days (mean 2 ± 0.7 days; 76% within 48 hours). Patients in the control group were scheduled for SWL within 12 to 30 days (mean

TABLE 1. CLINICAL AND STATISTICAL DATA OF TWO PATIENT GROUPS

	Control Group	*Study group*	P *value*
Mean age (range)	47.92 ± 11.679 (22–63)	45.20 ± 10.607 (25–65)	0.393[a]
M/F (%)	44/56	36/64	0.564[b]
Mean stone size (mm) (range mm)	6.88 ± 2.421 (5–13)	7.40 ± 2.398 (5–12)	0.449[a]
Calculi ≥1 cm (%)	17.8	28	0.194[a]
Stone site (%)			
UPJ	12	28	0.157[b]
Upper ureter	88	72	
Obstruction (%)	24	20	0.733[b]
Stones resistant to SWL (%)	33	40	0.501[b]
Stone-free rate (%)	64	72	0.37[b]
Re-SWL	16	8	0.384[b]
Auxiliary procedure (%)	28	28	1.0[b]
EQ	44	53	
Mean days before SWL (range)	26 ± 5.85 (15–34)	2 ± 0.707 (1–3)	
Hospital days for pain control	4.84 ± 1.344 (3–7)	3.44 ± 1.635 (2–9)	0.0018
Hospitalization (days) (range)	7.72 ± 2.132 (5–13)	5.76 ± 3.218 (3–15)	0.014[a]
Outpatient visits (range)	1.6 ± 1.08 (0–4)	1.04 ± 1.064 (0–3)	0.042[a]

[a]Student's *t*-test.
[b]χ^2 test.

22 ± 5.7) after their departure from the hospital, but their treatment was actually delayed to 26 ± 5.85 days (range 15–34 days), including days spent in the hospital for pain reduction + the waiting list for SWL. Three patients in the control group passed calculi spontaneously before SWL. The SWL treatment lasted 50 ± 11 minutes. No patient in the study group needed any kind of drainage after SWL, while two patients from the control group were discharged from the hospital with a double-J stent and one with a nephrostomy tube.

An IRS analysis was performed in 49 of the cases (98%). Of the fragments, 59% showed homogeneous composition, most commonly calcium oxalate and phosphate, and 41% were binary mixtures composed of oxalates and phosphates or oxalates and urate. There was no significant difference between the two groups with respect to stone composition ($P = 0.501$).

The stone-free rates were 72% and 64% in the study and control groups, respectively, but this difference was insignificant ($P = 0.37$). The stone-free rates for calculi <1 cm were 94% and 75% in the study and control groups, respectively ($P = 0.098$). More patients in the control group required a second SWL (16% v 8%; $P = 0.38$). Equal numbers of auxiliary procedures were performed in the groups. The EQs were 53% and 44% in the study and control groups, respectively.

After emergency SWL, patients spent 0 to 6 days in the hospital (mean 1.4 ± 1.4). They spent significantly less time for pain control during initial hospitalization (hospitalization minus day of SWL) than patients in the control group ($P = 0.0018$). Patients in the control group spent significantly more days in the hospital, including initial hospitalization + additional hospitalization for pain control + hospitalization for auxiliary procedures (control group 7.7 ± 2.1 v study group 5.8 ± 3.2; $P = 0.014$). The control group also lost more workdays at home for pain control before or after SWL and auxiliary procedures (10.28 ± 4.49 *v* 7.04 ± 4.108; $P = 0.011$) and wasted more days for outpatient visits (1.6 ± 1.08 *v* 1.04 ± 0.789; $P = 0.042$).

DISCUSSION

Most ureteral stones measuring <5 mm in diameter are likely to be eliminated spontaneously. Although this clearance rate is lower for stones in the upper ureter,[11] expectant management of these calculi is common and reasonable. Therefore, we included in our study only patients with stones 5 mm to 1.5 cm in diameter. We also think that emergency SWL is a preferable treatment for all stones in the upper urinary tract. This view is supported by the results of the comprehensive study performed by Arrabal-Martin and colleagues,[12] who found that the complete success rate in the group of patients with obstructive lithiasis treated by *in situ* SWL was comparable to the results of ureteroscopy performed for calculi in the lumbar ureter. Those investigators concluded that the primary therapeutic option for lumbar ureterolithiasis is *in situ* SWL. In contrast, expectant treatment[11] or ureteroscopy[13] may be a reasonable choice for renal colic caused by calculi of the same size in the middle or lower ureter.

The EQ in our study (53%) was similar to that in a study dedicated to emergency SWL for stones ranging from 3 to 20 mm and located in the upper, middle, or lower ureter.[7] However, Doublet and associates[5] found a significant relation between stone location and stone-free rates after an emergency SWL session. Only 65% of their patients with upper urinary-tract stones became stone free. In contrast, Joshi et al[6] found that SWL was most successful for stones obstructing the upper ureter. Their results are similar to ours (72% stone-free rate).

The superior EQ and stone-free rate in our study group may be explained in the following way. There is dilation of the ureter and renal pelvis above the stone in most patients who undergo emergency SWL. This dilated area resembles an expansion chamber, which is important for better fragmentation during SWL.[14] Also, ureteral edema and fibrosis are less extensive in the early periods of stone impaction. These facts, as suggested by Joshi et al,[6] may improve the success rate in patients who undergo emergency SWL.

Ureteroscopy is one of the options that can simultaneously relieve the obstruction and extract stone fragments. The success rate for treating proximal-ureteral stones with small rigid and flexible ureteroscopes and the holmium laser ranges from 71.1%[15] to 90%.[16] When comparing the results of ureteroscopy and SWL for proximal-ureteral calculi ≥1 cm, the EQ was 0.76 for ureteroscopic lithotripsy and 0.43 for SWL (initial stone-free rate of 93% and 50%, respectively).[17] Alternately, the EQ was calculated as 0.81 for ureteroscopic lithotripsy and 0.72 for SWL in treating proximal-ureteral calculi <1 cm (initial stone-free rate 100% and 80%, respectively). For this reason, it was proposed that SWL should remain first-line therapy only for proximal-ureteral calculi <1 cm because of the lower morbidity and analgesic requirements.

Of the patients enrolled in the study, 22.6% had calculi ≥1 cm. This may explain the lower initial stone-free rate (72%/64%) and EQ (53%/44%) obtained in our study. The initial stone-free rate in the patients with calculi of <1 cm was 75% and 94% in the control and the study groups, respectively. Consequently, if we compare the results of SWL for proximal-ureteral calculi <1 cm, the results achieved in the control group are similar to the data of the above-mentioned study, and the results of the emergency SWL group are even more encouraging. However, the cost of SWL would have to decrease by more than $1489 to achieve cost equivalence with ureteroscopy.[16] All patients in our study group were discharged from the hospital without any kind of drainage, and only 8% of these patients required repeat SWL. They also spent less time in the hospital and promptly returned to work or normal activity. It must be emphasized that the stone-free rates for calculi of <1 cm were 94% in the study group. This result encouraged us to suggest that focusing emergency SWL on upper-tract stones ranging from 5 mm to <1 cm may decrease the cost of treatment (drainage procedure, hospitalization, repeat SWL, work time lost at home) and offer an economic advantage.

Several limitations of this study should be acknowledged. Only 53 patients were involved. A prospective trial with more patients will be required to substantiate our findings. Moreover, we investigated emergency SWL only for the upper urinary tract and only for radiopaque calculi ranging from 5 mm to 1.5 cm in greatest diameter. This highly selected group has an obvious quantitative limitation. Notwithstanding this shortcoming, our study compared the results of "emergency" and "scheduled" SWL in a prospective manner and suggested that the former is superior.

CONCLUSION

Emergency SWL of upper urinary-tract stones during acute renal colic is a safe procedure. It offers effective release from pain and obstruction. Moreover, it decreases days of hospitalization and hastens return to normal activity.

REFERENCES

1. Segura JW, Preminger GM, Assimos DG, et al. Ureteral Stones Clinical Guidelines Panel Summary Report on the Management of Ureteral Calculi. J Urol 1997;158:1915–1921.
2. Clayman RV. Outpatient treatment of middle and lower ureteric stones: Extra-corporeal shock wave lithotripsy versus ureteroscopic laser lithotripsy. J Urol 1999;162:1876–1877.
3. Turk MT, Jenkins AD. A comparison of ureteroscopy to in situ extracorporeal shock wave lithotripsy for the treatment of distal ureteral calculi. J Urol 1999;161:45–47.
4. Cass AS: In situ extracorporeal shock wave lithotripsy for obstructing ureteral stones with acute renal colic. J Urol 1992;148: 1786–1787.
5. Doublet JD, Tchala K, Tligui M, Ciofu C, Gattegno B, Thibault P. In situ extracorporeal shock wave lithotripsy for acute colic due to obstructing ureteral stones. Scand J Urol Nephrol 1997;31:137–139.
6. Joshi HB, Obadeyi OO, Rao PN. A comparative analysis of nephrostomy, JJ stent and urgent in situ extracorporeal shock wave lithotripsy for obstructing ureteric stones. Br J Urol 1999;84:264–269.
7. Tligui M, Khadime MR El, Tchala K, Haab F, Traxer O, Gattegno B, Thibault P. Emergency extracorporeal shock wave lithotripsy (ESWL) for obstructing ureteral stones. Eur Urol 2003;43:552–555.
8. Raichlin Y, Kravchick S, Cytron S, Gerber L, Katzir A. Fiberoptic infrared spectroscopy: A novel tool for the determination of the composition of urinary stones [abstract]. J Endourol 2003;17(suppl 1):22A.
9. Pittomvils G, Vandeursen H, Wevers M, et al. The influence of internal stone structure upon the fracture behavior of urinary calculi. Ultrasound Med Biol 1994;20:803–810.
10. Saw KC, Lingeman JE. Lesson 20: Management of calyceal stones. AUA Update Series 1999;20:154–159.
11. Coll DM, Varanelli MJ, Smith RC. Relationship of spontaneous passage of ureteral calculi to stone size and location as revealed by unenhanced helical CT. AJR Am J Roentgenol 2002;178:101–103.
12. Arrabal-Martin M, Pareja-Vilches M, Gutierrez-Tejero F, Mijan-Ortiz JL, Palao-Yago F, Zuluaga-Gomez A. Therapeutic options in lithiasis of the lumbar ureter. Eur Urol 2003;43:556–563.
13. Lifshitz DA, Lingeman JE. Ureteroscopy as a first-line intervention for ureteral calculi in pregnancy. J Endourol 2002;16:19–22.
14. Muller SC, Wilbert D, Thuroff J, Alken P. Extracorporeal shock wave lithotripsy of ureteral stones: Clinical experience and experimental findings. J Urol 1986;135:831–834.
15. Chow GK, Patterson DE, Blute ML, Segura JW. Ureteroscopy: Effect of technology and technique on clinical practice. J Urol 2003;170:99–102.
16. Bagley DH. Expanding role of ureteroscopy and laser lithotripsy for treatment of proximal ureteral and intrarenal calculi. Curr Opin Urol. 2002;12:277–280.
17. Lam JS, Greene TD, Gupta M. Treatment of proximal ureteral calculi: Holmium:YAG laser ureterolithotripsy versus extracorporeal shock wave lithotripsy. Urology 2002;167:1972–1976.
18. Lotan Y, Gettman MT, Roehrborn CG, Cadeddu JA, Pearle MS. Management of ureteral calculi: A cost comparison and decision making analysis. J Urol 2002:167:1621–1629.

Address reprint requests to:
Sergey Kravchick, M.D.
Haazmaut 87/35
Ashdod, Israel
