# Supplementary material for: Efficacy of emergency extracorporeal shock wave lithotripsy in the treatment of ureteral stones: a meta-analysis
Source: BMC Urol. 2023 Apr 4;23:56. doi: 10.1186/s12894-023-01226-5 (PMC10074806; doi:10.1186/s12894-023-01226-5)
Supplement: Supplementary file 6 — Additional File 6: Kumar 2010 [file 12894_2023_1226_MOESM6_ESM.pdf]

JOURNAL OF ENDOUROLOGY
Volume 24, Number 12, December 2010
© Mary Ann Liebert, Inc.
Pp. 2059–2066
DOI: 10.1089/end.2010.0066

A Prospective Randomized Comparison Between Early (<48 Hours of Onset of Colicky Pain) Versus Delayed Shockwave Lithotripsy for Symptomatic Upper Ureteral Calculi: A Single Center Experience

Anup Kumar, M.S., M.Ch., Nayan K. Mohanty, M.S., M.Ch., Manoj Jain, M.S.,
Sanjay Prakash, M.S., and Rajender P. Arora, M.S.

Abstract

Background and Purpose: The role of early/emergency shockwave lithotripsy (SWL) in symptomatic upper ureteral calculi has still not been established. We have performed a randomized comparison between early (<48 hours) *vs* delayed (>48 hours) SWL for symptomatic upper ureteral stones less than 1 cm to evaluate the feasibility, safety, and efficacy of early SWL in these patients.
Patients and Methods: One hundred and sixty consecutive patients with a single radiopaque upper ureteral stone <1 cm, who presented with an episode of colicky pain and who were undergoing treatment between July 2008 and June 2009 in our department were included. The patients were hospitalized and randomized into two groups—group A: SWL was performed within 48 hours of onset of colicky pain (early SWL) using the electromagnetic lithotripter (Dornier Alpha Compact) along with analgesics and hydration therapy; group B: SWL was performed after 48 hours (delayed SWL) along with analgesics and hydration therapy. The statistical analysis was performed in two groups regarding the patient demographic profile, presence of hydronephrosis, time to stone clearance, success rates, number of sessions needed, auxiliary procedures, modified efficiency quotient (EQ), and complications.
Results: Eighty patients were enrolled in each group. The mean stone size was 7.3 mm in group A *vs* 7.5 mm in group B ($P = 0.52$). The stone fragmentation rate was 88.75% in group A *vs* 91.2% in group B ($P = 0.35$). The overall 3-month stone-free rate was 86.3% (69/80) for group A *vs* 76.2% (61/80) for group B ($P = 0.34$). The mean time taken for stone clearance was significantly less in group A than in group B (10.2 days *vs* 21.1 days; $P = 0.01$). The number of sessions needed in group A were significantly less than in group B (1.3 *vs* 2.7; $P = 0.01$). The auxiliary procedure rate was also significantly lesser in group A than group B (16.3% *vs* 32.5%; $P = 0.001$). The modified EQ (in %) was 67.2 in group A *vs* 59.4 in group B ($P = 0.21$). The steinstrasse formation and requirement for percutaneous nephrostomy (PCN) were significantly less in group A (P:0.02 and P:0.01 respectively).
Conclusions: Early SWL (within 48 hours of onset of colicky pain) is feasible, safe, and highly efficacious in the management of symptomatic proximal ureteral stones <1 cm, resulting in a lesser requirement of number of SWL sessions, time taken for stone clearance, auxiliary procedure rate, and fewer complications in comparison with delayed SWL.

Introduction

With the introduction of shockwave lithotripsy (SWL) in the early 1980s as a management option for upper ureteral stones, it has become a primary treatment for most patients with uncomplicated upper urinary tract stones.[1–4] According to the European Association of Urology-American Urological Association (EAU-AUA) ureteral stones clinical guideline in 2007, 3-month stone-free rates for SWL in upper ureteral stones are approximately 90% and 68%, respectively, for upper ureteral stone size <1 cm and 1 to 2 cm.[5]

SWL has been one of the standard management options for upper ureteral stones <1 cm.[5] Ureteroscopy has been traditionally considered the surgical treatment of choice for patients with mid and lower ureteral stones.[6] With the advances made in ureteroscopic instruments and techniques, however, along with an increase in expertise, this modality has also become an established management option for upper ureteral

Department of Urology, Vardhman Mahaveer Medical College and Safdarjang Hospital, New Delhi, India.

stones up to 2 cm, as published in the 2007 guideline for the management of ureteral calculi.[5]

SWL can be used either as an *in-situ* or push-back technique or along with stent placement for upper ureteral stones.[2] SWL is a minimally invasive, simple, and safe treatment option for these patients.[4–7]

Ureteral stone is one of the most painful and prevalent urologic disorders. The usual management of acute renal colic from upper ureteral calculi is administration of analgesics and hydration therapy for relief of symptoms, except in complications such as acute pyelonephritis and acute renal failure.[8–14] The definitive management of stones in these patients is usually deferred because approximately 80% of stones <4 mm are expected to pass spontaneously.[10,15] As stone size increases, however, spontaneous passage becomes less likely.[10,15] Therefore, the ideal goal of management in these patients should be to get a fast complete stone clearance with minimal morbidity.[5,6,10,16]

Very few randomized studies have addressed the role of SWL in the treatment of these patients who present with renal colic.[17,18] The rationale for early use of SWL in these patients with acute renal colic is that ureteral mucosal edema starts developing after 24 to 48 hours, which might delay stone clearance after SWL.[19–21]

One can achieve maximal stone clearance with early SWL in the shortest possible time, with early detection of SWL failures necessitating auxiliary procedures.[19–21] Therefore, we have performed this randomized comparison between early (<48 hours of onset of renal colic) *vs* delayed (>48 hours) SWL for symptomatic upper ureteral stones <1 cm to evaluate the feasibility, safety, and efficacy of early SWL in these patients.

Patients and Methods

The study included 160 consecutive patients with a single radiopaque upper ureteral stone <1 cm, who presented with an episode of colicky pain and were undergoing treatment between July 2008 and June 2009 in our department. Exclusion criterion included bleeding disorders, active urinary infection, age >60 years and <15 years, weight >100 kg and <40 kg, comorbid cardiovascular and respiratory illnesses, fever >38°C, total leukocyte count >12,000/dL, serum creatinine level >1.5 mg/dL, solitary kidney, coexisting ureteral pathology including tumor/stricture, pregnancy, and severe hydronephrosis.

A total of 230 patients presented with acute colicky pain. The patients were hospitalized and underwent a detailed clinical evaluation, including familial and dietary history, baseline hematologic, biochemical tests, urine routine microscopy, urine culture sensitivity, 24-hour urine analysis, and ultrasonography of the kidneys, ureters, and bladder (KUB) region. Metabolic evaluation was performed in all patients after their first visit to the hospital, once they were enrolled in the study according to the EAU guideline on stones.[5] Noncontrast CT with three-dimensional (3-D) reconstruction was performed in all patients to assess stone location and size. Radiography of the KUB was performed in all patients to assess whether the stone was radioopaque.

Based on inclusion and exclusion criteria, 195 patients were eligible for the study. A detailed informed consent was given by 160 patients who were randomized into two groups using a computer-generated table based on randomized block design. Group A: SWL was performed within 48 hours of the onset of colicky pain (early SWL) using the electromagnetic lithotripter (Dornier Alpha Compact) along with intravenous (IV) fluids and intramuscular (IM) diclofenac. All patients initially received IV fluids and analgesics (diclofenac) 75 mg IM. The analgesic was repeated on demand, if pain persisted (diclofenac 75 mg IM q8h).

Group B: SWL was performed after 48 hours of onset of colicky pain (delayed SWL) along with IV fluids and IM diclofenac (repeated according to requirement). According to our routine protocol, all patients in both groups A and B received an occlusive dressing of eutectic mixture of lidocaine and prilocaine (EMLA) 5 gm, applied on a skin area of approximately 30 cm^2, corresponding to entry site of the shockwaves, 60 minutes before SWL along with oral tablet diclofenac sodium 60 minutes before SWL (dosage: 50 mg for body weight <70 kg, 100 mg for >70 kg).

The time interval between onset of colicky pain and start of SWL was recorded. According to the time interval between onset of colicky pain and onset of SWL treatment, the results in group A and B were further subanalyzed as: Group A1 < 24 hours and group A2 = 24 to 48 hours; group B1 = 48 to72 hours, group B2 = 72 to 96 hours; and Group B3 = 96 hours to 7 days. Figure 1 shows the CONSORT flow chart indicating the accrual, randomization, and patient inclusion/exclusion criteria of the study.

SWL was performed using the Dornier alpha-compact lithotripter at a shockwave rate of 100 Hz. A maximum of 3000 shockwaves were delivered during each session. The following parameters were recorded: Patient demographics—age, sex, body weight, height; stone characteristics—side, site, size, degree of hydronephrosis, previous stent placement; need for supplemental analgesia; treatment details—total number of shockwaves delivered, maximum voltage used during a session, and stone fragmentation rate.

Adverse effects of medications and skin lesions at the shockwave entry site were also noted. Supplemental analgesia in the form of a combination of IM pethidine (1 mg/kg body weight) and promethazine (0.5 mg/kg body weight, to counteract postnarcotic nausea) was administered, and the patients were monitored for respiratory depression, decrease in oxygen saturation, nausea, vomiting, drowsiness, and hypersensitivity reactions for 2 hours.

Stone fragmentation and clearance was assessed with radiography of the KUB region on the next day after each SWL session. Ultrasonography of the KUB region was performed to confirm stone clearance, once radiography did not reveal any residual fragments. Re-treatment SWL if needed (up to a maximum of four sessions) was performed after 24 hours, if any residual fragments were found. The patients remained in the hospital throughout the treatment in both groups. Noncontrast CT with 3-D reconstruction was performed at 3 months in all patients to assess stone-free status. The stone-free status at 3 months determined success or failure of the procedure. Post-SWL auxiliary procedures, including ureterorenoscopy with intracorporeal lithotripsy, percutaneous nephrolithotripsy, percutaneous nephrostomy (PCN), placement of a Double-J stent, were used within 48 hours in cases of failure of stone clearance, development of fever >100°F, steinstrasse, and worsening of pain. Stone analysis was performed in all cases. Treatment outcome was assessed by cal-

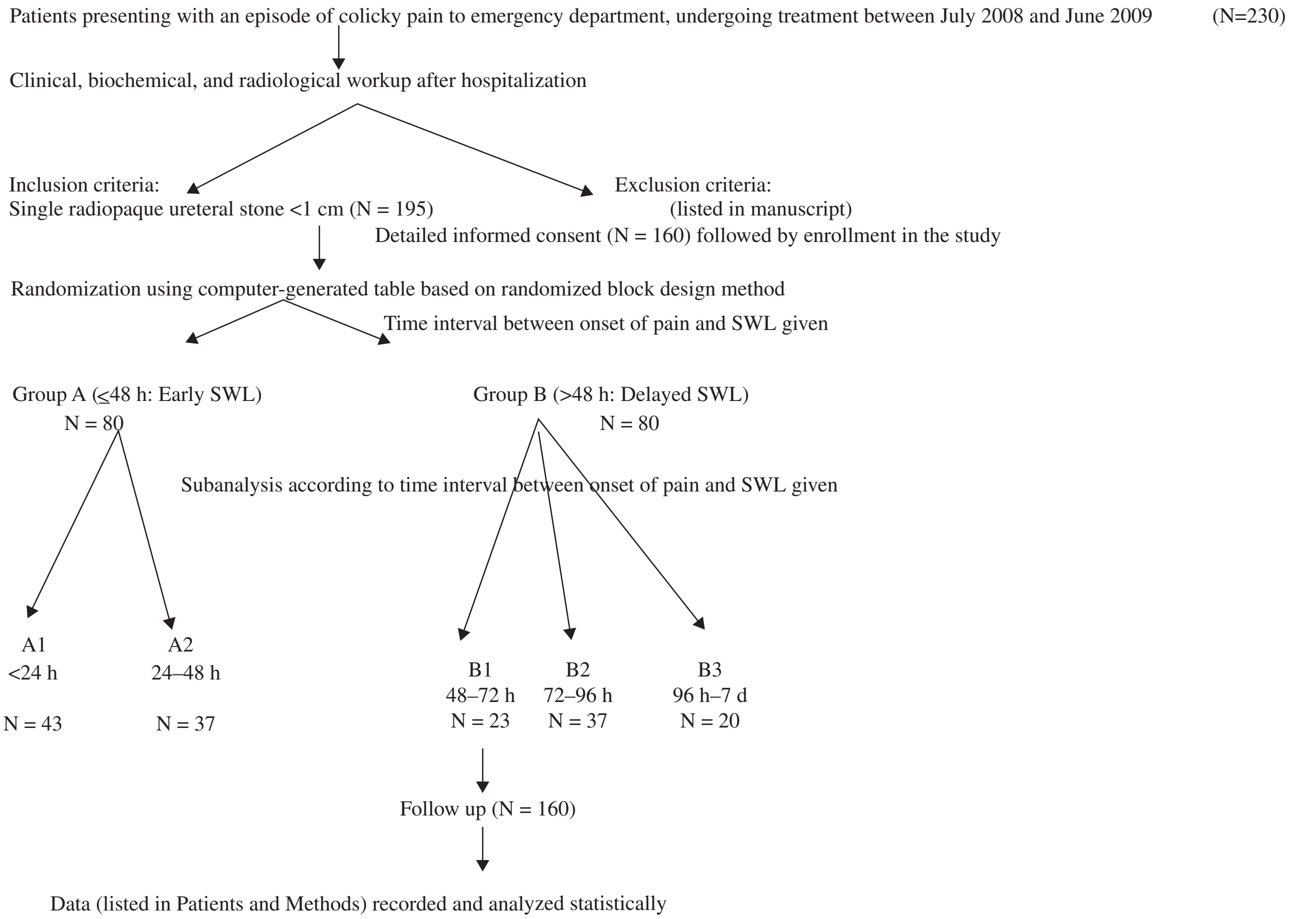

FIG. 1. CONSORT flowchart showing the accrual, randomization, and patient inclusion/exclusion criteria of the study. SWL = shockwave lithotripsy.

culating stone-free rate at 3 months, auxiliary procedure rate, and calculating the modified efficiency quotient (EQ).

Stone-free status was defined as no radiologic evidence of stone at 3 months or earlier, after initial SWL session, documented by noncontrast CT with 3-D reconstruction.

A modified EQ[3] was used to clearly distinguish between patients who became stone free because of SWL alone and those who were rendered stone free by auxiliary procedures. All patients were advised to use a strainer device during voiding after SWL sessions to collect passed stone fragments. Stone analysis was performed in all patients on these stone fragments using an infrared spectroscopy technique.

Statistical analysis was performed for groups A and B/subgroup A1 and A2 using the chi-square test for the categoric variables and Wilcoxon rank-sum test for nonparametric variables and the Student *t* test for continuous variables. Results between subgroups B1, B2, and B3 were analyzed statistically using the chi-square test for the categoric variables and one-way analysis of variance test for the continuous variables. The values of continuous variables were given as mean ± standard deviation, and all *P* values < 0.05 were taken as statistically significant.

Results

Patients' demographic profiles and stone characteristics are given in Table 1. A total of 160 patients were included in the study. Patients in the two groups had comparable patient demographics and stone characteristics (Table 1). Patients in the two groups had a similar metabolic profile; metabolic abnormalities were evident in 18 (22.5%) patients in group A and 19 (23.75%) patients in group B ($P = 0.23$). Renal hypercalciuria and hyperuricosuria were found in 12 and 6 patients in group A, respectively; 14 and 5 patients in group B, respectively.

Table 2 summarizes the treatment outcomes. The total number of shockwaves delivered, the maximum voltage given, and the SWL duration were comparable in two groups (Table 2). The requirement of supplemental analgesia was also comparable in both groups ($P = 0.65$). All patients who were declared stone free at 3 months showed no radiologic evidence of stone in noncontrast CT with 3-D reconstruction study. The stone fragmentation rate and stone-free rate at 3 months were slightly higher in group A compared with group B but were not statistically significant (92.5 *vs* 85%; $P = 0.2$ and 86.3 and 80%; $P = 0.31$, respectively). The mean time taken for stone clearance was significantly lower in group A compared with group B (10.2 *vs* 21.1 days, respectively, $P = 0.01$). On further subanalysis, the mean time taken for stone clearance was comparable in the AI and A2 subgroups; however, it was significantly higher in subgroup B3 compared with B1 and B2 (29.7 *vs* 14.9 *vs* 18.7 days in B3, B1, and B2, respectively; $P = 0.01$).

The re-treatment rate, the number of sessions of SWL needed, and the auxiliary procedure rate were significantly lower

Table 1. Patient Demographic Profiles and Stone Characteristics

	Group A			Group B				
	<24 h (A1)	24–48 h (A2)	Total	48–72 h (B1)	72–96 h (B2)	96 h–7 d (B3)	Total	P value[a]
Number	43	37	80	23	37	20	80	
Age (years)	37.6 ± 2.75	37.2 ± 3.26	37.4 ± 2.08	37.4 ± 3.7	37.5 ± 3.3	36.9 ± 1.3	37.3 ± 2.5	0.72 (NS)
Sex (M/F)	23/20	21/16	44/36	12/11	23/14	11/9	46/34	0.81 (NS)
BMI (kg/m2)	23.9	24.3	24.1	24.8	24.3	24.4	24.5	0.67 (NS)
Stone size (mm)	7.2 ± 1.63	7.4 ± 1.71	7.3 ± 1.5	7.3 ± 1.7	7.1 ± 1.4	8.1 ± 1.2	7.5 ± 1.7	0.43 (NS)
Stone location								
Right/left	19/24	18/19	37/43	11/12	21/16	12/8	47/33	0.72 (NS)
Hydronephrosis								
Nil	21	19	40	13	19	9	41	0.54 (NS)
Mild	12	11	23	7	11	7	25	0.61 (NS)
Moderate	10	7	17	3	7	4	14	0.73 (NS)
Previous Double-J stent placement	3	2	5	4	2	1	7	0.58 (NS)

[a]*P* value for groups A *vs* B.
BMI = body mass index; NS = not significant.

in group A (13.7 *vs* 26.3%, 1.3 *vs* 2.7 and 16.3 *vs* 32.5%, respectively, $P = 0.01$). On further subanalysis, the re-treatment rate and the number of sessions of SWL needed were comparable in the AI and A2 subgroups; however, it was significantly higher in subgroup B3 compared with B1 and B2 (35 *vs* 17.4 *vs* 27% and 3.8 *vs* 1.8 *vs* 2.8, respectively, in groups B3, B1, and B2; $P = 0.01$). The auxiliary procedure rate was higher in subgroup B3, however, compared with B1 and B2, although not significant statistically ($P = 0.53$). The curative auxiliary procedure rate was comparable in both groups ($P = 0.41$). The modified EQ was slightly higher in group A compared with group B; however, it was not significant statistically ($P = 0.21$).

The complications of SWL in different groups are summarized in Table 3. There were no skin lesions in either group from use of occlusive skin dressing of EMLA cream in all patients. The hematuria rate was comparable in all groups ($P = 0.54$). No significant adverse effects from diclofenac sodium or EMLA were seen in any of the patients. However, the steinstrasse and PCN requirement were significantly less in group A (6.25 *vs* 12.5%, $P = 0.02$, and 3.75 *vs* 7.5%, $P = 0.01$, respectively). On subgroup analysis, however, steinstrasse and PCN requirement were comparable in A1 and A2 ($P = 0.11$ and 0.65, respectively). Steinstrasse formation was significantly higher in subgroup B3 compared with B1 and B2 (20 *vs* 8.7 *vs* 10.8%, respectively; $P = 0.01$).

Table 4 summarizes the stone analysis profile performed in all 160 patients using an infrared spectroscopy technique. There was predominance of calcium oxalate monohydrate in all the groups (65% *vs* 70% in groups A and B, respectively, $P = 0.31$). Mixed stones were found in 2.5% and 3.75%, respectively, in groups A and B ($P = 0.41$). The mixed stone patterns included calcium oxalate monohydrate + calcium oxalate dihydrate, calcium oxalate + calcium phosphate, calcium oxalate + uric acid, and calcium oxalate + calcium magnesium phosphate).

Discussion

Since its introduction in 1980, shockwave lithotripsy has given a new dimension to management of urolithiasis, because of its simplicity, noninvasive nature, efficacy, and minimal morbidity. Further introduction of newer generation lithotripters, such as the Dornier alpha-compact with modifications in the aperture size of the shockwave source and consequent lower shockwave energy output, has made possible the use of less potent analgesia and sedation, with fewer complications, making it a day care procedure.[1–4,22]

The outcome of the management of ureteral stones depends on many factors such as location, stone size, composition, and degree of obstruction.[5,6] The majority of ureteral calculi smaller than 4 mm will pass spontaneously, and therefore need expectant management. Chances of spontaneous passage for ureteral calculi larger than 5 mm decrease significantly, however—48% for 7 to 9 mm and 25% for >9 mm. Spontaneous passage also depends on stone location with 48% for the upper ureter, 60% for the midureter, and 75% for the distal ureter.[5,6,10] Patients with upper ureteral stones primarily present with colicky pain but also with urinary tract infection, hematuria, and azotemia.

SWL has become a standard mode of treatment for ureteral calculi in the last two decades.[5,6] The AUA nephrolithiasis Guideline Panel in 1997 recommended SWL as an effective treatment of upper ureteral calculi with a 3-month stone-free rate of 83%.[6] This was further supported by guidelines for the management of ureteral calculi in 2007 by AUA education and research/EAU, which reported a 3-month stone-free rate of 82% in the proximal ureter,7 3% in the midureter, and 74% in the distal ureter.[5] With the availability of semirigid and flexible ureteroscopes, however, 74% stone-free rates for upper ureteral calculi and 90% to 100% for distal ureteral calculi have been reported recently.[6,7,23,24] The risk of significant complications has been reported as 4% and 11% for SWL and ureteroscopy, respectively.[5–7,23,24]

Traditionally, a patient who presented with colicky pain from upper ureteral calculi has been treated with injectable analgesics and IV fluids, if necessary. Pain relief still remains the most urgent step in patients with an acute stone episode. Oral diclofenac (nonsteroidal anti-inflammatory drug) in the prophylaxis of recurrent renal colic was evaluated in a double-blind placebo-controlled prospective study by Laerum and associates.[12] They demonstrated that this treatment option was effective in reducing colic and hospital admissions,

Table 2. Treatment Outcomes

	Group A			Group B				
	<24 h (A1)	*24–48 h (A2)*	*Total*	*48–72 h (B1)*	*72–96 h (B2)*	*96 h–7 d (B3)*	Total	P *value*[a]
Number	43	37	80	23	37	20	80	
SWL duration (min)	40.3 ± 1.25	39.23 ± 2.27	39.7 ± 2.4	41.2 ± 1.7	38.5 ± 2.5	40.2 ± 1.6	39.9 ± 3.1	0.67 (NS)
No. of shocks delivered	2854.21 ± 137.2	2732.75 ± 121.1	2793.48 ± 141.4	2813.54 ± 163.2	2765.43 ± 157.3	2737.67 ± 132.4	2772.21 ± 115.3	0.43 (NS)
Voltage (Kv)	12.1 ± 0.33	12.2 ± 0.41	12.15 ± 0.37	12.4 ± 0.23	12.3 ± 0.33	12.2 ± 0.25	12.45 ± 2.5	0.58 (NS)
Supplemental analgesia needed (%)	2	1	3 (3.75)	2	1	1	4 (5)	0.65 (NS)
Stone fragmentation (%)	90.7 (39/43)	94.6 (35/37)	92.5 (74/80) $P^b = 0.34$ (NS)	86.9 (20/23)	83.7 (31/38)	85 (17/20)	85 (68/80) $P^b = 0.42$ (NS)	0.2 (NS)
Stone-free rate at 3 months (%)	86.04 (37/43)	86.4 (32/37)	86.3 (69/80) $P^b = 0.25$ (NS)	82.3 (19/23)	78.4 (29/37)	80 (16/20)	80 (64/80) $P^b = 0.27$ (NS)	0.31 (NS)
Time taken for stone clearance (d)	10.3	10.1	10.2 $P^b = 0.45$ (NS)	14.9	18.7	29.7	21.1 $P^b = 0.01$ (S)	0.01 (S)
Re-treatment rate (%)	13.9 (6/43)	13.5 (5/37)	13.7 (11/80) $P^b = 0.32$ (NS)	17.4 (4/23)	27 (10/37)	35 (7/20)	26.3 (21/80) $P^b = 0.01$ (S)	0.01 (S)
No. of sessions of SWL	1.4	1.2	1.3 $P^b = 0.37$ (NS)	1.8	2.5	3.8	2.7 $P^b = 0.01$ (S)	0.01 (S)
Post-SWL auxiliary procedure rate(%)	16.27 (7/43)	16.21 (6/37)	16.3 (13/80) $P^b = 0.41$ (NS)	17.4 (4/23)	35.1 (13/37)	45 (9/20)	32.5 (26/80) $P^b = 0.53$ (NS)	0.01 (S)
Curative auxiliary procedure rate at 3 months (%)	6.9 (3/43)	5.4 (2/37)	6.25 (5/80) $P^b = 0.23$ (NS)	8.7 (2/23)	8.1 (3/37)	10 (2/20)	8.75 (7/80) $P^b = 0.32$ (NS)	0.41 (NS)
Modified EQ (%)	66.1	68.3	67.2 $P^b = 0.33$ (NS)	62.4	58.3	57.5	59.4 $P^b = 0.55$ (NS)	0.21 (NS)

[a]*P* value for groups A and B.
[b]*P* value for subgroups A1 *vs* /A2 and B1 *vs* B2 *vs* B3.
SWL = shockwave lithotripsy; NS = not significant; S = significant; EQ = efficiency quotient.

Table 3. Complications

	Group A			Group B				
	< 24 h (A1)	*24–48 h (A2)*	*Total*	*48–72 h (B1)*	*72–96 h (B2)*	*96 h–7 d (B3)*	*Total*	P *value*[a]
Number	43	37	80	23	37	20	80	
Steinstrasse (%)	6.9 (3/43)	5.4 (2/37)	6.25 (5/80) $P^b = 0.11$ (NS)	8.7 (2/23)	10.8 (4/37)	20 (4/20)	12.5 (10/80) $P^b = 0.01$ (S)	0.02 (S)
Hematuria (%)	39.5 (17/43)	37.8 (14/37)	38.7 (31/80) $P^b = 0.32$ (NS)	39.1 (9/23)	43.24 (16/37)	45 (9/20)	42.5 (34/80) $P^b = 0.63$(NS)	0.54 (NS)
PCN (%)	4.65 (2/23)	2.7 (1/23)	3.75 (3/80) $P^b = 0.65$(NS)	4.34 (1/23)	5.4 (2/37)	15 (3/20)	7.5 (6/80) $P^b = 0.11$ (NS)	0.01 (S)

[a]*P* value for groups A and B.
[b]*P* value for subgroups A1 *vs* /A2 and B1 *vs* B2 *vs* B3.
S = significant; NS = not significant; PCN = percutaneous nephrostomy.

even if stone passage rate was not affected. Tramadol is more potent than previous oral preparations, with fewer opioid-type side effects and less potential for dependence.[13] Ketorolac given intramuscularly is as effective as tramadol with an earlier analgesic effect.[14]

Medical expulsive therapy has been described to facilitate and accelerate the spontaneous passage of ureteral stones as well as stone fragments generated with SWL.[25–27] These findings indicate that alpha blockers (eg, tamsulosin) facilitate ureteral stone passage while nifedipine may provide a marginal benefit. Most of these studies have been performed on distal ureteral stones, however. [25–27] Our study included only upper ureteral stones; therefore, this therapy was not used before SWL.

Definitive management, either SWL or ureteroscopy, is usually deferred for a later stage.[8–11] Deferring the definitive treatment, however, usually results in more episodes of colicky pain along with other complications, such as urinary tract infection, azotemia, thus increasing morbidity. Very few studies, however, have reported the role of early/emergency SWL in the management of upper ureteral calculi immediately after colicky pain.[16–18,28–31] Among these, only two prospective studies have addressed this issue.[17,18] SWL is a good option for these patients because of its noninvasiveness, low overall morbidity, with no need for anesthesia. The pathophysiology that supports early use of SWL in these patients is that ureteral edema caused by an impacted symptomatic calculus develops over time, which impairs stone clearance after SWL. This gradual ureteral edema develops after 24 to 48 hours, as proved in a few published series. The stone bed mucosa has shown a hyperplastic appearance with increased mitosis, which develops after 48 hours, on histopathologic examination, further supporting the above theory. This ureteral edema also prevents formation of the expansion chamber and liquid interface, thus also reducing fragmentation rates after SWL.[19–21] Thus, it seems logical to use SWL early in these patients within 48 hours, before development of ureteral edema.

Very few published series, however, have addressed the role of early SWL in patients who present with colicky pain from upper ureteral calculi. The main goal of treatment in these patients should be achieving faster pain relief and early removal of stones with minimal morbidity.[5,6,10,16] Criteria for deciding what is early SWL have not been addressed properly, however. Only two published prospective trials have addressed this. Tombal and colleagues[18] compared medical therapy alone with medical therapy and SWL in patients with symptomatic ureteral stones. SWL was performed within 6 hours of onset of pain. They concluded that early SWL is a valuable therapeutic option in proximal ureteral stones, resulting in increased stone clearance and shorter hospital stay. Seitz and coworkers[17] have prospectively compared the role of piezoelectric SWL for a solitary proximal upper ureteral stone in patients who present with colic and noncolic patients. SWL was performed within 24 hours of onset of pain. They concluded that early/rapid SWL should be performed in

Table 4. Stone Analysis Profile of Patients

	Group A			Group B				
Stone composition (%)	*< 24 h (A1)*	*24–48 h (A2)*	*Total*	*48–72 h (B1)*	*72–96 h (B2)*	*96 h–7 d (B3)*	*Total*	P *value*[a]
Calcium oxalate	28 (65.1)	24 (64.8)	52 (65)	15 (65.2)	27 (72.9)	14 (70)	56 (70)	0.31 (NS)
Monohydrate	23 (53.5)	19 (51.3)	42 (52.5)	12 (52.2)	21 (56.7)	11 (55)	44 (55)	0.11 (NS)
Dihydrate	5 (11.6)	5 (13.5)	10 (12.5)	3 (13)	6 (16.2)	3 (15)	12 (15)	0.32 (NS)
Calcium carbonate	6 (13.9)	5 (13.5)	11 (13.8)	3 (13)	4 (10.8)	2 (10)	9 (11.3)	0.41 (NS)
Calcium phosphate	7 (16.3)	4 (10.8)	11 (13.8)	4 (17.3)	2 (5.4)	2 (10)	8 (10)	0.25 (NS)
Cystine	2 (4.6)	2 (5.4)	4 (5)	1 (4.3)	2 (5.4)	1 (5)	4 (5)	0.36 (NS)
Mixed	1 (2.3)	1 (2.7)	2 (2.5)	1 (4.34)	1 (2.7)	1 (5)	3 (3.75)	0.41 (NS)

[a]*P* value for groups A *vs* B.
NS = not significant.

proximal symptomatic ureteral stones because significant impaction of stone does not form within 24 hours. The time to stone clearance and treatment outcome in both groups were comparable and independent of coexisting hydronephrosis.[17] Tligui and colleagues[28] evaluated the role of early/emergency SWL in patients with symptomatic ureteral calculi and found this to have a satisfactory success rate with low morbidity. They did not mention time interval between onset of pain and start of SWL, however. Kravchick and associates[31] also reported the satisfactory role of early SWL (within 2 days) in patients with symptomatic ureteral calculi.

Our study is a prospective randomized trial in which we considered early SWL as within 48 hours, as supported by the pathophysiology of symptomatic upper ureteral stones and published series. We have taken only upper ureteral radiopaque stones <1 cm, because SWL results are best for upper ureteral stones <1 cm.[5,6] We have subanalyzed the two groups according to time interval between onset of pain and start of SWL. Our results have shown that as this mean colic to SWL time increases beyond 48 hours, mean days taken for stone clearance significantly increase (10.2 *vs* 21.1 days, respectively, in groups A and B), and this difference can also be appreciated in subgroups B1, B2, and B3. Similiarly, the retreatment rate, number of SWL sessions needed, and auxiliary procedure rates significantly increase after 48 hours, and this difference can also be appreciated in subgroups B1, B2, and B3. The stone fragmentation rate also decreases after 48 hours; however, the difference is not statistically significant, thus supporting the pathophysiology theory. Similiarly, the stone-free rate at 3 months and modified EQ decrease after 48 hours, but could not attain statistical significance. More interestingly, steinstrasse formation and the need for PCN significantly increase after 48 hours.

All these findings can be explained on the basis of pathophysiology described above supporting early SWL. Thus, development of ureteral edema after 24 hours along with mucosal hyperplasia prevents formation of expansion chamber and liquid interface. This not only reduces fragmentation rates after SWL, but also impairs stone clearance after SWL, resulting in increased need for re-treatment, number of SWL sessions needed, and complications in the delayed SWL group. The stone-free rate at 3 months of 86.3% and modified EQ of 67.2 are comparable with published series on early as well as deferred SWL.[16–18,28–31] Moreover, complications reported are also comparable with published series on both early as well as deferred SWL.[16,18,28–31]

All the patients tolerated SWL treatment well. Thus, we have achieved the goals of early pain relief and early clearance of ureteral stones with minimal morbidity in these patients.

Conclusions

Early SWL (within 48 hours of onset of colicky pain) is feasible, safe, and highly efficacious in the management of symptomatic proximal ureteral stones <1 cm, resulting in a lesser requirement of SWL sessions, time taken for stone clearance, the auxiliary procedure rate, and fewer complications in comparison with delayed SWL.

Disclosure Statement

No competing financial interests exist.

References

1. Chaussy CG, Brendel W, Schmiedt E. Extracorporeally induced destruction of kidney stones by shock waves. Lancet 1980;2:1265–1268.
2. Chow GK, Streem SB. Extracorporeal lithotripsy. Update on technology. Urol Clin North Am 2000;27:315–322.
3. Chaussy C, Schmiedt E, Jocham D. First clinical experience with extracorporeally induced destruction of kidney stones by shock waves. J Urol 1982;127:417–420.
4. Anderson KR, Keetch DW, Albala DM, et al. Optimal therapy for the distal ureteral stone: Extracorporeal shock wave lithotripsy versus ureteroscopy. J Urol 1994;152:62–65.
5. Preminger GM, Tiselius HG, Assimos DG, et al. 2007 Guideline for the management of ureteral calculi. Eur Urol 2007;52:1610–1631.
6. Segura JW, Preminger GM, Assimos DG, et al. Ureteral stones Clinical Guidelines Panel summary report on the management of ureteral calculi. J Urol 1997;158:1915–1921.
7. Wu CF, Shee JJ, Lin WY, et al. Comparison between extracorporeal shock wave lithotripsy and semirigid ureterorenoscope with holmium:YAG laser lithotripsy for treating large proximal ureteral stones. J Urol 2004;172:1899–1902.
8. Teichman JM. Clinical practice. Acute renal colic from ureteral calculus. N Engl J Med 2004;350:684–693.
9. Cooper JT, Stack GM, Cooper TP. Intensive medical management of ureteral calculi. Urology 2000;56:575–578.
10. Francesca F, Bader P, Echtle D, et al. EAU guidelines on pain management. Eur Urol 2003;44:383–389.
11. Worster A, Richards C. Fluids and diuretics for acute ureteric colic. Cochrane Database Syst Rev 2005;3:CD004926.
12. Laerum E, Ommundsen OE, Gronseth JE, et al. Oral diclofenac in the prophylatic treatment of recurrent renal colic: A double-blind comparison with placebo. Eur Urol 1995;28: 108–111.
13. Eray O, Cete Y, Oktay C, Karsli B, Akça S, Cete N, et al. Intravenous single dose tramadol versus meperidine for pain relief in renal colic. Eur J Anaesthesiol 2002;19:368–70.
14. Nicolás Torralba JA, Rigabert Montiel M, Banon Pérez V, et al. [Intramuscular ketorolac compared to subcutaneous tramadol in the initial emergency treatment of renal colic.] (Spa) Arch Esp Urol 1999;52:435–437.
15. Cummings JM, Boullier JA, Izenberg SD, et al. Prediction of spontaneous ureteral calculus passage by an artificial neural network. J Urol 2000;164:326–328.
16. Dasgupta R, Hegarty N, Thomas K. Emergency shock wave lithotripsy for ureteric stones. Curr Opin Urol 2009;19:196–199.
17. Seitz C, Tanovic E, Kikic Z, et al. Rapid extracorporeal shock wave lithotripsy for proximal ureteral calculi in colic versus noncolic patients. Eur Urol 2007;52:1223–1227.
18. Tombal B, Mawlawi H, Feyaerts A, et al. Prospective randomized evaluation of emergency extracorporeal shock wave lithotripsy (ESWL) on the short-time outcome of symptomatic ureteral stones. Eur Urol 2005;47:855–859.
19. Deliveliotis C, Chrisofos M, Albanis S, et al. Management and follow-up of impacted ureteral stones. Urol Int 2003;70: 269–272.
20. Mueller SC, Wilbert D, Thueroff JW, Alken P. Extracorporeal shock wave lithotripsy of ureteral stones: Clinical experience and experimental findings. J Urol 1986;135:831–834.
21. Kim HL, Labay PC, Boyarsky S, Glenn JF. An experimental model of ureteral colic. J Urol 1970;104:390–394.
22. Nomikos MS, Sowter SJ, Tolley DA. Outcomes using a fourth-generation lithotripter: A new benchmark for comparison? BJU Int 2007;100:1356–1360.

23. Stewart GD, Bariol SV, Moussa SA, et al. Matched pair analysis of ureteroscopy vs. shock wave lithotripsy for the treatment of upper ureteric calculi. Int J Clin Pract 2007;61: 784–788.
24. Youssef RF, El-Nahas AR, El-Assmy AM, et al. Shock wave lithotripsy versus semirigid ureteroscopy for proximal ureteral calculi (<20 mm): A comparative matched-pair study. Urology 2009;73:1184–1187.
25. Resim S, Ekerbicer HC, Ciftci A. Role of tamsulosin in treatment of patients with steinstrasse developing after extracorporeal shock wave lithotripsy. Urology 2005;66:945–948.
26. Porpiglia F, Destefanis P, Fiori C, Fontana D. Effectiveness of nifedipine and deflazacort in the management of distal ureter stones. Urology 2000;56:579–582.
27. Dellabella M, Milanese G, Muzzonigro G. Randomized trial of the efficacy of tamsulosin, nifedipine and phloroglucinol in medical expulsive therapy for distal ureteral calculi. J Urol 2005;174:167–172.
28. Tligui M, El Khadime MR, Tchala K, et al. Emergency extracorporeal shock wave lithotripsy (ESWL) for obstructing ureteral stones. Eur Urol 2003;43:552–555.
29. Cass AS. In situ extracorporeal shock wave lithotripsy for obstructing ureteral stones with acute renal colic. J Urol 1992;148:1786–1787.
30. Joshi HB, Obadeyi OO, Rao PN. A comparative analysis of nephrostomy, JJ stent and urgent in situ extracorporeal shock wave lithotripsy for obstructing ureteric stones. BJU Int 1999;84:264–269.
31. Kravchick S, Bunkin I, Stepnov E, et al. Emergency extracorporeal shockwave lithotripsy for acute renal colic caused by upper urinary-tract stones. J Endourol 2005;19:1–4.

Address correspondence to:
Anup Kumar, M.S., M.Ch.
Department of Urology
VMMC and Safdarjang Hospital
New Delhi
India

E-mail: anup_14k@yahoo.com

Abbreviations Used

AUA = American Urological Association
CT = computed tomography
EAU = European Association of Urology
EMLA = eutectic mixture of lidocaine and prilocaine
EQ = efficiency quotient
IM = intramuscular
IV = intravenous
KUB = kidneys, ureters, and bladder
PCN = percutaneous nephrostomy
SWL = shockwave lithotripsy
3-D = three-dimensional
