# Supplementary material for: Efficacy of emergency extracorporeal shock wave lithotripsy in the treatment of ureteral stones: a meta-analysis
Source: BMC Urol. 2023 Apr 4;23:56. doi: 10.1186/s12894-023-01226-5 (PMC10074806; doi:10.1186/s12894-023-01226-5)
Supplement: Supplementary file 7 — Additional File 7: Seitz 2005 [file 12894_2023_1226_MOESM7_ESM.pdf]

available at www.sciencedirect.com
journal homepage: www.europeanurology.com

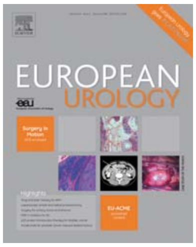

European Association of Urology

Stone Disease

Rapid Extracorporeal Shock Wave Lithotripsy Treatment after a First Colic Episode Correlates with Accelerated Ureteral Stone Clearance

Christian Seitz *, *Harun Fajković, Mesut Remzi, Matthias Waldert, Mehmet Özsoy, Gero Kramer, Michael Marberger*

Department of Urology, Medical University of Vienna, Währinger Gürtel 18–20, 1090 Vienna, Austria

Article info

Article history:
Accepted December 1, 2005
Published online ahead of print on December 28, 2005

Keywords:
ESWL
Proximal ureter
Colic
Stone clearance

Abstract

Objectives: To investigate the relationship between delay in extracorporeal shock wave lithotripsy (ESWL) after a first colic and subsequent time to complete stone clearance.
Methods: This prospective, non-randomized study included 94 patients treated with ESWL for unilateral solitary proximal ureteral stones after at least one episode of colic pain. Time between the first onset of colic pain and ESWL and stone clearance was recorded. The pretherapeutic degree of hydronephrosis has been assessed using ultrasound.
Results: Mean stone size was 7.9 ± 2.3 mm and mean time before ESWL after a first colic was 93.4 ± 143.5 h. At 3 months, 3 patients were lost to follow-up. In 76.9% of patients stones were completely cleared and a further 3.3% harbored residual fragments ≤3 mm. Delay in treatment after a first colic correlated with subsequent time to stone clearance ($p < 0.0001$). Mean time to stone clearance in patients treated within 24 h was 6.4 ± 6.3 days compared with 16.0 ± 17.8days for those treated later ($p = 0.008$). Maximum stone diameter correlated with time to stone clearance ($p = 0.031$), but the degree of hydronephrosis did not.
Conclusions: Rapid ESWL after a first onset of colic pain resulted in accelerated stone clearance independent of the degree of hydronephrosis but had no impact on the need for auxiliary procedures.
© 2005 Elsevier B.V. All rights reserved.

* Corresponding author. Medical University of Vienna, Währinger Gürtel 18–20, 1090 Vienna, Austria. Tel.: +43 1 40 400 2616; fax: +43 1 408 9966.
E-mail address: drseitz@gmx.at (C. Seitz).

1. Introduction

Since its introduction in the early 1980s [1] extracorporeal shock wave lithotripsy (ESWL) has become an effective treatment for urinary tract stones. The goal of the management of ureteral calculi is to achieve fast, complete stone clearance with minimal morbidity. Factors affecting stone clearance

0302-2838/$ – see back matter © 2005 Elsevier B.V. All rights reserved. doi:10.1016/j.eururo.2005.12.003

should be considered when choosing a treatment modality. The influence of symptoms, stone size, degree and duration of obstruction on time to stone clearance and treatment success with ESWL remains controversial. Impacted stones tend to be more resistant to ESWL [2]. In vitro studies suggest that this may be due to lack of a liquid interface surrounding the impacted stone. Confinement of a model stone was shown to be associated with substantial reduction in fragmentation [3].

The rationale for use of rapid ESWL after a renal colic episode is to attain maximum stone clearance in the shortest possible time with early detection of lithotripsy failures that could be treated with auxiliary procedures. Ureteral edema formation over time causing impaction might delay stone clearance after ESWL [3]. We investigated the relationship between rapid ESWL after a first colic episode and subsequent time to complete stone clearance, assuming that early treatment leads to accelerated stone clearance.

2. Material and methods

A total of 94 patients with a solitary proximal ureteral stone, at least one episode of acute renal colic, and no previous **active** treatment were prospectively, **non-randomly** enrolled in the study between March 2003 and January 2005. After presentation each patient was scheduled for ESWL as soon as possible. Variable time intervals between a first colic episode and referral to our clinic led to a variance in treatment delay.

Stones were detected by either non-contrast computerized tomography when symptomatic or by excretory urography in the asymptomatic interval. The presence of hydronephrosis was defined by ultrasound immediately before ESWL treatment. Patients were divided into four groups according to the degree of stone induced hydronephrosis. Group 0 had no urinary system dilatation, group 1 had mild dilatation of the renal pelvis, group 2 had a moderate dilatation and group 3 a severe dilatation of the renal pelvis and calices.

Pregnant women and patients with ureteric strictures, clotting disorders, or a solitary non-functioning kidney were excluded. Laboratory investigations included urinalysis, urinary culture, serum creatinine determination, and a coagulation profile. All patients were afebrile, but urinary cultures were positive in 5 individuals (5.3%) who received appropriate antimicrobial drugs prior to ESWL.

Patients were treated with a Piezolith 3000 (Richard Wolf, Knittlingen, Germany) with dual ultrasound/fluoroscopic detection. Initial ESWL was performed as an inpatient procedure for administrative reasons, whereas retreatments were usually performed in an outpatient setting. Proximal ureteral stones were fragmented with the patient in the supine position. Stone size was measured using excretory urography. The maximum stone diameter was 7.9 ± 2.3 mm (range 3.0 to 15.0 mm), with a transverse diameter of 5.5 ± 1.8 mm (range 3.0 to 12.0 mm). Patients did not receive pain medication routinely during ESWL. Routine pain control was intravenous metamizol, 2.5g, on demand. The time between the first onset of colic pain and ESWL treatment and the time between ESWL and stone clearance was recorded.

Patients in whom ESWL failed to disintegrate the stone completely during the first treatment underwent repeat sessions. Those, in whom ESWL had no impact on the stone during the first session, as evidenced by plain radiography (KUB), underwent ureterorenoscopy (URS). Stone-free status was established by urinary tract plain X-ray and intravenous urography as well as by native computerized tomography in cases where residual fragments could not be excluded. Residual fragments were defined as those remaining in the kidney or ureter 3 months after the last ESWL session. The presence of fragments >3 mm in diameter was considered indicative of treatment failure. Patients with stones ≤ 3 mm in diameter were included in neither the treatment failure nor the stone-free group.

Patients were reviewed 1 day after each ESWL session with KUB and renal ultrasound to assess stone fragmentation and hydronephrosis. Repeat treatment was carried out immediately when incomplete fragmentation was observed. Incomplete fragmentation after four sessions was considered ESWL failure. Follow-up continued for a maximum of 3 months using KUB and renal sonography every 2 weeks or after each stone passage until complete clearance was achieved. Stone analysis to determine the crystalline structure was carried out using X-ray diffraction.

JMP version 3.2.2 1989–97 software (SAS Institute, Inc.) was used for statistical analysis. Wilcoxon and Pearson correlations were used for comparison between continuous variables and linear regression. A multiple regression model was used for factors maintaining a statistically significant impact on the time to stone clearance, indicating that they act independently.

3. Results

Mean patient age was 45.9 years (range 24 to 81 years). At 3 months, 3 patients were lost to follow-up. Of the remaining 91, 70 (76.9%) were free of stones, 3 (3.3%) harboured residual fragments ≤ 3 mm in diameter in the lower calyx, 16 (17.6%) underwent URS, and 2 (2.2%) underwent percutaneous nephrolitholapaxy (PNL). The indication to perform an URS was based on recurrent colic pain and/or persistence of fragments >3 mm after repeated ESWL sessions.

During ESWL all stones were focused with fluoroscopy and/or ultrasonography only and opacification of the excretory route was never required. All stones were located in the proximal ureter, radiographically defined as between the ureteropelvic junction and the pelvic brim. Mean time to ESWL after a first colic episode was 93.4 ± 143.5 h (range 4.0 to 840.0 h). The number of shocks per stone delivered in several sessions was 8782 ± 4844 (range 2500 to 25,000), with a mean intensity of 18.3 ± 1.7 (range 12.00 to 20.00). 48.9% of patients required one

session only, 30.2% a second, 16.6 % a third, and 4.3% a fourth for complete stone fragmentation. The mean number of sessions performed per patient was 1.8 (range 1 to 4), and correlated significantly with the time to ESWL after a first colic episode ($p = 0.037$). Patients treated within 24 h after their first colic needed significant less ESWL sessions than patients treated after 96 h ($p = 0.014$). A positive correlation between the number of sessions and a delayed time to stone clearance was also observed ($p = 0.003$).

Immediately before ESWL treatment hydronephrosis was present in 85.7% of patients. Group 0 (14.3%, $n = 13$) had no urinary system dilatation, group 1 (30.8%, $n = 28$) had a mild dilatation of the renal pelvis, group 2 (49.5%, $n = 45$) had a moderate dilatation and group 3 (5.5%, $n = 5$) a severe dilatation of the renal pelvis and calices. The presence of hydronephrosis detected by ultrasound had no impact on the time to stone clearance or on treatment success ($p = 0.14$, $p = 0.25$). When stratifying patients for different degrees of hydronephrosis (1–3) again no significant correlation was observed ($p = 0.43$, $p = 0.59$).

The overall mean time to stone clearance was 14.2 ± 16.6 days (range 0.5 to 90.0 days). There was a significant correlation between rapid ESWL treatment after the first onset of colic pain and the subsequent time to complete stone clearance ($p < 0.001$). This effect was most pronounced within the first 24 h after a first colic episode, with a mean time to stone clearance of 6.4 ± 6,3 days compared with 16.0 ± 17.8 days for treatment after this time ($p = 0.008$). A further delay of treatment further increased the mean time to stone clearance. Patients treated after 96 h presented with a mean time to stone clearance of 22.2 ± 23.5 days (Table 1). Additionally, there was no significant difference between groups in terms of patient age, gender or stone composition.

In patients with small ureteric calculi it may be scientifically questionable to credit stone clearance within several weeks after ESWL to the treatment, as many stones would have passed spontaneously. To avoid this ambiguity we investigated a subgroup of 78 patients with stones ≥6 mm in diameter and thus unlikely to pass spontaneously [4–7]. Again a significant ($p < 0.001$) correlation was documented between delay in treatment after a first colic episode and the time at which complete stone clearance was observed (Fig. 1). The maximum diameter of the stone correlated significantly with time to stone clearance ($p = 0.031$), whereas the transverse diameter had no significant impact on the proportion of stone-free patients or time to stone clearance.

Defined were two factors having a significant impact on the time to stone clearance in an univariate analysis, namely delay in ESWL after a first colic ($p < 0.001$) and the maximum diameter of the stone ($p = 0.031$). Using a multivariate analysis revealed that the time to ESWL and the maximum diameter of the stone maintained a statistically significant impact on the time to stone clearance ($p < 0.001$; $p = 0.037$) indicating that they act independently.

A total of 95,6% of stones were radio-opaque. Stones were composed of calcium oxalate in 93.4% of cases ($n = 85$): 49.4% ($n = 42$) of these were 100% calcium oxalate monohydrate. The remainder were composed of uric acid ($n = 4$; 4.4%) and magnesium

Table 1 – Comparison of stone size and treatment results in patients treated at different time intervals after a first onset of colic pain

Time to ESWL (h)	N	Maximum stone size in mm*	No. of sessions	Subsequent time to stone clearance (days)*	No. (%) of treatment successes	No. (%) of patients with residual fragments	No. (%) of treatment failures
<24	16	7.9 ± 3.1	1.6 ± 0.8	6.4 ± 6.3	14 (87.5)	0	2 (12.5)
≥24	75	8.1 ± 2.0	1.8 ± 0,9	16.0 ± 17.8	56 (74.7)	3 (4.0)	16 (21.3)
		$p = 0.54$‡	$p = 0.5$‡	$p = 0.008$‡	$p = 0.44$†		
<48	44	8.0 ± 2.4	1.7 ± 0.9	10.7 ± 12.8	35 (79.5)	1 (2.3)	8 (18.2)
≥48	47	8.1 ± 2.1	1.9 ± 0.9	17.3 ± 19.1	35 (74.5)	2 (4.2)	10 (21.3)
		$p = 0.72$‡	$p = 0.8$‡	$p = 0.03$‡	$p = 0.85$†		
<72	59	8.1 ± 2.4	1.7 ± 0.8	10.7 ± 11.5	46 (78)	1 (1.7)	12 (20.3)
≥72	32	7.9 ± 1.9	2.1 ± 1.0	20.6 ± 22.4	24 (75.0)	2 (6.3)	6 (18.8)
		$p = 0.97$‡	$p = 0.08$‡	$p = 0.02$‡	$p = 0.5$†		
<96	65	8.0 ± 2.3	1.6 ± 0.8	10.6 ± 11,2	49 (75.4)	3 (4.6)	13 (20.0)
≥96	26	8.0 ± 2.0	2.4 ± 1.0	22.2 ± 23.5	21 (80.8)	0	5 (19.2)
		$p = 0.83$‡	$p = 0.004$‡	$p = 0.01$‡	$p = 0.54$†		

* Mean ± standard deviation.
† Pearson Test.
‡ Wilcoxon Test.

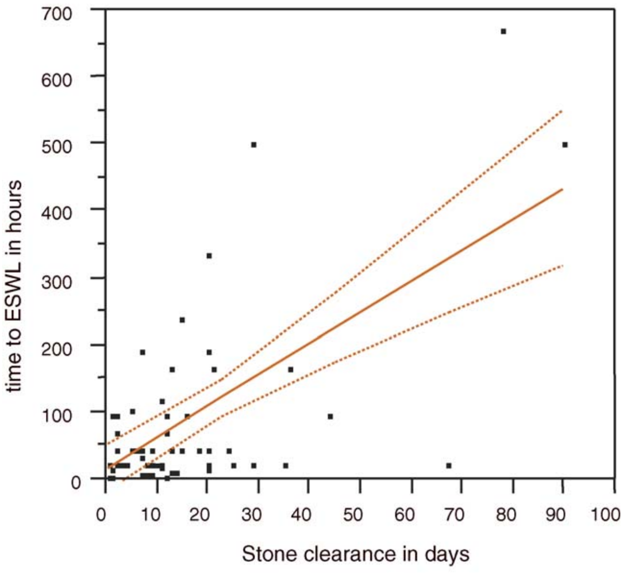

Fig. 1 – Regression analysis and curve fitting with 95% confidence curves showing the correlation between time to ESWL after a first colic episode and time to subsequent stone clearance for all stones with maximum diameters between 6.0 and 15.0 mm ($p < 0.0001$).

ammonium phosphate ($n = 2$; 2.2%). Insufficient material for analysis was collected for 3 patients. The proportion of calcium oxalate monohydrate:dihydrate did not significantly affect stone fragmentation, the number of ESWL sessions required, time to stone clearance, or the success rate. The number of stones of other composition was too small to draw further conclusions.

A percutaneous nephrostomy was inserted in 7 patients and an ureteric stent in 2 patients due to persistent colic pain or severe hydronephrosis in combination with fever and/or elevated infection parameters. Only 1 of these patients was subsequently subjected to URS and considered as treatment failure. Of the remainder, 6 became stone free and 2 harbored residual fragments ≤3 mm in diameter after 3 months. One ESWL session was interrupted after 2500 impulses due to acute septic shock. This patient also developed a significant subcapsular renal hematoma, which resolved spontaneously. A static steinstrasse occurred in 2 cases (2.2%). One passed after inserting a percutaneous nephrostomy and the other was managed by URS and extraction of the leading stone fragment. Oliguria with deterioration of renal function occurred in 1 patient (1%). This was managed by URS, basket extraction of the stone, and insertion of an ureteric JJ stent. Renal function recovered immediately thereafter.

4. Comment

The natural history of ureteral stones favors spontaneous elimination. The incidence of this depends essentially on the size of the stones and, to a lesser extent, on their location at the time of diagnosis. Spontaneous passage varies between 60% and 98% for proximal ureteric stones with diameters ≤5 mm and decreases rapidly for stones above that size, with those ≥6 mm rarely being passed [4–8]. The decision to treat small stones 3–5 mm in diameter ($n = 13$) by ESWL rather than to wait for spontaneous passage was based on the following criteria: persistent moderate or severe pain despite appropriate medical treatment, stones associated with urinary tract infection, impaired renal function, and no evidence of stone migration in combination with obstruction over a 2-week period. These criteria are generally accepted indications for intervention. The efficacy and very low morbidity associated with ESWL justify its use in the treatment of these smaller symptomatic ureteral stones, often in response to patient demand [9–12].

Ureteral edema formation over time caused by an impacted obstructive ureteral calculus theoretically impairs stone clearance after ESWL [3]. It could be assumed that the transverse diameter of a stone had a significant impact on its clearance due to impaction in the ureteral lumen. Indeed, Abdel-Khalek et al. found transverse stone diameter to be a prognostic factor for ESWL success with ureteric stones and to have a significant impact on post-ESWL stone clearance [13]. In our patients we did not observe a significant effect of transverse diameter on time to stone clearance or treatment success, but the maximum diameter of a ureteric stone significantly influenced time to stone clearance in an univariate ($p = 0.031$) and multivariate ($p = 0.037$) analysis.

The presence or degree of ultrasound detected hydronephrosis immediately before ESWL treatment did not have a significant impact on either time to stone clearance or treatment outcome. This finding has been reported in a recent study including colic and non-colic patients [14] and other studies showing no correlation of ureteral stone induced hydronephrosis with treatment success after ESWL [2,10,11,15]. Patients with complete obstructive ureteral stones, including a subgroup of 46 patients with proximal ureteral stones, have been retrospectively divided into three groups by Joshi et al. A Percutaneous nephrostomy or a double J stent were placed to relieve obstruction with subsequent ESWL treatment. The remaining patients underwent urgent ESWL within 1–14 days without prior

deobstruction. Success after urgent ESWL was greater compared to the two alternative treatment groups, although the difference was only significant towards the PCN group. Urgent ESWL appeared to be the preferred treatment option [16]. However, in cases of chronic obstruction with impaction of the calculi and fibrosis, fragmentation and expulsion of the fragments might be impeded. More than two ESWL sessions did not improve the success rate [16]. In a series of Arrabal-Martin et al. the number of shockwaves and the re-ESWL index were higher in obstructive calculi, with a higher final success rate in the group of non-obstructive calculi. Retreatment improved results from 64% to 89%, though the final success rate in calculi >1 cm, despite increasing the number of sessions, did not exceed 70% [17]. Having a comparable retreatment rate in our study, 19 (20.9%) Patients underwent a third and fourth ESWL session with a 7% improvement in success rate.

The use of various lithotripters, the heterogeneity of patients, small sample sizes, differences in follow-up, and variations in defining success rates, fragmentation and residual fragments contribute to these contradictory findings (Table 2).

This study for the first time compared prospectively the influence of a consecutive treatment delay in symptomatic patients harbouring proximal ureteral calculi. The correlation between time to ESWL after a first onset of colic pain and time to stone clearance could be explained by the development of mucosal edema within days resulting from calculi impacting the ureter [3]. Histological studies of the mucosa in the stone bed have indeed revealed a hyperplastic appearance with increased mitotic activity [18]. It is therefore beneficial for colic patients to undergo ESWL as soon as possible before such morphologic changes occur. We demonstrated a significant increase in mean time to stone clearance correlating with a consecutive delay in ESWL treatment after a first colic episode. Our finding is supported by work by Doublet et al., who reported that immediate ESWL for obstructing ureteral stones is an effective treatment giving results comparable to those of asymptomatic patients with a 1-month stone-free rate of 94% [19]. Tombal et al. recently assessed in a randomized controlled trial the efficacy of emergency ESWL (eESWL) on the short-term outcome of symptomatic ureteral stones proximal to the iliac vessels demonstrating an improved elimination [20]. Cummings et al. reported a 76% correct outcome prediction using a newly designed artificial neuronal network with 100% sensitivity [21]. The pretreatment duration of symptoms was identified as the most important factor for spontaneous stone passage.

Table 2 – ESWL treatment for symptomatic proximal ureteral stones reported in the literature

Author/Year	Lithotripter	N=†	Obstruction	Stone size (mm)	No. of sessions	Time interval between colic and 1st ESWL	No. stone free (%)	Residual fragments ≤3 mm (%)	Auxiliary procedures (%)
Tligui M. 2003	EDAP LT02: Piezoelectric	44	100%	n.a.	1.3‡	≤24 h (after admission)	36 (81.8) at 3 months	n.a.	8 (5.5)
Kravchick S. 2005	Econolith: Electrohydraulic	25	20%	7.4	n.a.	2 ± 0.7* days	18 (72) at 1 month		7 (28)
		28	24%	6.9	n.a.	26 ± 5.9* days	18 (64) at 1 month	n.a.	8 (28)
Tombal B. 2005	Siemens Lithostar: Electromagnetic	29	n.a.	6.4‡	n.a.	≤6 h (after admission)	21 (72.4) at 48 h	n.a.	6 (21)
Seitz C. 2005	Wolf Piezolith 3000: Piezoelectric	91	85.9%	7.9	1.8	93.4 ± 143.5* h	70 (76.9) at 3 months	3 (3.3)	18 (19,8)

n.a.: Not available.
* Values are means ± SD.
† No. of patients harbouring a proximal/upper third ureteral stone.
‡ For proximal and distal ureteral stones.

One prospective randomized trial comparing the outcome of emergency vs. scheduled ESWL has recently been performed [22]. Patients assigned to the study group underwent ESWL within 48 to 72 h whereas the control group was scheduled within 15 to 34 days (Table 2). The difference in overall stone free rate (72% vs. 64%) as well in stones <1 cm (94% vs. 75%) was insignificant ($p = 0.37$, $p = 0.098$). The study group had a lower number of hospitalization days and spent significantly less time for outpatient visits ($p = 0.014$, $p = 0.042$). It was therefore concluded that emergency ESWL has been a safe and effective treatment for proximal ureteral stones [22]. These findings are in accordance to our results with similar stone free rates although a stone free rate of 94% in stones <1 cm could not be achieved. Patients undergoing delayed ESWL treatment needed significantly more ESWL sessions to become stone free than patients treated with rapid ESWL ($p = 0.037$). This finding might be explained by an increase in ureteral edema formation, impactation and a subsequent lack of an expansion chamber with an increased fluid-stone interface reducing initial ESWL fragmentation rates. These results suggest that rapid ESWL in symptomatic patients is medically and economically advantageous compared to delayed treatment.

Our failure rate of 19.8% is comparable to the 18% reported by Robert et al. and by Tligui et al. both using a piezoelectric lithotripter [10,23]. Our clearance rate of 76.9% compares well with the 3-month stone-free rate (72% to 85%) of reports that establish ESWL as the treatment of choice for proximal ureteral stones without stone manipulation [22,24,25]. Proximal and mid-ureteric stones respond significantly better to shock wave application than those in the distal ureter [26]. Most guidelines still recommend ESWL as first-line treatment for proximal ureteric stones [4,26]. Whether proximal stones with diameters >10 mm should be treated with URS or ESWL remains controversial. An American Urological Association meta-analysis found the highest stone clearance rates for stones <10 mm in diameter, with 74% cleared compared with 46% of 11–20 mm stones. ESWL, URS, and percutaneous nephrolithotomy are all recommended for stones with diameters >10 mm in the proximal ureter, but URS becomes less appropriate as the stone becomes larger [4]. Other investigators favorably treat stones >10 mm in diameter primarily with URS [27,28] We found 74,2% of patients with proximal ureteric stones <10 mm in diameter to be stone free at 3 months compared with 84% of those with stones ≥10 mm ($p = 0.89$). In our view, ESWL remains the principal treatment option for most proximal ureteric stones because of its minimally invasive nature, and avoidance of general anesthesia.

5. Conclusions

Rapid ESWL after a first onset of colic pain is an effective treatment and results in accelerated stone clearance, independent of the presence or degree of hydronephrosis, without impact on the need for auxiliary procedures. When available, rapid ESWL compares favorably with delayed ESWL; therefore, treatment for symptomatic proximal ureteral stones should be initiated as soon as possible.

Acknowledgement

This study has been highlighted at the XX EAU Annual Meeting 2005, Istanbul; Turkey.

References

[1] Chaussy C, Brendel W, Schmiedt E. Extracorporeally induced destruction of kidney stones by shock waves. Lancet 1980;2:1265–8.

[2] Farsi HM, Mosli HA, Alzimaity M. In situ extracorporeal shock wave lithotripsy for primary ureteric calculi. Urology 1994;43:776–81.

[3] Mueller SC, Wilbert D, Thueroff JW. Extracorporeal shock wave lithotripsy of ureteral stones: clinical experience and experimental findings. J Urol 1986;135:831–4.

[4] Segura JW, Preminger GM, Assimos DG, Dretler SP, Kahn RI, Lingeman JE. Ureteral stones clinical guidelines panel summary report on the management of ureteral calculi. J Urol 1997;158:1915–21.

[5] Marberger M, Hofbauer J, Turk C, Hobarth K, Albrecht W. Management of ureteric stones. Eur Urol 1994;25:265–72.

[6] Miller OF, Kane CJ. Time to stone passage for observed ureteral calculi: a guide for patient education. J Urol 1999; 162:688–91.

[7] Coll DM, Varanelli MJ, Smith RC. Relationship of spontaneous passage of ureteral calculi to stone size and location as revealed by unenhanced helical CT. AJR 2002;178:101–3.

[8] Hubner WA, Irby P, Stoller ML. Natural history and current concepts for the treatment of small ureteral calculi. Eur Urol 1993;24:172–6.

[9] Assimos DG, Boyce WH, Harrisson LH, McCullough DL, Kroovand LR, Sweat KR. The role of open stone surgery since extracorporeal shock wave lithotripsy. J Urol 1989; 42:263–7.

[10] Robert M, Delbos O, Guiter J, Grasset D. In situ piezoelectric extracorporeal shock wave lithotripsy of ureteric stones. Br J Urol 1995;76:435–9.

[11] Cass AS. In situ extracorporeal shock wave lithotripsy for obstructing ureteral stones with acute renal colic. J Urol 1992;148:1786–7.

[12] Dretler SP. Ureteral stone disease. Options for management. Urol Clin North Am 1990;17:217–30.
[13] Abdel-Khalek M, Sheir K, Elsobky E, Showkey S, Kenawy M. Prognostic factors for extracorporeal shock-wave lithotripsy of ureteric stones—a multivariate analysis study. Scand J Urol Nephrol 2003;37:413–8.
[14] Seitz C, Fajkovic H, Waldert M, Tanovic E, Remzi M, Kramer G, et al. Extracorporeal shock wave lithotripsy in the treatment of proximal ureteral stones: Does the presence and degree of hydronephrosis affect success? Eur Urol 2005;49:378–83.
[15] Demirbas M, Kose AC, Samli M, Guler C, Kara T, Karalar M. Extracorporeal shock wave lithotripsy for solitary distal ureteral stones: does the degree of ureteral obstruction affect success? J Endourol 2004;18:237–40.
[16] Joshi HB, Obadeyi OO, Rao PN. A comparative analysis of nephrostomy, JJ stent and urgent in situ extracorporeal shock wave lithotripsy for obstructing ureteric stones. BJU 1999;84:264–9.
[17] Arrabal-Martin M, Pareja-Vilches M, Gutiérrez-Tejero F, Miján-Ortiz JL, Palao-Yago F, Zuluaga-Góme ZA. Therapeutic options in lithiasis of the lumbar ureter. Eur Urol 2003;43:556–63.
[18] Kim HL, Labay PC, Boyarsky S, Glenn JF. An experimental model of ureteral colic. J Urol 1970;104:390–4.
[19] Doublet JD, Tchala K, Tligui M, Ciofu C, Gattegno B, Thibault P. In situ extracorporeal shock wave lithotripsy for acute renal colic due to obstructing ureteral stones. Scand J Urol Nephrol 1997;31:137–9.
[20] Tombal B, Mawlawi H, Feyaerts A, Wese FX, Opsomer R, Van Cangh PJ. Prospective randomized evaluation of emergency extracorporeal shock wave lithotripsy (ESWL) on the short-time outcome of symptomatic ureteral stones. Eur Urol 2005;4:855–9.
[21] Cummings JM, Boullier JA, Izenberg SD, Kitchens DM, Kothandapani RV. Prediction of spontaneous ureteral calculous passage by an artificial neural network. J Urol 2000;164:326–8.
[22] Kravchick S, Bunkin I, Stepnov E, Peled R, Agulansy L, Cytron S. Emergency extracorporeal shock wave lithotripsy for acute renal colic caused by upper urinary-tract stones. J Endourol 2005;19:1–4.
[23] Tligui M, El Khadime MR, Tchala K, Haab F, Traxer O, Gattegno B, et al. Emergency extracorporeal shock wave lithotripsy (ESWL) for obstructing ureteral stones. Eur Urol 2003;43:552–5.
[24] Chang SC, Kuo HC, Hsu T. Extracorporeal shock wave lithotripsy for obstructed proximal ureteral stones. A prospective randomized study comparing in situ stent bypass and below stone catheter with irrigation strategies. Eur Urol 1993;24:177–84.
[25] Kumar A, Kumar RV, Mishra VK, Ahlawat R, Kapoor R, Bhandari M. Should upper ureteral calculi be manipulated before extracorporeal shock wave lithotripsy? A prospective controlled trial. J Urol 1994;152:320–3.
[26] Tiselius HG, Ackermann D, Alken P, Buck C, Conort P, Gallucci M. Guidelines on urolithiasis. Eur Urol 2001; 40:362–71.
[27] Anagostou T, Tolley D. Management of ureteric stones. Eur Urol 2004;45:714–21.
[28] Pearle MS, Nadler R, Bercowsky E, Chen C, Dunn M, Figenshau RS. Prospective randomized trial comparing shock wave lithotripsy and ureteroscopy for management of distal ureteral calculi. J Urol 2001;166:1255–60.

Editorial Comment

Riccardo Autorino, *Naples, Italy*
ricautor@tin.it

When an active ureteral stone treatment is warranted, the best procedure to choose is dependent on several factors, besides stone size and location, including operators experience, patients preference, available equipment and related costs.

Two decades after its introduction in clinical practice, extracorporeal shockwave lithotripsy (SWL) is nowadays used for the treatment of most upper urinary stones because of its minimally invasive nature, lack of serious complications and avoidance of general anaesthesia. Nevertheless, its role as a first line therapeutic option, applied rapidly after the onset of renal colic, has deserved very limited attention. Few reports on this subject can be found in the literature and, so far, only two randomized trials have been reported [1,2], even both with encouraging results of ‘‘emergency’’ SWL, that is an attractive proposition as it can result in both stone disintegration and relief from acute obstruction.

The authors of the present report are to be congratulated for conducting a prospective study investigating the relationship between rapid SWL (with a third generation lithotripter) after a first colic episode and subsequent time to complete stone clearance in a selected study population. This study, even if non-randomized, correlates for the first time the influence of a consecutive treatment delay in patients harbouring proximal ureteral stones. The analysis of factors having an impact on the time to stone clearance showed that only two of them, namely delay in SWL treatment after a first colic and maximum stone diameter, resulted independently significant.

The promising results obtained after rapid SWL are in agreement with others from previously reported trials. The improved success rates in those undergoing emergency SWL can be explained by the fact that ureteral edema and fibrosis are less extensive in the early periods of stone impaction [3].

Even if patients undergoing delayed SWL treatment needed significantly more sessions to become stone free, the findings from the present report cannot allow answering on the cost-effectiveness of rapid SWL. Thus, besides its recognized medical advantages, the supposed economic advantages of emergency SWL remains to be demonstrated and trials specifically addressing this issue are needed. In this respect, even if the economics of urolithiasis are of great importance due to the burden of the disease, it should be recognized that there is a wide disparity in the cost of stone treatment between health care systems from different countries and this fact still represents an unsolved problem [4].

Historically, ureteroscopy has remained less widely accepted and more controversial than SWL for proximal ureteral stones. In the recent years, the miniaturization of ureteroscopes together with the introduction of the holmium laser has improved stone free rates and decreased complication rates, making ureteroscopy more than an attractive alternative and widening its indications [5]. Notwithstanding this phenomenon, significant data on the ureteroscopic management of ureteral stone in an emergency setting are still lacking. Moreover, despite these technological advances, ureteroscopy remains much more challenging than SWL for proximal ureteral stones.

On the basis of the findings from this and other previous reports, I believe emergency SWL for obstructive proximal ureteral stones could be widespread. Of course, it requires appropriate lithotripter facilities for emergency use and this could still represent a major limitation. In this setting, further randomized trials are warranted in order to corroborate the positive data from the current literature.

References

[1] Tombal B, Mawlawi H, Feyaerts A, Wese F, Opsomer R, Van Cangh PJ. Prospective randomized evaluation of emergency extracorporeal shock wave lithotripsy (ESWL) on the short-time outcome of symptomatic ureteral stones. Eur Urol 2005;47:855–9.

[2] Kravchick S, Bunkin I, Stepnov E, Peled R, Agulansky L, Cytron S. Emergency extracorporeal shockwave lithotripsy for acute renal colic caused by upper urinary tract stones. J Endourol 2005;19:1–4.

[3] Joshi HB, Obadeyi OO, Rao PN. A comparative analysis of nephrostomy, JJ stent and urgent in situ extracorporeal shockwave lithotripsy for obstructing ureteric stones. BJU International 1999;84:264–9.

[4] Lotan Y, Pearle M. Economics of stone management. EAU Update series 2005;3:51–60.

[5] Gettman MT, Segura JW. Management of ureteric stones: issues and controversies. BJU Int 2005;95(suppl 2):85–93.
