# Supplementary material for: Efficacy of emergency extracorporeal shock wave lithotripsy in the treatment of ureteral stones: a meta-analysis
Source: BMC Urol. 2023 Apr 4;23:56. doi: 10.1186/s12894-023-01226-5 (PMC10074806; doi:10.1186/s12894-023-01226-5)
Supplement: Supplementary file 8 — Additional File 8: Tombal 2005 [file 12894_2023_1226_MOESM8_ESM.pdf]

European Urology

European Urology 47 (2005) 855–859

Prospective Randomized Evaluation of Emergency Extracorporeal Shock Wave Lithotripsy (ESWL) on the Short-Time Outcome of Symptomatic Ureteral Stones

Bertrand Tombal*, Hadi Mawlawi, Axel Feyaerts, Francois X. Wese, Reinier Opsomer, Paul J. Van Cangh

Service d'Urologie, UCL Saint Luc, Avenue Hippocrate, 10, B-1200 Brussels, Belgium

Accepted 3 March 2005

Available online 17 March 2005

Abstract

Objective: Here, we report the results of a randomized controlled trial (RCT) assessing the efficacy of emergency ESWL (eESWL) on the short-term outcome of symptomatic ureteral stones.

Material: The trial enrolled 100 patients admitted in emergency room for renal colic caused by a ureteral radiolucent stone. Patients were randomized to medical therapy alone or combined with eESWL. eESWL was performed within 6 hours of the onset of renal colic without specific analgesia on a Lithostar lithotripter (Siemens Medical, Munich, Germany). The primary endpoints were the proportion of patients stone free rate after 48 hours (SF-48) and the cumulative proportion of patients discharged from the hospital after 48 and 72 hours.

Results: Ureteral stone's location was proximal and distal in respectively 46% and 54% of the patients; stone's mean size was 5.5 mm (range 2–10 mm). Median hospital stay was 3 days, ranging from 1 to 14 days. SF-48 in the control group varied from 76% for distal stones <5 mm to 28.6% for proximal stones >5 mm, averaging at 61%. On average, eESWL increased SF-48 by 13% (p: 0.126), the gain strictly depending on stone size and location. SF-48 increase ranged from 40% for proximal stones >5 mm to 1.8% for distal stone <5 mm. On average, eESWL increased the median duration of hospital stay by one day. This mean negative impact results from ESWL increasing significantly the duration of hospital stay in case of distal stone, while slightly shortened it for stones located proximally.

Conclusion: This study demonstrated for the first time that rapidly performed ESWL is a valuable therapeutic option to improve elimination of ureteral stones and shorten duration of hospital stay, proven that the stone is located proximally to the iliac vessels.

© 2005 Elsevier B.V. All rights reserved.

Keywords: ESWL; Ureteral stones; Emergency

1. Introduction

Ureteral stone disease is one of most prevalent of urological disorders, and of the most painful [1]. It is estimated that as much as 5% of the population will be affected by urinary stones during their lifetime. Since most stones pass out of the urinary tract with no or little discomfort, most patients are treated in outpatient's facilities [1].

Patient are hospitalized when active relieve of urinary obstacle is emergently required, e.g. in case of high fever, urinary tract infection, septic shock, anuria, or impaired renal function [2,3]. In most cases, however, hospitalization is simply motivated by the need of having patient under intravenous infusion because the pain was resistant to first-line oral or intra-rectal

Abbreviations: ESWL, Extracorporeal shock wave lithotripsy; CRP, C-reactive protein; ER, Emergency Room; RCT, randomized controlled trial; eESWL, emergency extracorporeal shockwave lithotripsy.

* Corresponding author. Tel. +32 2 7645540; Fax: +32 2 7645580.
E-mail address: bertrand.tombal@fymu.ucl.ac.be (B. Tombal).

0302-2838/$ – see front matter © 2005 Elsevier B.V. All rights reserved.
doi:10.1016/j.eururo.2005.03.006

ELSEVIER

treatment. In these patients, the treatment focus on securing fast pain relief, achieving quick elimination of the stone and shortening the duration of hospital stay, with minimal morbidity and maximal optimal cost-effectiveness.

Extracorporeal shockwave lithotripsy (ESWL) is the treatment of choice for moderately sized, uncomplicated ureteral stones [3,4]. ESWL is a simple, robust and safe procedure and is usually recommended for stones resistant to medical treatment in absence of absolute indication of ureteral drainage [5]. Interestingly, the role of ESWL as a first line therapy, applied rapidly after the onset of renal colic, has deserved very limited attention. So far only three non-randomized studies have suggested that emergency ESWL (eESWL) is an appealing treatment strategy for symptomatic ureteral stones [6–8].

Here, we have prospectively investigated whether rapidly scheduled ESWL, presently referred as emergency ESWL (eESWL), improved stone's elimination and shortened hospital stay. To test this hypothesis we have conducted a prospective randomized controlled trial to compare medical therapy alone or combined with eESWL.

2. Material and methods

The present RCT enrolled after oral consent 100 patients admitted in emergency room (ER) between 01/01/2001 and 01/02/2003 for the treatment of a symptomatic ureteral stone.

Admission work-up included: monitoring of vital parameters; rectal temperature; physical examination; blood test for leucocytes, CRP, urea, creatinine; urine analysis and culture. Primary imaging of the patient was performed by helical unenhanced computed tomography (HUCT) of the abdomen, according to modern recommendations [9–12]. Plain abdominal X-ray was added to confirm radiolucency of the stone. Initial characterization of the stone was based on the HUCT imaging and included stone size (largest transversal diameter measured by CT) and stone location (proximal or distal ureter when located respectively above or below the crossing with the iliac artery).

2.1. Inclusion criteria were

- Patient with acute flank pain caused by ureteral stone.
- Hospitalisation motivated by the patient requiring intravenous administration (IV) of drug and fluid either because of the onset of nausea and/or vomiting hampering administration of oral drug or pain persisting despite correct use of oral drug.
- Radiolucent stone clearly identify on play X-ray allowing ESWL

2.2. Exclusion criteria

- Stone >10 mm in largest diameter
- Dilatation of the renal pelvis >30 mm or presence of a perirenal urinoma.
- Rectal temperature > 38 °C
- Blood leukocytes >20,000/dl, serum creatinine >1.8 mg/dl, urine leukocytes >25/field
- Stone located in the renal pelvis or the pyelo-ureteral junction.
- Solitary kidney, prior history of ureteral stricture or tumour
- Contraindication to ESWL

2.3. Treatment randomization and schedule

Patients were randomized at admission in ER (Day 0) for medical treatment or medical treatment plus eESWL. No stratification parameters were used. All patients received intravenous perfusion of glucose 5% in saline. Baseline medical treatment started at admission in ER and included IV administration of antispasmodic drug, butylhyoscine 20 mg, and NSAID, ketorolac 30 mg. Thereafter, ketorolac 30 mg was administered IV systematically every 12 h. Butylhyoscine 20 mg was used as first-line on-demand additional analgesia, with a minimal interval of 4 h. Morphine analogue, piritramide 10 mg, was administrated intramuscularly on demand only in case of persisting pain despite baseline treatment.

eESWL procedures were performed on a Siemens Lithostar Multiline within 6 h of admission. No additional analgesia was used for ESWL procedure. An average of 3500 hits were administered while incrementing progressively the power up to the maximal tolerable dose. Control plain X-ray of the abdomen was performed on day 1 and day 2. Interventional procedures (JJ stent ± uretero-ureteroscopy) were performed within 72 hours only in case of worsening of the symptom and impossibility to manage them medically, apparition of fever or modification of laboratory findings.

2.4. Study endpoints

2.4.1. Primary endpoint

Proportion of patients stone free at 48H00 post-admission (SF-48 h). Patients were stone free only after complete elimination of ureteral stone on plain X-rays.

2.4.2. Secondary endpoint

Cumulative percentage of patients released after 48 and 72 h. To secure the endpoint and minimize patient- or physician-driven discharge's bias, patients were strictly released of the hospital once they did not required on-demand additional drugs (butylhyoscine or piritramide) for 12 consecutive hours. Patients were released only based on this drug requirement, independently of the stone elimination.

2.4.3. Statistics

The sample size was calculated using the following estimations: α value: 0.005; power: 0.80; expected SF-48 h: 50%; expected improvement by ESWL: 15%. Continuous variable are described by the mean value and the 95% lower and upper confidence interval (95%CI). Comparison between variables was performed using the independent sample *t*-test procedure. Comparison between distributions is performed using odds ratio and χ^2 procedures. All analyses were performed by SPSS Statistical Software.

3. Results

Patient's characteristics at inclusions are reported in Table 1. Male/female ratio was 8/2. Average age is 43 years old. Age, sex ratio, and side were equally distributed between cohorts. Size and stone localization

Table 1
Patients characteristics

n	Total 100	Medical therapy 50	ESWL 50	p^{*}
Sex ratio (M/F)	83/17	43/7	40/10	
Side (L/R)	51/49	27/23	24/26	
Age (years)				
mean	43	42	44	
95% CI lower-upper	(40–45)	(39–46)	(40–47)	
Stone location				
Proximal ureter	46	17	29	0.13*
Distal ureter	54	33	21	
Stone size (mm)				
Mean	5.6	4.8	6.38	0.01**
95% CI lower-upper	5.1– 6.02	4.23–5.37	5.7–6.9	
Median	5	4	6	
Range	2–10	2–10	2–10	
<5 mm	52	14	34	
>5 mm	48	36	16	
Duration of hospital stay (days)				
Mean	4.08	3.92	4.24	
Median	3	3	4	

* *p* value is measured by the Pearson Chi-Square procedure.
** *p* value is measured by the Independent-Samples *t*-test procedure.

Table 3
Cumulative percentage of patient released from hospitalization at day 1, 2 and 3

	Cumulative percentage of patient discharged from hospitalisation after			
	48 hours		72 hours	
%	Medical therapy	ESWL	Medical therapy	ESWL
Overall series	36	24	56	46
Stone size				
≤5 mm	44	40	66	53
>5 mm	14	17	28	42
Stone location				
Distal	45	28	63	52
Proximal	17	20	41	41
Stone size and location				
Distal ≤5 mm	50	44	69	55
Distal >5 mm	28	16	42	50
Proximal ≤5 mm	30	33	60	50
Proximal >5 mm	0	18	14	39

were not balanced between medical treatment and eESWL cohorts reflecting the absence of stratification. There were more small distal stones in the surveillance group. eESWL was administered in 50 patients. The procedure could be completed successfully in 47 patients and aborted in 3 patients for pain. Eight patients mentioned macroscopic hematuria afterwards, none requiring specific treatment

3.1. Stone free rate at 48 hours (Table 2)

On average, 61% of the stones passed with medical treatment by 48 h. eESWL improved SF-48 by 13%, thus less than the 15% expected when designing the trials. In intent-to-treat analysis, the results did not reach the level of statistical significance ($p = 0.126$). Group analysis were performed by combining stone location (distal vs. proximal) and size (largest diameter <5 or >5 mm). In the medical treatment group, SF-48 decreased with size and location from 76% for distal stones <5 mm to 28.6% for proximal stones >5 mm. eESWL improved SF-48 for any stone size and location. The amplitude of the benefit, however, was more stringent for stone located proximally and with a size >5 mm.

3.2. Cumulative proportion of patient relieve from hospitalization (Table 3)

Median and average hospital stay were 3 and 3.1 days (95% lower and upper confidence interval: 2.6–3.6 days). eESWL increased median hospital stay by 1 day. eESWL decreased the proportion of patient released from the hospital by 12% at day 2, and by 10% at day 3. This effect largely depended of the size and location of the stone. In patients with stones <5 mm located distally, eESWL decreased the proportion of patients released from the hospital at day 2 and day 3 by 20%, while, in contrast, eESWL increased it by 20% in patients with proximal stones >5 mm.

3.3. Need for additional procedures

Retrograde ureteroscopy with fragmentation of the stone and/or insertion of a JJ stent was performed in 19 patients, 8 from the medical treatment cohort and 11 from the ESWL cohort (*p* for χ^2 test: 0.306). Detailed distribution is reported in Table 4. Indication was

Table 2
Proportion of patients (in percents) stone free at 48 hours (%)

	Medical therapy		ESWL		O.R.*	p^{**}
	n		*n*			
Total	50	61.2	50	74.0	1.80	*0.126*
Distal	33	71.9	21	76.2	1.25	*0.490*
Proximal	17	41.2	29	72.4	3.75	**0.038**
≤5 mm	36	68.6	16	81.3	1.98	*0.278*
>5 mm	14	42.9	34	70.6	3.2	*0.071*
Distal ≤5 mm	26	76.0	9	77.8	1.10	*0.649*
Distal >5 mm	7	57.1	12	75.0	2.25	*0.905*
Proximal ≤5 mm	10	50.0	7	85.7	6.00	*0.160*
Proximal >5 mm	7	28.6	22	68.2	5.35	*0.080*

* Odds Ratio (O.R.) of being stone free at 48 hours when comparing surveillance to ESWL.
** *p* calculated by the χ^2 procedure.

Table 4
Ureteroscopy

N (*N* successful)	Medical therapy	ESWL
Overall	8	11
Proximal	7 (6)	6 (3)
Distal	1 (1)	5 (3)
≤5 mm	3 (3)	5 (2)
>5 mm	5 (4)	7 (4)

persistent pain despite optimal analgesic use in 17 patients, and increase of serum creatinine in 2 patients. The procedure achieved complete fragmentation and removal of the stone in 13 patients, including 6/8 from the medical treatment cohort and 7/11 from the ESWL cohort.

4. Discussion

Urinary stone disease is one of the most frequent pathology in modern urology. Most urinary stones pass uneventfully through the collecting system [13]. Stone obstruction and renal colic occur although often and account for most of emergency admission in Urology. According to the U.S. National Institutes of Health, 1 person in 10 develops kidney stones during their lifetime and renal stone disease accounts for 7–10 of every 1000 hospital admissions. Hopefully, most renal colic can be treated in outpatient facilities, pain relief being commonly achieved by NSAID or antispasmodic drugs administered orally or intra-rectally (1,4,14). The spontaneous rate of elimination of the stones depends on the stone size and position in the ureter [13]. In a recent prospective study using unenhanced helical CT, Coll et al. have demonstrated that the spontaneous passage rate for stones 1 mm in diameter was 87%; for stones 2–4 mm, 76%; for stones 5–7 mm, 60%; for stones 7–9 mm, 48%; and for stones larger than 9 mm, 25% [15]. In the same series, spontaneous passage rate was also dependent on stone location (48% for stones in the proximal ureter, 60% for mid ureteral stones, 75% for distal stones, and 79% for ureterovesical junction stones). In addition to size and location, there are also other interfering factors such as obesity, level of renal obstruction and type of medical therapy [2,16,17].

In the last 20 years, the development and constant improvement of minimally invasive techniques such as ureteroscopy with in situ lithotripsy or laser fragmentation and ESWL has prompted urologists toward more aggressive attitude. Although observation is still recommended for stones measuring less than 4 mm in diameter, most international guidelines recommend today active removal of all stone exceeding 5–7 mm, proven that they have resisted to medical therapy. Active removal is also strongly indicated in patient with persistent pain despite adequate medical treatment, acute obstruction with impaired renal function or solitary functional kidney, urinary tract infection, risk or suspicion of urosepsis [13,14,18]. In case removal of ureteral stone is warranted, the main debate centres nowadays around the choice of extracorporeal shock wave lithotripsy or endoscopic management combined with laser or mechanic fragmentation [4,19–21]. This issue of optimal management of ureteral stones has been reviewed in detail by Anagnostou and Tolley [14].

More commonly, hospitalization is also required to manage intractable pain resistant to oral or intra-rectal therapy. While the main goal of therapy should then still be oriented toward fast pain relief and safe stone removal, it is also critical to achieve rapid discharge from the hospital. So far this issue has been mainly tackled by adapting medical therapy, e.g. by adding calcium-channel blockers, corticoids or even alpha-blockers to standard treatments [16,17,22,23]. Overall although, there is still considerable scope for improving the process of delivery of emergency interventional care and reducing inpatient stay.

In institution equipped with ESWL then comes the question whether applying ESWL shortly after the onset of renal colic could help resolving this issue. Interestingly enough, although ESWL is widely considered as one of the treatment of choice of ureteral stones, its use as an immediate therapeutic tool in an ER setting has not deserved that much attention yet. To our knowledge, only reports by Gonzalez Enguita et al. [8], Doublet et al. [6], and Tligui et al. [7] addressed its potential interest. Tligui et al reported in 2003 their experience of 200 patients suffering from acute renal colic and treated with emergency ESWL within 24 h. Based on this observation, they advocated a more widespread used of the technique based on a high stone free rates after three months and a low morbidity. The study however was not randomized.

Here, we report the results of the first randomized trial addressing the role of eESWL in 100 patients requiring hospitalization for the management of renal colic. We have prospectively compared standard medical treatment with NSAID and antispasmodic to medical treatment plus eESWL, performed without analgesia on a Lithostar lithotripter within 6 h following admission in the ER. For this exploratory trial, randomization procedure did not include stratifying patient. This resulted in a slight imbalance between cohorts in stone size and location. On the average, this study showed that eESWL increased the proportion of patient stone-free at 48 h by 13% while increased the median duration of hospitali-

zation by one day. Noteworthy, the effect of eESWL on both endpoints strictly relies on the size and location of the stone. eESWL increased both SF-48 h and proportion of patients discharged from the hospital at 72 hours by respectively 40% and 25% when the stone was located proximally and >5 mm, and should be strongly recommended in these cases. In contrast, when the stone is located distally from the crossing of the iliac artery, eESWL only slightly increased stone free rate by 5% while decreasing the proportion of patients released from hospitalization at 48 h and 72 h. In addition, it seemed from a limited number of patients requiring endoscopic procedure that ESWL hampered access to the stone since ureteroscopy failed to achieve complete evacuation of the stones in 3/5 distal stones treated with ESWL.

Theoretically, the addition of EWSL in patients under medical treatment would be expected to increase the overall management cost. However, calculation of cost-effectiveness implies much more than the added cost of ESWL, i.e. length and cost of hospitalization stay, recovery time and need for additional therapy. Therefore, this study does not allow answering on the cost-effectiveness of emergency ESWL.

5. Conclusions

This is the first RCT designed to assess the value of emergency ESWL on symptomatic ureteral stones. Its conclusions are double. Firstly, it confirms that medical therapy is a valuable options for small stones located in the distal ureter since three third of stone will pass within 48 h. In contrast, spontaneous passage is observed in 50% or less of the patients when the stone is located in the proximal ureter. Secondly, the study suggests eESWL is a valuable option since it increases the proportion of patient stone free after 48 h. This effect is modest in patient with distal stones but spectacular in patients with proximal stone, improving by more than 35% the success rate. Further evaluations are on their ways to assess cost-effectiveness.

References

[1] Teichman JM. Clinical practice Acute renal colic from ureteral calculus. N Engl J Med 2004;350:684–93.

[2] Deliveliotis C, Chrisofos M, Albanis S, Serafetinides E, Varkarakis J, Protogerou V. Management and follow-up of impacted ureteral stones. Urol Int 2003;70:269–72.

[3] Tiselius HG, Ackermann D, Alken P, Buck C, Conort P, Gallucci M. Guidelines on urolithiasis. European Urology 2001;40:362–71.

[4] Arrabal-Martin M, Pareja-Vilches M, Gutierrez-Tejero F, Mijan-Ortiz JL, Palao-Yago F, Zuluaga-Gomez A. Therapeutic options in lithiasis of the lumbar ureter. Eur Urol 2003;43:556–63.

[5] Tombolini P, Ruoppolo M, Bellorofonte C, Zaatar C, Follini M. Lithotripsy in the treatment of urinary lithiasis. J Nephrol 2000;13(Suppl 3):S71–82.

[6] Doublet JD, Tchala K, Tligui M, Ciofu C, Gattegno B, Thibault P. In situ extracorporeal shock wave lithotripsy for acute renal colic due to obstructing ureteral stones. Scand J Urol Nephrol 1997;31:137–9.

[7] Tligui M, El Khadime MR, Tchala K, Haab F, Traxer O, Gattegno B, et al. Emergency extracorporeal shock wave lithotripsy (ESWL) for obstructing ureteral stones. Eur Urol 2003;43:552–5.

[8] Gonzalez Enguita C, Cabrera Perez J, Calahorra Fernandez FJ, Garcia Cardoso J, Vela Navarrete R. [Efficient, immediate or emergency ESWL: an attractive strategic alternative to be considered in the treatment of renal colic!]. Actas Urol Esp 2000;24:721–7.

[9] Dalla Palma L, Pozzi-Mucelli R, Stacul F. Present-day imaging of patients with renal colic. Eur Radiol 2001;11:4–17.

[10] Sourtzis S, Thibeau JF, Damry N, Raslan A, Vandendris M, Bellemans M. Radiologic investigation of renal colic: unenhanced helical CT compared with excretory urography. AJR Am J Roentgenol 1999;172:1491–4.

[11] Wang LJ, Ng CJ, Chen JC, Chiu TF, Wong YC. Diagnosis of acute flank pain caused by ureteral stones: value of combined direct and indirect signs on IVU and unenhanced helical CT. Eur Radiol 2004;14:1634–40.

[12] Yilmaz S, Sindel T, Arslan G, Ozkaynak C, Karaali K, KabaalioŸlu A, et al. Renal colic: comparison of spiral CT, US and IVU in the detection of ureteral calculi. European Radiology 1998;8:212–7.

[13] Segura JW, Preminger GM, Assimos DG, Dretler SP, Kahn RI, Lingeman JE, et al. Ureteral Stones Clinical Guidelines Panel summary report on the management of ureteral calculi. The American Urological Association. J Urol 1997;158:1915–21.

[14] Anagnostou T, Tolley D. Management of ureteric stones. Eur Urol 2004;45:714–21.

[15] Coll DM, Varanelli MJ, Smith RC. Relationship of spontaneous passage of ureteral calculi to stone size and location as revealed by unenhanced helical CT. AJR Am J Roentgenol 2002;178:101–3.

[16] Dellabella M, Milanese G, Muzzonigro G. Efficacy of tamsulosin in the medical management of juxtavesical ureteral stones. J Urol 2003;170(6 Pt 1):2202–5.

[17] Porpiglia F, Ghignone G, Fiori C, Fontana D, Scarpa RM. Nifedipine versus tamsulosin for the management of lower ureteral stones. J Urol 2004;172:568–71.

[18] Tiselius HG, Ackermann D, Alken P, Buck C, Conort P, Gallucci M. Guidelines on urolithiasis. Eur Urol 2001;40:362–71.

[19] Hosking DH, Smith WE, McColm SE. A comparison of extracorporeal shock wave lithotripsy and ureteroscopy under intravenous sedation for the management of distal ureteric calculi. Can J Urol 2003;10:1780–4.

[20] Hochreiter WW, Danuser H, Perrig M, Studer UE. Extracorporeal shock wave lithotripsy for distal ureteral calculi: what a powerful machine can achieve. J Urol 2003;169:878–80.

[21] Wu CF, Shee JJ, Lin WY, Lin CL, Chen CS. Comparison between extracorporeal shock wave lithotripsy and semirigid ureterorenoscope with holmium:YAG laser lithotripsy for treating large proximal ureteral stones. J Urol 2004;172(5 Pt 1):1899–902.

[22] Cooper JT, Stack GM, Cooper TP. Intensive medical management of ureteral calculi. Urology 2000;56:575–8.

[23] Porpiglia F, Destefanis P, Fiori C, Scarpa RM, Fontana D. Role of adjunctive medical therapy with nifedipine and deflazacort after extracorporeal shock wave lithotripsy of ureteral stones. Urology 2002;59:835–8.
