# Supplementary material for: Efficacy of emergency extracorporeal shock wave lithotripsy in the treatment of ureteral stones: a meta-analysis
Source: BMC Urol. 2023 Apr 4;23:56. doi: 10.1186/s12894-023-01226-5 (PMC10074806; doi:10.1186/s12894-023-01226-5)
Supplement: Supplementary file 9 — Additional File 9: Uguz 2012 [file 12894_2023_1226_MOESM9_ESM.pdf]

Urol Res (2012) 40:739–744
DOI 10.1007/s00240-012-0490-2

ORIGINAL PAPER

Immediate or delayed SWL in ureteric stones: a prospective and randomized study

Sami Uguz · Temucin Senkul · Hasan Soydan · Ferhat Ates · Ilker Akyol · Ercan Malkoc · Kadir Vehbi Baykal

Received: 29 February 2012 / Accepted: 22 June 2012 / Published online: 5 July 2012
© Springer-Verlag 2012

Abstract The objective is to compare immediate and delayed SWL as a treatment for ureter stones between 5 and 20 mm. Patients who applied to the emergency department with single, radio opaque ureteric stone of 5–20 mm size were included in the study. Patients were randomized into immediate and delayed SWL groups. SWL were carried out after pain relief in the delayed group. Maximum of three SWL sessions were applied to the patients with stones of 5 mm or bigger in diameter, leaving at least 3 days interval in-between. Stone-free rate after first session was similar in two groups. When CIRF was also considered as success, the success rate of SWL in the immediate SWL group was higher after first session ($p = 0.02$). Immediate SWL had a greater success rate in upper ureteric stones ($p = 0.019$). Overall average time required for elimination of stones was shorter in immediate SWL group than delayed SWL group ($p = 0.033$). The success rate after first SWL session (including CIRF) was 59 % for patients with chronic pain in the delayed group and 100 % for patients with acute pain in the immediate group. According to the hydronephrosis grade, success rates were 71 and 44.4 % for patients with grade 1 or no hydronephrosis in the immediate SWL group, and for patients with grade 2 or larger hydronephrosis in the delayed SWL group, respectively, after first SWL session. Immediate SWL insures stone-free status in a shorter time in cases with renal colicky pain and ureteral stones, particularly upper ureteral stones.

Keywords Ureter · Stone · Treatment · SWL

Abbreviations

SWL	Extracorporeal shock wave lithotripsy
EAU	European Association of Urology
AUA	American Urological Association
PAR	Plain abdominal radiographs
URS	Ureteroscopic lithotripsy
PNL	Percutaneous nephrolithotomy
USG	Ultrasonography
NCCT	Noncontrast computed tomography
ER	Emergency department
VPS	Visual pain scale
CIRF	Clinically insignificant residual fragment

Introduction

Ureteric stones may cause significant obstruction and may require interventional treatment, depending on the size and location of the stone [1]. A conservative approach may lead to complications such as flank pain and need for emergency referrals, loss of labor, urinary tract obstruction, infection, and loss of renal function [2]. Upto 98 % of the newly diagnosed cases, proximal or distal single ureteric stones smaller than 0.5 cm can pass spontaneously, while bigger ones can pass at a reduced rate with an increased complication rate.

Extracorporeal shock wave lithotripsy (SWL) has been employed frequently in the treatment of urinary stone disease, being a simple, effective and reliable procedure

S. Uguz · T. Senkul · H. Soydan (✉) · F. Ates · I. Akyol · K. V. Baykal
Urology Department, GATA Haydarpasa Teaching Hospital, Uskudar, Istanbul 34668, Turkey
e-mail: hasan_soydan@yahoo.com

E. Malkoc
Corlu Military Hospital, Tekirdag, Turkey

that can be done without anesthesia [3]. SWL has been the treatment of choice in most of the ureteric stones, as recommended in the Ureteral Calculi Guidelines of both European Association of Urology (EAU) and American Urological Association (AUA) [4]. Success rate of SWL in the treatment of ureteric stones is approximately 80 % [2]. Various factors determine the success rate such as location, size and type of stone, as well as impaction of the stone and renal function [5].

If stone remains stationary in the ureter, mucosal edema develops at the stone site within days [6], and the success rate of SWL decreases. This is because there is no room for the stone to expand and there is no sufficient liquid interface around the stone [7]. Therefore, an early treatment is preferable. The aim of the immediate SWL is to increase treatment success by performing SWL before edema forms within the ureter. Despite the few number of studies showing that immediate SWL is more successful in the treatment of ureteric calculi, variations in terms of the study parameters make it difficult to reach a concrete conclusion regarding the issue [1, 8–14]. Our study aims to compare immediate SWL to delayed SWL using standardized parameters of diagnosis, treatment and outcome analysis.

Patients and methods

Our study started upon Institutional Review Board approval. Patients who applied to the emergency department (ER) with renal colic and who were diagnosed with a radioopaque ureteric stone of 5–20 mm size according to the plain abdominal radiographs (PAR) were included in the study. Informed consent from each patient was obtained. Our exclusion criteria were (suspicion of) pregnancy, history of ureteral stenosis or ureteral tumor, open/endoscopic operations [ureteroscopic lithotripsy (URS), percutaneous nephrolithotomy (PNL), etc.], bleeding diathesis, severe cardiovascular and pulmonary disease, solitary kidneys, acute kidney failure (serum creatinine level greater than 1.5 mg/dl), severe urinary tract infection or urosepsis, anticholinergic and alpha blocking agent use, age under 15 or above 80 years. Patients were evaluated with urine analysis, hemogram, serum urea and creatinine levels, activated partial thromboplastin time (aPTT), and prothrombin time/international normalized ratio (PTT/INR) tests. Body mass index (BMI) was calculated.

Whenever PAR revealed an opacity suspicious of a ureteric stone, a urinary ultrasonography (USG) was also performed. When dilatation of the collecting system was detected, hydronephrosis was graded as follows: mild (grade 1), if only renal pelvis was dilated; moderate (grade 2), if renal pelvis and calices were dilated; severe, if renal pelvis and calices were dilated and renal parenchyma has become thin (grade 3) [15]. Later, all cases were evaluated with abdominopelvic noncontrast computed tomography (NCCT) (64 slice multidetector computed tomography Brilliance scanner Philips Healthcare, Best, The Netherland) to detect the location and the size of the stones. Stones were classified as upper part (from ureteropelvic junction to the upper edge of sacrum), middle part (in-between the upper and lower borders of sacrum) and lower part (in-between lower border of sacrum and bladder) stones based on the images on PARs [16].

Patients were randomized into two groups by means of a computer software, such that, immediate and delayed SWL treatments were scheduled within 24 h and within 3–7 days from referral, respectively. Patients in the delayed SWL group received a non-steroidal anti-inflammatory drug (diclofenac sodium), and they were recommended an oral hydration so as to urinate 2 liters per day. They were cautioned that they should come back to the ER in the event of fever, severe nausea and vomiting, or any pain that does not respond to analgesic medication. SWL treatments were carried out after pain relief in this group. No patients received alpha blockers during SWL treatment, and no ureteric stents were inserted in any patient prior to the SWL until the end of third session. SWL procedure was performed by the same urologist in all cases using a lithotripter with an electromagnetic generator (Siemens Lithoskop TM, Germany) with the patients in supine position at a shock wave rate 90 per minute. Maximum pulses per session were 3,000 for lower ureteric stones and 3,500 for upper and middle part stones. However, treatment was stopped before completion of the scheduled pulses if the stone image disappeared or if the stone was disintegrated.

SWL outcome was assessed with NCCT on the third day after first session. Stone-free status was accepted as success. Stones with 4 mm or less in diameter were considered as clinically insignificant residual fragments (CIRF), while bigger stones were accepted as failure. A maximum of three SWL sessions were applied to the patients with stones of 5 mm or bigger, leaving at least 3 days interval in-between. Other treatment modalities were performed if SWL failed after three sessions. Number of sessions per patient, number of shocks per session, and complications of the treatment were recorded. Statistical evaluation was performed using a computerised software (SPSS® 16.0, SPSS Inc. Chicago, IL, USA). Data were analyzed for compliance with normal distribution. Data that do and do not comply with normal distribution were analyzed with *t* test as independent samples, and with the Mann–Whitney *U* and the Fisher's exact test, respectively. Categorical data were compared with the Chi-square test. A p value of 0.05 was considered as the threshold for statistical significance.

Results

A total of 63 patients, 32 (50.8 %) in the immediate SWL and 31 (49.2 %) in the delayed SWL group, were included in the study between December 2010 and April 2011. There were 25 (78.1 %) males and 7 (21 %) females in the immediate SWL group and 25 (80.6 %) males and 6 (19.4 %) females in the delayed SWL group. Mean stone sizes of immediate and delayed groups were 8.12 ± 3.16 and 8.8 ± 2.87 mm, respectively, which are statistically similar. Average age was 37.19 (18–79) years and average BMI was 26.3 kg/m^2. Body weights, BMIs and stone sizes were similar between the groups ($p > 0.05$) (Table 1).

There was no difference between the two groups in terms of laterality and location of the stones ($p = 0.171$). Delayed SWL group had significantly more cases with severe hydronephrosis ($p = 0.041$). The number of cases with chronic pain was higher in the delayed SWL group ($p = 0.015$). Both groups were similar in terms of shock numbers, amount of energy and pain scores (Table 2). In the delayed SWL group, one (1.6 %) patient passed his stone spontaneously. Total number of SWL sessions was 1, 2, and 3 in 43, 7 and 12 cases, respectively (Table 2). The average size of residual stones was 5.6 ± 2.3 and 7.9 ± 3.4 mm ($p = 0.053$) after first session in the immediate SWL and delayed SWL groups, respectively. 17 (53.1 %) and 12 (40 %) patients ($p = 0.353$) were stone free after the first session in the immediate SWL and the delayed SWL groups, respectively (Table 3). When CIRF was also considered as success, the success rate of SWL in the immediate SWL group was higher after first session ($p = 0.002$). After the first session, according to the stone location, it was observed that immediate SWL had a greater success rate in upper ureteric stones ($p = 0.019$), while the treatment success was similar between both the regimens in other parts of the ureter ($p = 0.414$ for mid and $p = 0.071$ for lower ureteric stones) (Table 4).

Average stone sizes requiring one, two and three SWL sessions were 7.52 ± 2.08, 8.57 ± 2.29 and 12.04 ± 3.67 mm, respectively, in both groups ($p < 0.001$). Patients undergoing at least two sessions of treatments in the immediate SWL group had bigger stones than their counterparts in the delayed SWL group ($p < 0.01$). Although more sessions were required for patients in the delayed SWL group, no statistical significance was observed between the groups (independent groups *t* test $p = 0.068$).

When the presence of CIRF is considered as success, SWL success rate in the immediate treatment group increased to 93.8 % after three sessions, whereas it was 80 % in the delayed SWL group ($p = 0.107$). In general, SWL success rate in ureteral stones was 69.4 % after first session and 87.1 % after three sessions when CIRFs were also included. Overall average time required for elimination of stones was 6.31 ± 8.84 (1–39) days in immediate SWL group and 9.82 ± 10.24 (1–37) days in delayed SWL group, and the difference was significant ($p = 0.033$).

The success rate after first SWL session (including CIRF) was evaluated according to the pain type at referral, which was found to be 59 % for patients with chronic pain in the delayed group, whereas it was 100 % for patients with acute pain in the immediate group. When the first session success rates were evaluated (including CIRF) according to hydronephrosis grade, success rates were 71 and 44.4 % for patients with grade 1 or no hydronephrosis in the immediate SWL group, and for patients with grade 2 or larger hydronephrosis in the delayed SWL group, respectively.

On the other hand, URS lithotripsy was performed as an additional procedure after three SWL sessions in 8 (12.7 %) cases; 2 (6.2 %) from the immediate SWL group and 6 (19.4 %) from the delayed SWL group. Two groups were similar in terms of the necessity for an additional intervention ($p = 0.472$).

Discussion

SWL is preferred in the treatment of most of the kidney and ureteral stones due to its simplicity, noninvasiveness, effectiveness, minimum morbidity and no need for anesthesia. For a significant portion of ureteric stones, SWL is the first treatment option, which is recommended in the Ureteral Calculi Guidelines of both the European

Table 1 Breakdown of cases by gender, age, height, body weight, BMI and size of stone

	Immediate SWL group	Delayed SWL group	Total/average value	*p*
Number *n* (%)	32 (50.8)	31 (49.2)	63 (100)	
Gender M/F *n* (%)	25(78.1)/7(21)	25(80.6)/6(19.4)	50 (79.4)/13 (20.6)	0.526
Age (year)[a]	36.7 ± 12.7	37.6 ± 12.8	37.2 ± 12.7	0.797
Height (cm)[a]	172.7 ± 10.2	172.5 ± 9.04	172 ± 9.58	0.934
Weight (kg)[a]	78.6 ± 15.7	78.09 ± 12.8	78.3 ± 14.2	0.878
BMI (kg/m^2)[a]	26.4 ± 5.59	26.2 ± 3.7	26.3 ± 4.7	0.857
Size of stone (mm)[a]	8.12 ± 3.16	8.8 ± 2.87	8.46 ± 3.02	0.375

BMI body mass index

[a] Figures are provided as follows: average ± standard deviation

Table 2 Stone locations, laterality, grade of hydronephrosis, pain type, number of shock waves, amount of energy, pain score, nember of treatment sessions according to treatment groups

	Immediate		Delayed	
	N	%	*N*	%
Laterality				
Right	14	43.8	9	36.5
Left	18	56.2	22	65.5
Location				
Upper	21	65.6	20	64.5
Middle	2	6.2	4	12.9
Lower	9	28.1	7	22.5
Hydronephrosis				
0	3	9.4	1	3.2
1	18	56.2	12	38.7
2	11	34.4	12	38.7
3	–	0	6	19.4
Pain				
Acute	19	59.4	9	29
Chronic	13	40.6	22	71
Shock number				
1st session	3,187 ± 595		3,187 ± 661	
2nd session	3,416 ± 204		3,349 ± 311	
3rd session	2,875 ± 629		3,375 ± 353.5	
Amount of energy applied (Joules)				
1st session	70.8 ± 36.4		71 ± 31.4	
2nd session	105 ± 34.6		85.8 ± 36.6	
3rd session	76.1 ± 43.7		92 ± 27.4	
Pain score				
1st session	4.56 ± 1.9		4.7 ± 2.1	
2nd session	4.3 ± 2.8		4.6 ± 2.9	
3rd session	3.75 ± 2.36		4.12 ± 3.44	
Number of SWL session				
1st session	26	81.2	17	56.6
2nd session	2	6.2	5	16.6
3rd session	4	12.5	8	26.6

Table 3 Success rates after first SWL treatment according to groups

Third day after first SWL session	Immediate SWL *n* (%)	Delayed SWL *n* (%)	*p**	*p***
Stone-free	17 (53.1)	12 (40.0)	0.353	0.105
CIRF	9 (28.1)	5 (16.7)	0.285	
Failure	6 (18.8)	13 (43.3)	0.108	

* Chi-square test, ** Kruskal–Wallis test

Association of Urology (EAU) and American Urological Association (AUA) [4].

It is known that impacted ureteric stones are more resistant to SWL [14]. An impacted stone causes ureteral edema within 24–48 h [6, 17, 18]. It has been observed in histopathologic evaluations that the mucosa, which the stone is in contact with, gains a hyperplastic appearance within 48 h due to the increased mitosis [18]. Ureteral edema narrows the expansion area that is required for lithotripsy and removes the fluid between the stone and the tissue [7]. For this reason, it seems logical to perform SWL within 24 h before ureteral edema develops. We aimed to study whether a SWL procedure is advantageous before edema develops in the ureter by evaluating the success of the first SWL session in particular on the third day after the procedure. Furthermore, the success rate of SWL after three sessions was also evaluated to see the effectiveness of immediate SWL in time.

Ureteral calculi smaller than 5 mm can pass spontaneously, and an expectant follow-up is a reasonable approach

Table 4 After first session, success rate of immediate and delayed SWL

	Immediate SWL		Delayed SWL		*p*
	N	%	*N*	%	
Success	26	81.2	17	56.7	0.002
Failure	6	18.8	13	43.3	
Stone location					
Upper ureter	17/21	80.9	11/20	55	0.019
Middle ureter	1/2	50	3/4	75	0.414
Lower ureter	8/9	88.8	3/6	50	0.071

for these stones. Spontaneous passage rate decreases significantly when the stone diameter exceeds 4 mm, i.e, 48 and 25 % for stones with diameters 7–9 mm and greater than 9 mm, respectively [5]. Stones in our study were bigger than 4 mm. The fact that only one patient out of 31 in the delayed group passed his stone spontaneously supports the idea that expectant follow-up for ureteral stones bigger than 4 mm may not be beneficial, and immediate SWL may be a better approach.

It is known that stone size is an important parameter in the success rate of SWL, which differs significantly between ureteral stones smaller and bigger than 1 cm [19, 20]. Our study also shows that as stone size gets bigger, the number of SWL sessions, which are required to eliminate the stone, increases as well. Stone-free rate in the third month after SWL is 82 % for upper ureteral stones, 73 % for middle ureteral stones, and 74 % for lower ureteral stones according to the AUA and EAU guidelines [5]. In our study, the average stone-free rate within the first 1.5-month period (1–41 days) for 43 cases required only one SWL session was statistically higher in the immediate SWL group independent from stone size ($p = 0.037$). When SWL success after one SWL session was assessed according to stone location, the success rate was higher for upper ureteral stones in the immediate SWL group ($p = 0.019$). On the other hand, the success rate was similar between the two treatment groups in middle ($p = 0.414$) and lower ($p = 0.071$) ureteral stones, which might be due to the insufficient number of cases for subgroup analysis.

There are few studies evaluating the outcome of immediate SWL in ureteral stones. In some of the studies, only the patients with upper ureteral stones were evaluated [12–14], while in others, stones in all locations of the ureter were studied [1, 9, 11], as in our study. The methodology of each study was different; Tombal et al. compared SWL to medical treatment, while Seitz compared patients with colicky pain to those that do not have colicky pain [12]. Tombal et al. [11] reported that they performed an immediate SWL procedure within the first 6 h from the onset of the pain, which proved to be more successful than medical treatment in patients with upper ureteral stones . It has been found that stone-free rate was higher and hospitalization time was shorter with the use of immediate SWL procedure for upper ureteral stones. Seitz et al. [12] found stone clearance time accelerated when immediate SWL was applied. Our findings are compatible with these results. Patients with upper ureteral stones and colicky pain who underwent a SWL procedure within first 24 h of referral were compared to those with no colicky pain who underwent an elective SWL procedure 5 days after referral. Stone clearance time and success rate were similar and independent of concomitant hydronephrosis [13]. Kumar et al. [14] reported that disintegration rate and time to stone-free status was longer, though statistically insignificant, when SWL was performed later than 48 h after the onset of pain in their randomized study including symptomatic patients with single 1 cm or larger radioopaque upper ureteral stones. They also found that the number of SWL sessions and the need for additional interventions increased with time [14]. Our study showed that stone clearance time was longer in the delayed SWL group ($p = 0.033$) and there was an increasing need for additional procedures (19.4 %). Furthermore, our study suggests that stone-free rate after first session was significantly lower in cases with chronic pain and included in the delayed SWL treatment group when compared with those in immediate SWL group. Residual stone size was smaller in the immediate SWL group on the third day after first session, which was close to statistical significance ($p = 0.053$). Time to stone clearance was shorter in 26 patients undergoing immediate SWL out of total 43 cases requiring only one SWL session, as well as it was also shorter in immediate SWL group after three sessions, though statistically insignificant. Success and failure rates were similar after three sessions while immediate SWL appeared more successful after first session, and this suggests that SWL should be performed in the early period in patients with colicky pain due to ureteral stones. From this viewpoint, patients from the immediate SWL group who did not pass their stones had a similar clinical picture to those patients from the delayed SWL group during follow-up.

In some of the previously published studies, it has been reported that the presence and the grade of hydronephrosis determined with ultrasonography before SWL have no impact on elimination of stones and treatment outcome [13, 16–20]. Similarly, it has been found out that hydronephrosis has no impact on stone disintegration or the time to stone-free status ($p = 0.769$) in our study. On the other hand, a borderline relationship ($p = 0.051$) between grade of hydronephrosis and need for SWL sessions has been detected. This indicates that a higher number of interventions are needed for the elimination of impacted stones.

Although no significant relationship has been determined between the level of hydronephrosis and SWL success ($p = 0.454$), it was observed that as the level of hydronephrosis increased, the number of required SWL sessions in order to achieve the same outcome increased. Hydronephrosis has been identified more frequently in the delayed SWL group. When all the patients were separated into four groups according to hydronephrosis grade and treatment time (immediate or delayed), success after first session was the least in patients with severe hydronephrosis and underwent a delayed SWL procedure. This seems to support that hydronephrosis grade is related with an impacted stone.

The necessity for post-SWL additional procedure (URS) for ureteral stones has been reported as 5.5 % by Tligui et al. [21], 21 % by Tombal et al. [11] and 19.8 % by Seitz et al. [18]. In our study, additional procedures were required in only 12.7 % of the cases and it has been found that additional procedures are required in patients from the delayed SWL group, though this was statistically insignificant. Immediate SWL reduces the need for additional sessions, while it seems that it also reduces the need for post-SWL additional procedures. To further support this statement, other studies including higher number of cases should be performed.

Conclusions

Immediate SWL insures stone-free status in a shorter time in cases with renal colicky pain and ureteral stones, particularly upper ureteral stones. Moreover, it reduces the need for additional SWL sessions and additional procedures (URS). In determining the success of immediate SWL, evaluating the cases on the third day after first SWL session is important since those cases requiring a second session follow a clinical course like the cases in the delayed SWL group because of developing ureteral edema. Our findings suggest that immediate SWL is useful in insuring stone-free status in the shortest time possible and/or in deciding whether additional procedures are necessary for cases referring with renal colicky pain and ureteral stones.

References

1. Ghalayini I, Al-Ghazo M, Khader Y (2008) Evaluation of emergency extracorporeal shock wave lithotripsy for obstructing ureteral stones. Int Braz J Urol 34:433–442
2. Segura JW, Preminger GM, Assimos DG et al (1997) Ureteral Stones Clinical Guidelines Panel summary report on the management of ureteral calculi. The American Urological Association. J Urol 158:1915–1921
3. Tombolini P, Ruoppolo M, Bellorofonte C et al (2000) Lithotripsy in the treatment of urinary lithiasis. J Nephrol 13(Suppl 3):S71–S82
4. Preminger GM, Tiselius HG, Assimos DG (2007) Guideline for the management of ureteral calculi. Eur Urol 52:1610–1631
5. Azm TA, Higazy H (2002) Effect of diuresis on extracorporeal shockwave lithotripsy treatment of ureteric stones. Scand J Urol Nephrol 36:209–212
6. Mueller SC, Wilbert D, Thueroff JW et al (1986) Extracorporeal shock wave lithotripsy of ureteral stones: clinical experience and experimental findings. J Urol 135:831–834
7. Parr NJ, Pye SD, Ritchie AWS et al (1992) Mechanisms responsible for diminished fragmentation of ureteral calculi: an experimental and clinical study. J Urol 148:1079–1083
8. Doublet JD, Tchala K, Tligui M et al (1997) In situ extracorporeal shock wave lithotripsy for acute renal colic due to obstructing ureteral stones. Scand J Urol Nephrol 31:137–139
9. Joshi HB, Obadeyi OO, Rao PN (1999) A comparative analysis of nephrostomy, JJ stent and urgent in situ extracorporeal shock wave lithotripsy for obstructing ureteric stones. BJU Int 84:264–269
10. Kravchick S, Bunkin I, Stepnov E et al (2005) Emergency extracorporeal shockwave lithotripsy for acute renal colic caused by upper urinary-tract stones. J Endourol 19:1–4
11. Tombal B, Mawlawi H, Feyaerts A et al (2005) Prospective randomized evaluation of emergency extracorporeal shock wave lithotripsy (ESWL) on the short-time outcome of symptomatic ureteral stones. Eur Urol 47:855–859
12. Seitz CFH, Remzi M, Waldert M et al (2006) Rapid extracorporeal shock wave lithotripsy treatment after a first colic episode correlates with accelerated ureteral stone clearance. Eur Urol 49:1099–1105
13. Seitz C, Tanovic E, Kikic Z et al (2007) Rapid extracorporeal shock wave lithotripsy for proximal ureteral calculi in colic versus noncolic patients. Eur Urol 52:1223–1227
14. Kumar A, Mohanty NK, Jain M et al (2010) A prospective randomized comparison between early (<48 h onset of colicky pain) versus delayed shockwave lithotripsy for symptomatic upper ureteral calculi: a single center experience. J Endourol 24(12): 2059–2066
15. Kriegshauser JS, Carroll BA (1991) The urinary tract. In: Rumack CM, Wilson SR, Charboneau JW (eds) Diagnostic ultrasound, Mosby Year Book, St.Louis, p 242
16. Robert M, Delbos O, Guiter J et al (1995) In situ piezoelectric extracorporeal shock wave lithotripsy of ureteric stones. Br J Urol 76:435–439
17. Farsi HM, Mosli HA, Alzimaity M (1994) In situ extracorporeal shock wave lithotripsy for primary ureteric calculi. Urology 43:776–781
18. Seitz C, Fajkovic H, Waldert M et al (2005) Extracorporeal shock wave lithotripsy in the treatment of proximal ureteral stones: does the presence and degree of hydronephrosis affect success? Eur Urol 49:378–383
19. Demirbas M, Kose AC, Samli M et al (2004) Extracorporeal shock wave lithotripsy for solitary distal ureteral stones: does the degree of ureteral obstruction affect success? J Endourol 18:237–240
20. Zagoria RJ, Khatod EG, Chen MYM (2001) Abdominal radiography after CT reveals urinary calculi: a method to predict usefulness of abdominal radiography on the basis of size and CT attenuation of calculi. AJR 176:1117–1122
21. Tligui M, El Khadime MR, Tchala K et al (2003) Emergency extracorporeal shock wave lithotripsy (ESWL) for obstructing ureteral stones. Eur Urol 43:552–555
